# Cardiovascular Autonomic Neuropathy in Type 1 Diabetes Is Associated With Disturbances in TCA, Lipid, and Glucose Metabolism

**DOI:** 10.3389/fendo.2022.831793

**Published:** 2022-04-14

**Authors:** Christian S. Hansen, Tommi Suvitaival, Simone Theilade, Ismo Mattila, Maria Lajer, Kajetan Trošt, Linda Ahonen, Tine W. Hansen, Cristina Legido-Quigley, Peter Rossing, Tarunveer S. Ahluwalia

**Affiliations:** ^1^Complications Research, Steno Diabetes Center Copenhagen, Herlev, Denmark; ^2^Department of Clinical Medicine, University of Copenhagen, Copenhagen, Denmark; ^3^The Department of Medicine, Herlev-Gentofte Hospital, Copenhagen, Denmark; ^4^The Bioinformatics Center, Department of Biology, University of Copenhagen, Copenhagen, Denmark

**Keywords:** cardiovascular autonomic neuropathy, lipidomics, proteomics, TCA cycle, citric acid - CA

## Abstract

**Introduction:**

Diabetic cardiovascular autonomic neuropathy (CAN) is associated with increased mortality and morbidity. To explore metabolic mechanisms associated with CAN we investigated associations between serum metabolites and CAN in persons with type 1 diabetes (T1D).

**Materials and Methods:**

Cardiovascular reflex tests (CARTs) (heart rate response to: deep breathing; lying-to-standing test; and the Valsalva maneuver) were used to diagnose CAN in 302 persons with T1D. More than one pathological CARTs defined the CAN diagnosis. Serum metabolomics and lipidomic profiles were analyzed with two complementary non-targeted mass-spectrometry methods. Cross-sectional associations between metabolites and CAN were assessed by linear regression models adjusted for relevant confounders.

**Results:**

Participants were median (IQR) aged 55(49, 63) years, 48% males with diabetes duration 39(32, 47) years, HbA_1c_ 63(55,69) mmol/mol and 34% had CAN. A total of 75 metabolites and 106 lipids were analyzed. In crude models, the CAN diagnosis was associated with higher levels of hydroxy fatty acids (2,4- and 3,4-dihydroxybutanoic acids, 4−deoxytetronic acid), creatinine, sugar derivates (ribitol, ribonic acid, myo-inositol), citric acid, glycerol, phenols, phosphatidylcholines and lower levels of free fatty acids and the amino acid methionine (p<0.05). Upon adjustment, positive associations with the CAN diagnoses were retained for hydroxy fatty acids, tricarboxylic acid (TCA) cycle-based sugar derivates, citric acid, and phenols (P<0.05).

**Conclusion:**

Metabolic pathways, including the TCA cycle, hydroxy fatty acids, phosphatidylcholines and sugar derivatives are associated with the CAN diagnosis in T1D. These pathway may be part of the pathogeneses leading to CAN and may be modifiable risk factors for the complication.

## Introduction

People with diabetes are prone to complications including autonomic dysfunction. The prevalence of cardiovascular autonomic neuropathy (CAN) ranges from 20% in unselected populations with diabetes (1–3) to 35-65% in people with long-standing diabetes (4). CAN is an independent predictor of cardiovascular mortality and morbidity (5–10), diabetic nephropathy (11–14) and disorders of bone metabolism (15). For decades strides have been taken to compose a treatment for CAN. However, despite the severity of CAN no disease modifying treatment exist (16). The exploration of novel risk factors is therefore warranted. Such explorations can be facilitated by using non-targeted investigations of the metabolome and lipidome. Indeed, several studies based on metabolomic serum analyses have presented insights into the pathophysiology associated with the severity and progression of non-neuropathic complications including diabetic nephropathy (17–20) and retinopathy (21). Recently, metabolomic investigations have unveiled new insights into associations between disturbances in energy metabolism and CAN in a smaller study (n=47). Here, autonomic dysfunction and components of the tricarboxylic acid (TCA) cycle (22) were correlated. This indicates that CAN is associated with changes in human energy metabolism. Our aim for the current study is to explore potential associations between metabolic pathways including components of the TCA cycle and CAN by investigating circulating metabolites and CAN in persons with type 1 diabetes.

## Materials and Methods

### Study Population

This cross-sectional study includes people with type 1 diabetes recruited to a study performed at Steno Diabetes Center Copenhagen (SDCC) in Denmark from 2009 to 2011, as described previously (23). Participants were recruited from a cohort of participants in a case-control study (conducted in 1993-2001) including 900 persons with either longstanding normoalbuminuria or diabetic nephropathy. 571 participants of the 900 participants were still alive for this cross-sectional study. A total of 375 participants responded to the study invitation. Of these 20 were not eligible for inclusion because of severe comorbidities, such as cancer and non-diabetic kidney disease, leaving a total of 355 participants. Twenty-six participants were outside the age range of 20–80 years and were not included in the present study because reference values of CAN measures outside this age range do not exist, leaving 329 participants with usable CAN measures. Of these 329 participants, 27 had either missing data on omics outcomes or covariates, leaving 302 participants for analyses. An estimation of the CAN diagnosis was possible for 258 participants. Forty-four participants did not have enough data to enable CAN estimation due to lack of sufficient CAN measures caused by a participant’s inability to perform the respective tests or because of artefacts in the recordings caused by movement or electrical interference.

All participants gave written informed consent. The study adhered to the Declaration of Helsinki and the study protocol was approved by the local ethics committee; ClinicalTrials.gov ID NCT01171248.

### Assessment of Autonomic Function

Participants rested for 5 minutes in a supine position in a quiet room at a temperature between 18-23 degrees Celsius prior to the assessment of autonomic function.

Autonomic function was assessed by measures of CAN by 2-minute resting heart rate variability (HRV) indices and cardiovascular autonomic reflex test (CARTs). The HRV measure calculated for the present study was the standard deviation of normal-to-normal (SDNN) intervals which was derived from resting R-R-intervals. After the HRV measurement, the three standard CARTs recommended for diagnosing CAN (24) were performed: the lying-to-standing test (30/15), the deep breathing test (E/I ratio) and the Valsalva maneuver.

CARTs and SDNN measures were analyzed as continuous variables. Moreover, age-dependent cut-off levels defined by Cardone et al. (25) were used to define pathological results of the CARTs. The CAN diagnosis was defined as the presence of two or three pathological CARTs as recommended by the American Diabetes Association (26) and classified for participants with more than one valid CART measure. Participants with one or no CARTs measures were classified as “no CAN estimation”. Higher values of the CAN measures imply better autonomic function, whereas higher resting heart rate imply worse autonomic function.

Resting heart rate (HR), SDNN and CARTs were recorded by trained technicians using a Vagus™ device (Medicus Engineering, Aarhus, Denmark).

### Metabolomics Analyses

#### Sample Quantification and Identification

The metabolomics and lipidomic analysis have been described in detail previously (27, 28). Serum samples were stored at −80◦C until analyzed by two different analytical methods. Metabolomics profiling of samples was performed using two-dimensional gas chromatography with time of flight mass spectrometry. Peaks were identified from the raw data with ChromaTOF, and the resulting features aligned with Guineu.

Samples for lipidomics analysis were prepared using a modified Folch extraction procedure and analyzed by ultra-high-performance liquid chromatography quadruple time of flight mass spectrometry method. Raw acquired data was preprocessed with MZmine 2. A complete list of processed metabolites (including amino acids, free fatty acids, compounds from the energy metabolism pathways and polyols) is available in Tofte et al. (27). Lipid species have been defined by the number of carbon atoms (indicating total fatty acid chain length) and the number of double bonds for a specific species. These identities have been presented as “Class (number of carbon atoms:number of double-bonds)”.

The inclusion of metabolites and lipids (within the coverage of the two mass spectrometry platforms) in subsequent data analysis was solely based on the certainty of identification and the level of technical precision, thereby not restricted to any specific pathway or prior hypothesis.

#### Baseline Biochemical Measures

HbA_1c_ was measured by high-performance liquid chromatography (Variant, Bio-Rad Laboratories, Munich, Germany) and serum creatinine concentration by an enzymatic method (Hitachi 912; Roche Diagnostics, Mannheim, Germany). Urinary albumin excretion ratio was measured in three 24-hour urine collections by enzyme immunoassay. Chronic Kidney Disease Epidemiology Collaboration Equation was used to calculate the estimated glomerular filtration rate (eGFR) from serum creatinine.

#### Anthropometric Measures

Height and weight were measured with light indoor clothing, without shoes, using a fixed rigid stadiometer (Seca, Chino, USA) and an electronic scale (Mettler Toledo, Glostrup, Denmark), respectively.

#### Blood Pressure

Oscillometric (A&D Medical, UA787) office blood pressure was measured in a supine position after 15 minutes of rest using an appropriate cuff size. Three measurements were obtained and averaged.

#### Lifestyle Measures

Lifestyle measures were obtained by questionnaires. Participants were classified as current smokers if using ≥ 1 cigarette or cigar or pipes per day and all others were classified as non-smokers.

### Statistical Analyses

Continuous variables were reported as median and interquartile range (IQR). Skewed data were log2-transformed for analyses. Categorical variables were presented as total numbers with corresponding percentages. Comparisons of continuous and categorical variables between groups (no CAN *vs.* CAN) were performed using Wilcoxon rank-sum test and X^2^-test, respectively. Data were imputed and auto-scaled prior to model-fitting. Associations between clinical characteristics of interest (the CAN diagnosis and CAN indices) and the levels of individual compounds were assessed with compound-specific linear regression models adjusted to clinical variables. Using the R package limma as described previously (28). All analyses were performed using three levels of confounder adjustment: crude model: no adjustment; adjusted model: adjusted for age, sex, fasting plasma glucose, HbA_1c_, body mass index, diabetes duration, smoking, statin use, total cholesterol and total triglycerides. To explore the effect of kidney function on associations a third model (fully adjusted model) was applied with additional adjustment for eGFR. Due to 41 missing values of urinary albumin excretion rate (mg/24-hour) additional adjustments for albuminuria was not performed. Results from regression analyses were visualized as forest plots, bipartite graphs and heatmaps using the lipidomeR package.

P-values for each analysis were corrected for multiple testing using the Benjamini–Hochberg method and have been denoted as “q” in the main and **Supplementary Figures**. Significant associations between clinical variables and circulating metabolites levels from crude models were integrated into a single visualization as a chord diagram using R-package circlize.

All data analyses were completed with R version 4.0.4 (The R Foundation for Statistical Computing, www.r-project.org).

## Results

Three-hundred-and-two participants with type 1 diabetes were included in the final analyses. These were participants with any CARTs or HRV measured. They had a median (IQR) aged of 55 (49;63) years, 48% were males, with a diabetes duration of 39 (32;47) years, HbA_1c_ 63 (55;69) mmol/mol, eGFR 83 (60;98) ml/min/1.73m^2^. A total of 258 had a sufficient number of CARTs recorded to estimate the presence of the CAN diagnosis. A total of 88 (34%) persons were diagnosed with CAN. Group differences between persons without *vs.* with CAN are displayed in **Table 1**.

**Table 1:** Clinical Characteristics.

	No CAN, N = 170	Definite CAN, N = 88	No CAN estimation N=44	p-value (no CAN *vs*. CAN)
Sex (male), (n/%)	76 (46)	45 (54)	24 (57)	0.3
Age (years)	55 (48, 62)	55 (49, 60)	60 (52, 66)	0.7
HbA_1c_ (mmol/mol)	62 (55, 67)	64 (59, 73)	61 (53, 69)	0.002
HbA_1c_ (%)	7.80 (7.20, 8.30)	8.05 (7.57, 8.83)	7.70 (7.03, 8.47)	0.002
Body mass index (kg/m2)	24.2 (22.2, 26.9)	24.0 (22.3, 26.2)	23.8 (21.9, 26.1)	0.7
Systolic blood pressure (mmHg)	128 (118, 141)	131 (120, 147)	130 (114, 137)	0.10
Diastolic blood pressure (mmHg)	73 (68, 79)	73 (68, 80)	74 (68, 78)	>0.9
Diabetes duration (years)	37 (31, 45)	40 (35, 48)	43 (38, 51)	0.026
Smoking (n%)	26 (16%)	20 (24%)	10 (24%)	0.2
eGFR (ml/min/1.73 m^2^)	87 (75, 101)	62 (43, 84)	83 (61, 104)	<0.001
Urinary albumin excretion rate (mg/24-hour)	10 (6, 23)	56 (14, 432)	11 (6, 54)	<0.001
Total cholesterol (mmol/L)	4.55 (4.18, 5.10)	4.50 (4.00, 4.90)	4.55 (4.12, 5.10)	0.10
HDL cholesterol (mmol/L)	1.69 (1.39, 2.08)	1.54 (1.27, 1.86)	1.67 (1.39, 2.09)	0.013
LDL cholesterol (mmol/L)	2.30 (1.90, 2.73)	2.30 (1.90, 2.70)	2.30 (2.00, 2.95)	0.9
Triglycerides (mmol/L)	0.89 (0.68, 1.24)	0.98 (0.80, 1.23)	0.81 (0.67, 1.07)	0.048
Diuretic (n/%)	65 (40)	68 (81)	22 (52)	<0.001
RAAS blocker (n/%)	68 (42)	7 (8.3)	15 (36)	<0.001
Statins (n/%)	90 (55)	67 (80)	21 (50)	<0.001
Pathological E/I ratio (n/%)	27 (1)	82 (98)	21 (64)	<0.001
Pathological 30/15 ratio (n/%)	14 (8,7)	74 (88)	6 (21)	<0.001
Pathological Valsalva (n/%)	4 (3)	42 (79)	1 (33)	<0.001
E/I ratio	1.17 (1.12, 1.28)	1.04 (1.02, 1.07)	1.10 (1.05, 1.16)	<0.001
30/15 ratio	1.10 (1.06, 1.17)	1.01 (1.00, 1.03)	61 (53, 69)	<0.001
Valsalva maneuver	1.52 (1.36, 1.78)	1.16 (1.11, 1.27)	1.48 (1.32, 1.64)	<0.001
SDNN (ms)	30 (20, 42)	10 (7, 15)	20 (13, 33)	<0.001
Resting heart rate (beats/minute)	65 (58, 73)	74 (68, 84)	68 (59, 78)	<0.001

Data are in medians (IQR) or n (%). eGFR, Estimated glomerular filtration rate (ml/min/1.73m2); RAAS, Renin angiotensin aldosterone system; CAN, cardiovascular autonomic neuropathy. Wilcoxon rank sum test; Chi-squared test.

### Metabolomic Analysis

A total of 75 serum metabolites and 106 lipid species were identified and passed quality control (**Supplementary List 1**)

### Result Overview of Crude(Unadjusted) Models

**Figure 1:**
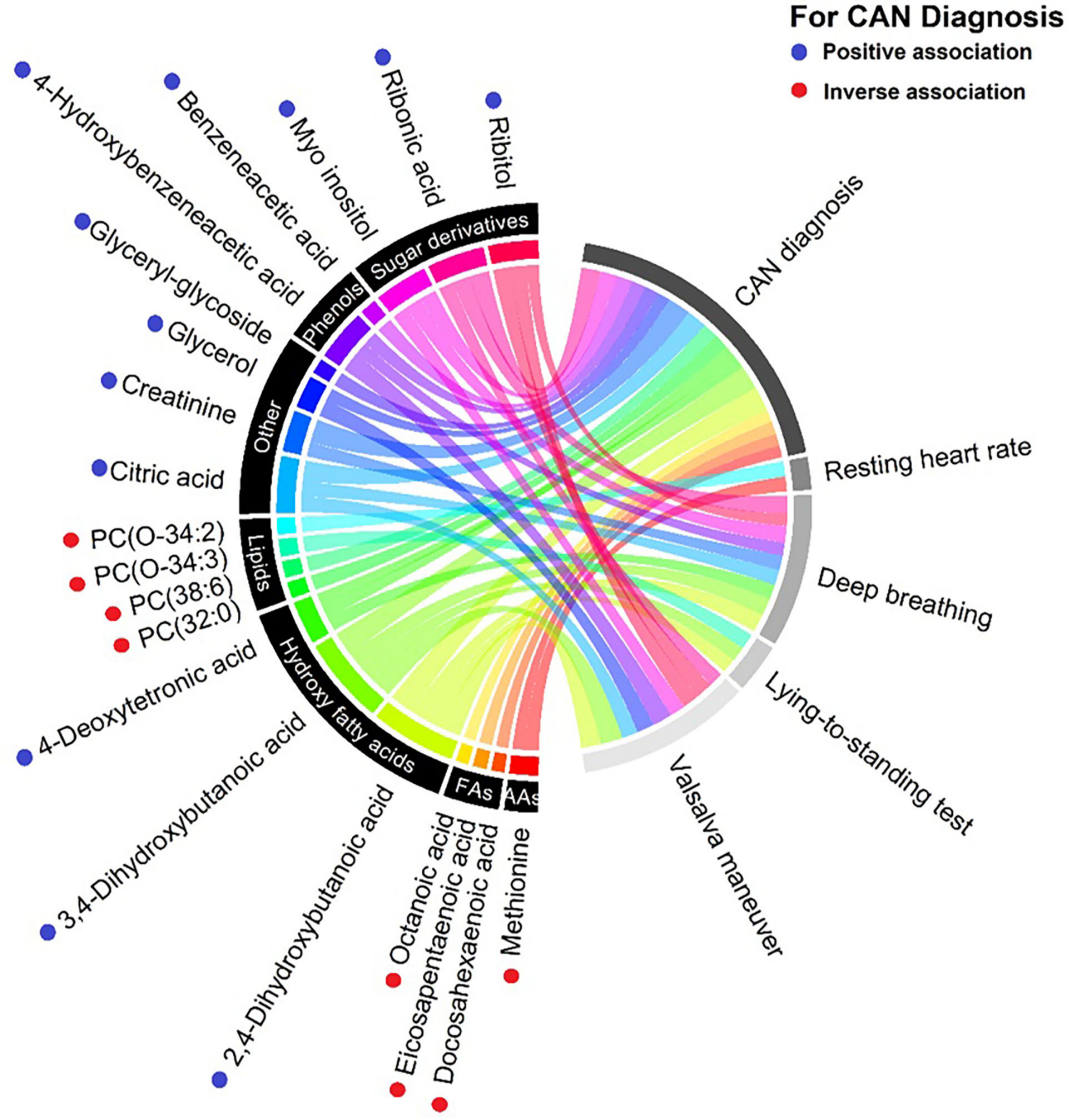
Chord diagram of the detected associations between metabolites (left) and the cardiovascular autonomic neuropathy (CAN) diagnosis and specific CAN measures (right) from the crude(udadjusted) model. Metabolites are categorized into pathways and shown with unique colors. Line width indicate strenght of the respective assocation. PC, Phosphatidylcholines; FAs, Fatty acids; AAs, Amino acids. Blue dots indicate higher levels of oucomes in patients with CAN compared with no CAN. Red dots indicate lower levels of outcomes in patients with CAN compared with no CAN.

**Figure 2:**
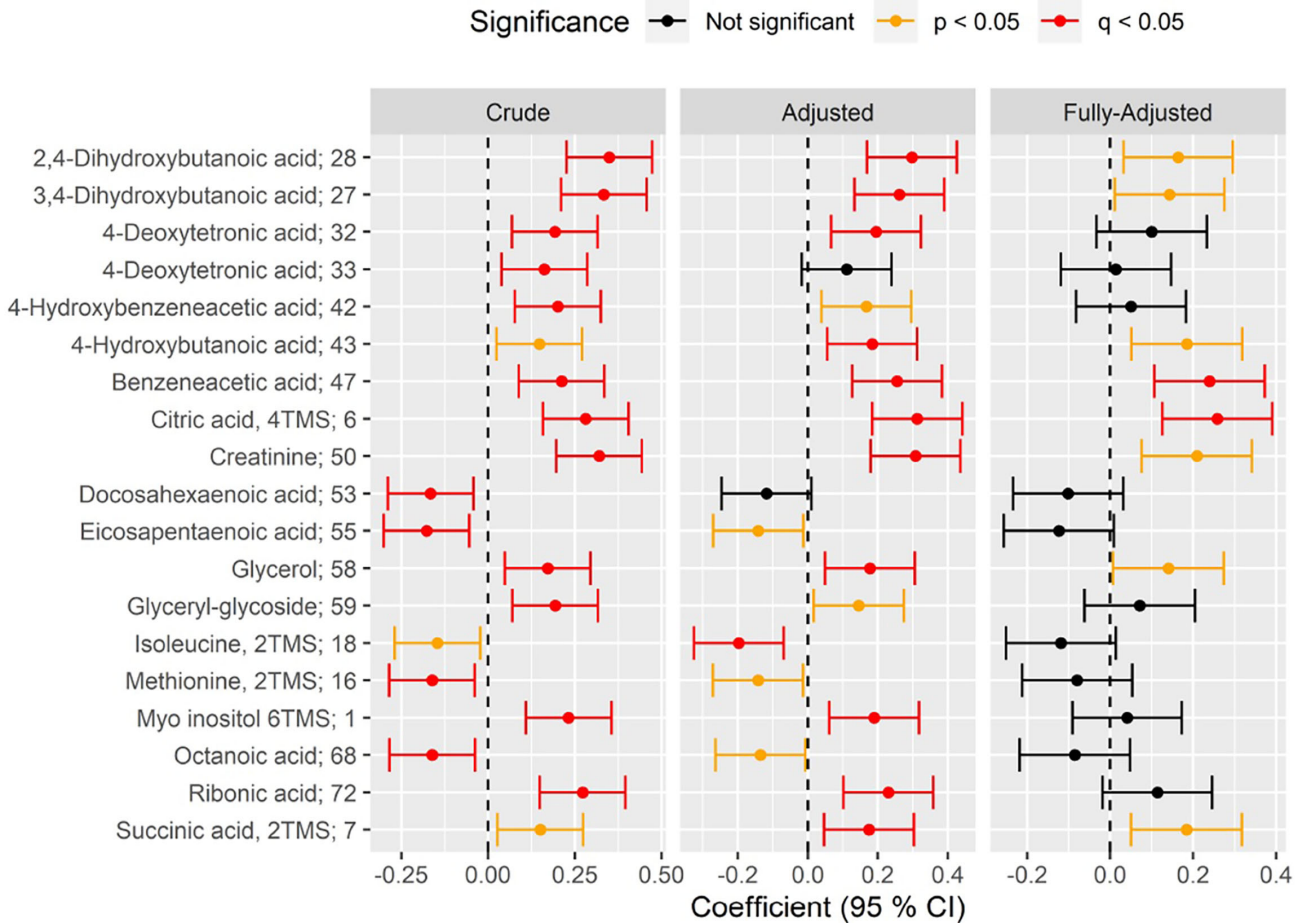
Forest plot of standardized association between CAN *vs* no CAN and circulating metabolite level in crude (left), adjusted (middle) and fully-adjusted (right) metabolite-specific regression models (rows). Positive (negative) coefficient on the x-axis indicates positive (inverse) association between CAN and metabolite level. Statistical significance of the association is indicated by color of the confidence 95% interval (red: significant after correction to multiple testing(q); orange: significant nominal p-value(p); black: not significant). Results are shown for crude models (unadjusted; left), for models adjusted for age, sex, plasma glucose, HbA_1c_, body mass index, diabetes duration, smoking, statin use, total cholesterol and total triglycerides (“Adjusted”; middle) and models further adjusted for eGFR (“Fully-Adjusted”, right). All significant associations (q<0.05) in at least one of the three models, have been indicated.

16 circulating metabolites were associated with CAN in the crude model (**Figures 1** and **2**, **Supplementary Appendix 1, Section 3.1.1**; p<0.05). Here, participants with CAN had higher levels of hydroxy fatty acids (2,4- and 3,4-dihydroxybutanoic acids, 4−deoxytetronic acid), creatinine, sugar derivates (ribitol, ribonic acid, myo-inositol), citric acid, glycerol, phenols, phosphatidylcholines and lower levels of free fatty acids and amino acid methionine compared to participants without CAN. The majority of the associations to CARTs were seen for the E/I ratio (**Supplementary Appendix 1 Section 5.2.1**) and the Valsalva maneuver. Higher resting heart rate was associated with a lower methionine level (**Supplementary Appendix 1 Section 5.1.1**). SDNN was not associated with any metabolites (**Supplementary Appendix 1 Section 5.5**; p>0.05). Below are results described in detail for categories of outcomes for all levels of adjustment.

### Result According to Outcome Categories

#### Hydroxy Fatty Acids

The CAN diagnosis was associated with higher levels of three hydroxy fatty acids in the adjusted model: 2,4- (beta=0.29, p=1.4×10^-4^) and 3,4-dihydroxybutanoic acids (beta=0.26, p=1.2×10^-3^), 4−deoxytetronic acid (beta=0.19, p=0.027; **Figure 2** and **Supplementary Appendix 1 Section 3.2**). Associations remained significant after additional adjustment for eGFR (**Figure 2** and **Supplementary Appendix 1 Section 3.3**; p<0.05).

Higher E/I ratio was associated with higher levels of all three above mentioned hydroxy fatty acids in crude models (**Supplementary Appendix 1 Section 5.2.2**; p<0.05) but only with lower levels of ribonic and citric acids in the adjusted model (**Supplementary Appendix 1 Section 5.2.2**; p<0.05). Higher levels of the Valsalva maneuver were associated (p<0.05) with higher levels of 2,4- and 3,4-dihydroxybutanoic acids, but not 4−deoxytetronic acid in crude models. Associations were lost with further adjustments.

No other CAN measure including resting heart rate and SDNN were associated with hydroxy fatty acids (**Supplementary Appendix 1**, p >0.05).

#### TCA Cycle

In the adjusted model, CAN diagnosis was associated with higher levels of citrate (beta: 0.31, p=1.0×10^-4^), but no other metabolites of the TCA cycle. This association remained significant after additional adjustment for eGFR (beta= 0.25, p=0.01, **Figure 2**, **Supplementary Appendix 1 Section 3.3** and **Figure 3.4**).

Higher values of the E/I ratio and the Valsalva were associated with lower levels of citrate in crude models(beta=-0.21, p=5.1×10^-3^), and the E/I ratio retained significant associations in the adjusted model(p<0.05).

No other associations between CAN measures and the TCA-cycle intermediates were found (**Supplementary Appendix 1**).

#### Sugar Derivatives

In crude models the CAN diagnosis was associated with higher levels of circulating myo inositol (beta=0.23, p=2.9×10^-3^; **Supplementary Appendix 1 Section 3.1** and **Figure 3.4**) and ribonic acid (beta=0.27, p=2.2×10^-4^). These findings remained significant in after adjustment (p<0.05; **Supplementary Appendix 1 Section 3.2** and **Figure 3.4**), but lost significance after further adjustment for eGFR. (p>0.05; **Figure 2**; **Supplementary Appendix 1 Section 3.3** and **Figure 3.4**).

The E/I-ratio and the Valsalva maneuver were the components of the CAN diagnosis associated with the findings above. Higher values of both these CARTs were associated with lower levels of the myo inositol in crude models (E/I-ratio: beta=-0.22, p=0.002; Valsalva: beta=-0.21, p=0.029). Higher values of the Valsalva maneuver were also associated with lower levels of ribonic acid (beta=-0.20, p=0.04) and ribitol (beta=-0.23, p=0.017) in the crude model. The E/I ratio was inversely associated with ribonic acid in the crude (beta=-0.24, p=9.7×10^-4^) and adjusted models (beta=-0.21, p=0.04. No other associations were found.

#### Phenols

The CAN diagnosis was associated with higher levels of benzeneacetic acid (beta=0.21, p=8.2×10^-3^) and 4-hydroxybezeneacetic acid (beta=0.20, p=0.013) in the crude model. Benzeneacetic acid association with CAN remained significant for adjusted (beta=0.25, p=1.4×10^-3^) and fully adjusted model (beta=0.23, p=0.01; **Figure 2**; **Supplementary Appendix 1 Sections 3.2, 3.3** and **Figure 3.4**).

CARTs associated with phenols were the E/I ratio and the Vasalva maneuver, where higher values of these two CARTs were associated with lower levels of 4-hydroxybezeneacetic acid in the crude models (p<0.05; **Supplementary Appendix 1, Sections 5.2.1** and **5.4.1**). No other associations between CAN measures and other phenols were found.

#### Amino Acids

The CAN diagnosis was associated with two amino acids: methionine (crude model, beta=-0.16, p=0.049) and isoleucine (adjusted model, beta=-0.19, p=0.027). Participants with CAN had lower levels of these amino acids in crude and adjusted models. Associations were lost after further adjustment for eGFR (**Figure 2**, **Supplementary Appendix 1, Section 3.3**).

Higher resting heart rate was associated with lower methionine, only in crude models (beta=-0.20, p=0.04). No CARTs nor SDNN were associated with amino acids (**Supplementary Appendix 1**).

### Lipidomic Analysis

One-hundred-and-four lipid species from five major lipid classes were identified and underwent quality control. The 5 classes include diacyl-phosphatidylcholines (PCs), acyl-phosphatidylcholines, lyso-phosphatidylcholines (LPCs), triacylglycerols (TGs), free fatty acids (FFA) and sphingomyelins. The investigated lipids are listed in the online supplementary material (**Supplementary Appendix 2**).

#### Phosphatidylcholines

In crude models the CAN diagnosis was associated with lower levels of phosphatidylcholine 38:6 (beta=-0.20, p=0.048) and 32:0 (beta=-0.20, p=0.048), albeit just borderline significant. No other significant associations were found (**Supplementary Appendix 2**).

#### Triacylglycerols, Free Fatty Acids, Sphingomyelins

No significant associations were found between the CAN diagnosis or any measure of CAN and triacylglycerols, free fatty acids or sphingomyelins (**Supplementary Appendix 2**).

## Discussion

We investigated the serum metabolome in a cross-sectional study cohort of 302 persons with longstanding type 1 diabetes (median diabetes duration of 39 years) where CAN was present in 34% of participants. We found that the CAN diagnosis was associated with several metabolic pathways including hydroxy fatty acids, the TCA cycle, sugar derivatives, phenols, amino acids and phosphatidylcholines. However, after adjusting for eGFR the associations were restricted to hydroxy fatty acids, phenols and the TCA cycle metabolite citrate. Below we have discussed the possible implications of these findings in separate categories of outcomes.

### Hydroxy Fatty Acids

The CAN diagnosis was associated with higher levels of several hydroxy fatty acids even after full adjustment for eGFR. Results indicate that both parasympathetic and sympathetic dysfunction may drive these associations. Albeit changes in fatty acid metabolism affects cardio-renal complications by promoting inflammation and fibrosis (29), the specific role of hydroxy fatty acids has not been observed for other diabetes complications.

Associations between hydroxy fatty acids and the CAN diagnosis and indices of CAN have not been described previously. The pathophysiological mechanisms behind these associations remains to be explored.

### TCA-Cycle

In participants with CAN serum citrate levels were higher compared to participants without CAN through all levels of adjustments. This association could be driven predominately by parasympathetic dysfunction, as indicated by lower E/I ratio being associated with a higher level of citrate in crude and adjusted models, where the Valsalva maneuver only had the similar associations in crude models.

Our results coincide with previous findings in type 1 diabetes (22) where metabolites of the TCA cycle have been associated with measures of CAN. Here, autonomic dysfunction in the form of worse HRV indices (SDNN and RMSSD), was associated with higher levels of fumarate and citrate. The CAN diagnosis and individual CARTs were not found to associate with the other elements in the TCA cycle. Our findings confirm that CAN may be related to disruptions in the TCA cycle and in a manner where CAN measures recommended for diagnosing CAN (CARTs) are associated with these findings.

Citrate is an essential part of the TCA cycle which is the base of cellular energy metabolism. Studies in animal models have shown that decreased mitochondrial function is associated with microvascular diabetes complications (17). Diabetes animal nerve tissue studies have shown decreased levels of glycolytic and TCA intermediates in sural, sciatic, and dorsal root ganglion (30). Whether such changes are present in humans remains to be investigated. Taken together animal and human studies including the present study indicate that CAN is associated with changes in the TCA cycle. It remains however to be investigated how serum measures of TCA intermediates in CAN may be related in on a tissue specific level. In addition, the cross-sectional nature of our study does not allow for conclusions on causal relations.

### Sugar Derivatives

We found the CAN diagnosis and both adverse levels of parasympathetic and sympathetic indices of the CAN diagnosis to be associated with higher levels of myo inositol and ribonic acid. Such associations have not been presented in human studies of CAN previously. Myo inositol is a polyol and may play a part in the polyol or sorbitol pathway has been associated with diabetic complications through the production of reactive oxygen species. The polyol pathway has been associated with diabetic peripheral neuropathy (31) and nephropathy (32). Metabolic perturbations in the polyol pathway have been associated with reduced tissue myo inositol content and peripheral nerve conduction (33). Dietary myo inositol intake has been demonstrated to improve peripheral nerve function in a small study on diabetes individuals from the United Kingdom (34). Thus, the CAN diagnosis may be associated with a higher level of sugar derivates where some are associated with the polyol pathway. It is plausible that these sugar derivatives are risk factors to CAN and not vice versa.

### Phenols

We found that participants with CAN had higher levels of benzeneacetic acid and 4-hydroxybezeneacetic and that both parasympathetic and sympathetic dysfunction was associated with higher levels of these phenols. These associations have not been presented previously. It is not known how and if these phenols represent a significant role in pathogenic mechanisms leading to diabetes complications.

### Amino Acids

Participants with CAN had lower levels of methionine and isoleucine. In type 1 diabetes both increased and decreased levels of various amino acids have demonstrated when compared to healthy controls (35). Also, amino acid levels have been associated with renal function impairment in type 1 diabetes (27). However, the mechanisms and implications of these imbalances are not understood. Reduced (more pathological) levels of the HRV SDNN have been linked to lower levels of the amino acid glutamine in adjusted models (22). These findings underline that lower levels of some amino acids may be detrimental to the autonomic nervous system.

### Phosphatidylcholines

In crude analyses we found CAN to be associated with lower levels of phosphatidylcholine 38:6 and 32:0 and the E/I ratio the proportionally associated with phosphatidylcholine 34:3. Recently, opposite associations were found in persons with recent onset type 2 diabetes (36), where more pathological measures of autonomic dysfunction by HRV were associated with higher levels of several phosphatidylcholines including 32:0. The same study examined persons with recent onset type 1 diabetes and found no associations between CAN and lipid compounds. To our knowledge, no other studies have investigated association between CAN and lipidomic profiles. In concert, phosphatidylcholines may play a part in the development of CAN but by different mechanisms in short and long-term duration of diabetes. As phosphatidylcholines have been associated with decline in kidney function in type 1 diabetes (28, 37), changes in amino acids, polyol pathway, and free fatty acid metabolism may be a common denominator between CAN and diabetic kidney disease. Further studies looking into the metabolomic pathways and cross-talk between microvascular diabetic complications are suggested.

### Triacylglycerols, Free Fatty Acids, Sphingomyelins

Our study did not reveal associations between CAN and any other lipid markers than few phosphatidylcholines. Results from animal models indicate that diabetic neuropathy is associated with disturbances in the lipid metabolism (including triacylglycerols) in nervous tissues (38, 39). To our knowledge these findings have not been addressed in people with diabetes.

Autonomic dysfunction in itself could affect fat metabolism as adipose tissue is innervated by sympathetic nerve fibers (40). Also, hyperlipidemia seen as elevated triglycerides is a known risk factor for CAN (2). In concert it could be anticipated that more lipid metabolites were associated with CAN in this study. Some of the associations were lost during multiple testing correction suggesting limited power of the current study The reason for the paucity of associations could be that levels of these metabolites do not correlate with the levels in nervous tissue and that they may still have deleterious effects on nerves at a tissue level. Other reasons for the lacking associations may be that the metabolites are not associated with CAN or due to limited power of the study,

### Strengths and Limitations

The study is based on a large cohort of people with type 1 diabetes with CAN assessed by measures recommended for diagnosing CAN.

The cross-sectional nature of the study does not allow for conclusions made on causality. Our findings may be caused by residual confounding. Results may not be generalizable to a broad diabetes population as participants were not randomly recruited from the outpatient clinic. The findings of this study remain to be validated in other cohorts.

## Conclusion

Metabolic pathways, including the TCA cycle, hydroxy fatty acids, phosphatidylcholines and sugar derivatives, were associated with CAN in type 1 diabetes. These metabolic pathways could prove to be future modifiable risk factors for CAN possibly allowing for prevention of CAN. Validation studies of study results should be conducted in other similar cohorts and also in type 2 diabetes.

## Data Availability Statement

The datasets for this manuscript are not publicly available but may be requested by researchers who have the relevant legal permissions from the data protection agency. Requests to access the datasets should be directed to PR, peter.rossing@regionh.dk

## Ethics Statement

The studies involving human participants were reviewed and approved by the Local Ethical Committee of the capital region of Denmark. The patients/participants provided their written informed consent to participate in this study.

## Author Contributions

CH interpreted the data and drafted the manuscript. TS analyzed and interpreted the data and made critical revision of the manuscript for key intellectual content. TA conceived and designed the research, interpreted the data, made critical revision of the manuscript for key intellectual content, and supervised the study. IM, KT, LA, ST, and ML researched data and made critical revision of the manuscript for key intellectual content. TH interpreted the data and critically revised the manuscript for key intellectual content. PR conceived and designed the research, interpreted the data, handled funding and supervision, made critical revision of the manuscript for key intellectual content, and supervised the study. CH is the guarantor of this work and, as such, had full access to all the data in the study and takes responsibility for the integrity of the data and the accuracy of the data analysis. All authors contributed to the article and approved the submitted version.

## Funding

The work leading to this article received funding from the European Community’s Seventh Framework programme under grant agreement no. HEALTH-F2-2009-241544 (SysKID consortium). TA was supported by Novo Nordisk Foundation (Steno Collaborative Grant) NNF18OC0052457.

## Conflict of Interest

The authors declare that the research was conducted in the absence of any commercial or financial relationships that could be construed as a potential conflict of interest.

## Publisher’s Note

All claims expressed in this article are solely those of the authors and do not necessarily represent those of their affiliated organizations, or those of the publisher, the editors and the reviewers. Any product that may be evaluated in this article, or claim that may be made by its manufacturer, is not guaranteed or endorsed by the publisher.

## Abbreviations

CAN, Cardiovascular autonomic neuropathy; CARTs, Cardiovascular reflex tests; eGFR, Estimated glomerular filtration rate; HRV, Heart rate variability; SDNN, Standard deviation of normal-to-normal; T1DM, Type 1 diabetes; TCA, Tricarboxylic acid.
