# Supplementary material for: Cardiovascular Autonomic Neuropathy in Type 1 Diabetes Is Associated With Disturbances in TCA, Lipid, and Glucose Metabolism
Source: Front Endocrinol (Lausanne). 2022 Apr 14;13:831793. doi: 10.3389/fendo.2022.831793 (PMC9046722; doi:10.3389/fendo.2022.831793)
Supplement: Supplementary file 1 [file DataSheet_1.zip › Supplementary Appendix 1 .PDF]

0033_PROFIL_2017 Neuropathy Metabolomics

Tommi Suvitaival, tommi.raimo.leo.suvitaival@regionh.dk, Steno Diabetes Center Copenhagen

October 30, 2020

Contents

1 Settings **4**

2 Filter **5**

3 CAN Stat **6**

3.1 Crude Model 6

3.1.1 Tables of Model Coefficients 7

3.1.2 Forest Plot of Model Coefficients 9

3.2 Adjusted Model 10

3.2.1 Tables of Model Coefficients 11

3.2.2 Forest Plot of Model Coefficients 13

3.2.3 Bipartite Network of Model Coefficients 15

3.3 Fully Adjusted Model 17

3.3.1 Tables of Model Coefficients 18

3.3.2 Forest Plot of Model Coefficients 20

3.3.3 Bipartite Network of Model Coefficients 22

3.4 Combined Forest Plot 24

4 Vibration Sensation Threshold **25**

4.1 Crude Model 25

4.1.1 Tables of Model Coefficients 26

4.1.2 Forest Plot of Model Coefficients 28

4.2 Adjusted Model 29

4.2.1 Tables of Model Coefficients 30

4.2.2 Forest Plot of Model Coefficients 32

4.2.3 Bipartite Network of Model Coefficients 33

4.3 Fully Adjusted Model 35

4.3.1 Tables of Model Coefficients 36

4.3.2 Forest Plot of Model Coefficients . . . 38
4.3.3 Bipartite Network of Model Coefficients . . . 39

5 Secondary Analyses **41**

5.1 Resting HR Vagus . . . 41
5.1.1 Crude Model . . . 41
5.1.1.1 Tables of Model Coefficients . . . 42
5.1.1.2 Forest Plot of Model Coefficients . . . 44
5.1.2 Adjusted Model . . . 45
5.1.2.1 Tables of Model Coefficients . . . 46
5.1.2.2 Forest Plot of Model Coefficients . . . 48
5.1.3 Fully-Adjusted Model . . . 49
5.1.3.1 Tables of Model Coefficients . . . 50
5.1.3.2 Forest Plot of Model Coefficients . . . 52
5.2 Deep Breathing (E_I) . . . 53
5.2.1 Crude Model . . . 53
5.2.1.1 Tables of Model Coefficients . . . 54
5.2.1.2 Forest Plot of Model Coefficients . . . 56
5.2.2 Adjusted Model . . . 57
5.2.2.1 Tables of Model Coefficients . . . 58
5.2.2.2 Forest Plot of Model Coefficients . . . 60
5.2.3 Fully-Adjusted Model . . . 62
5.2.3.1 Tables of Model Coefficients . . . 63
5.2.3.2 Forest Plot of Model Coefficients . . . 65
5.3 Lying to Standing Test (lig_staa) . . . 66
5.3.1 Crude Model . . . 66
5.3.1.1 Tables of Model Coefficients . . . 67
5.3.1.2 Forest Plot of Model Coefficients . . . 69
5.3.2 Adjusted Model . . . 70
5.3.2.1 Tables of Model Coefficients . . . 71
5.3.2.2 Forest Plot of Model Coefficients . . . 73
5.3.3 Fully-Adjusted Model . . . 74
5.3.3.1 Tables of Model Coefficients . . . 75
5.3.3.2 Forest Plot of Model Coefficients . . . 77
5.4 Valsalva Maneuver (Valsal) . . . 78
5.4.1 Crude Model . . . 78
5.4.1.1 Tables of Model Coefficients . . . 79

5.4.1.2 Forest Plot of Model Coefficients 81
5.4.2 Adjusted Model 82
5.4.2.1 Tables of Model Coefficients 83
5.4.2.2 Forest Plot of Model Coefficients 85
5.4.3 Fully-Adjusted Model 86
5.4.3.1 Tables of Model Coefficients 87
5.4.3.2 Forest Plot of Model Coefficients 89
5.5 Heart Rate Variablity (SDNN) 90
5.5.1 Crude Model 90
5.5.1.1 Tables of Model Coefficients 91
5.5.1.2 Forest Plot of Model Coefficients 93
5.5.2 Adjusted Model 94
5.5.2.1 Tables of Model Coefficients 95
5.5.2.2 Forest Plot of Model Coefficients 97
5.5.3 Fully-Adjusted Model 98
5.5.3.1 Tables of Model Coefficients 99
5.5.3.2 Forest Plot of Model Coefficients 101
5.6 Neuropathy Questionnaire (mnsineuropat) 102
5.6.1 Crude Model 102
5.6.1.1 Tables of Model Coefficients 103
5.6.1.2 Forest Plot of Model Coefficients 104
5.7 Adjusted Model 105
5.7.0.1 Tables of Model Coefficients 106
5.7.0.2 Forest Plot of Model Coefficients 108
5.8 Fully-Adjusted Model 109
5.8.0.1 Tables of Model Coefficients 110
5.8.0.2 Forest Plot of Model Coefficients 112

6 Appendix **112**

1 Settings

2 Filter

3 CAN Stat

3.1 Crude Model

```
## [1] "Fitting models:"
## [1] "~ CAN_stat"
## [1] ""
```

3.1.1 Tables of Model Coefficients

```
## [1] ""
## [1] "Table: CAN_stat"
## [1] "  (from model: "
## [1] "   ~ CAN_stat)"
## [1] ""
##                                Name Coefficient  P.Value adj.P.Val
## 1  2,4-Dihydroxybutanoic acid; 28     0.34900 2.93e-08  2.20e-06
## 2  3,4-Dihydroxybutanoic acid; 27     0.33300 1.21e-07  4.53e-06
## 3                 Creatinine; 50     0.32000 3.86e-07  9.66e-06
## 4           Citric acid, 4TMS; 6     0.28100 8.11e-06  1.52e-04
## 5               Ribonic acid; 72     0.27200 1.53e-05  2.29e-04
## 6           Myo inositol 6TMS; 1     0.23200 2.34e-04  2.92e-03
## 7         Benzeneacetic acid; 47     0.21200 7.70e-04  8.25e-03
## 8  4-Hydroxybenzeneacetic acid; 4     0.20100 1.42e-03  1.33e-02
## 9         Glyceryl-glycoside; 59     0.19300 2.16e-03  1.71e-02
## 10      4-Deoxytetronic acid; 32     0.19200 2.28e-03  1.71e-02
## 11     Eicosapentaenoic acid; 55    -0.17800 4.81e-03  3.28e-02
## 12                  Glycerol; 58     0.17200 6.44e-03  4.02e-02
## 13      Docosahexaenoic acid; 53    -0.16600 8.48e-03  4.89e-02
## 14      4-Deoxytetronic acid; 33     0.16200 1.01e-02  4.92e-02
## 15          Methionine, 2TMS; 16    -0.16200 1.03e-02  4.92e-02
## 16             Octanoic acid; 68    -0.16100 1.05e-02  4.92e-02
## 17        Succinic acid, 2TMS; 7     0.15000 1.70e-02  7.13e-02
## 18                  Ribitol; 70     0.14900 1.83e-02  7.13e-02
## 19                  Ribitol; 71     0.14800 1.86e-02  7.13e-02
## 20    4-Hydroxybutanoic acid; 43     0.14800 1.90e-02  7.13e-02
## 21          Isoleucine, 2TMS; 18    -0.14600 2.02e-02  7.23e-02
## 22              Valine, 2TMS; 20    -0.13900 2.76e-02  9.42e-02
## 23              Serine, 3TMS; 14    -0.13300 3.53e-02  1.14e-01
## 24            Hydroxyproline; 64     0.13200 3.66e-02  1.14e-01
## 25 4-Hydroxyphenyllactic acid; 44     0.12500 4.65e-02  1.40e-01
## 26            Arachidic acid; 46    -0.11800 5.99e-02  1.73e-01
## 27         Fumaric acid, 2TMS; 9     0.11300 7.15e-02  1.99e-01
## 28             Glycine, 3TMS; 17     0.11200 7.56e-02  2.03e-01
## 29         Nonadecanoic acid; 66    -0.11000 8.09e-02  2.09e-01
## 30               1-Dodecanol; 36     0.10800 8.49e-02  2.12e-01
## 31             Leucine, 2TMS; 19    -0.10600 9.36e-02  2.26e-01
## 32             Decanoic acid; 52    -0.10300 1.01e-01  2.29e-01
## 33           Arabinopyranose; 51     0.10300 1.02e-01  2.29e-01
## 34       3-Indoleacetic acid; 40     0.10200 1.04e-01  2.29e-01
## 35          Malic acid, 3TMS; 11     0.09980 1.13e-01  2.42e-01
## 36          Tridecanoic acid; 74     0.09850 1.18e-01  2.45e-01
## 37             Nonanoic acid; 67     0.08930 1.56e-01  3.16e-01
## 38 2-Hydroxybutyric acid, 2TMS; 2    -0.08410 1.82e-01  3.53e-01
## 39            L-5-Oxoproline; 63     0.08380 1.83e-01  3.53e-01
## 40          Stearic acid, TMS; 2    -0.07590 2.28e-01  4.25e-01
## 41         Pyroglutamic acid; 69     0.07480 2.35e-01  4.25e-01
## 42             Glyceric acid; 30    -0.07430 2.38e-01  4.25e-01
## 43            Tartronic acid; 73    -0.06920 2.71e-01  4.73e-01
## 44                  Tyrosine; 75    -0.06150 3.29e-01  5.38e-01
## 45             Alanine, 2TMS; 25     0.06150 3.29e-01  5.38e-01
## 46           Threonine, 3TMS; 12    -0.06130 3.30e-01  5.38e-01
```

```
## 47                 Glycerol; 57     0.05960 3.44e-01  5.44e-01
## 48              Lactic acid; 29     0.05860 3.52e-01  5.44e-01
## 49            1-Monopalmitin; 37     0.05820 3.55e-01  5.44e-01
## 50      Arachidonic acid, TMS; 24     0.05520 3.81e-01  5.63e-01
## 51           1,3-Propanediol; 34    -0.05500 3.83e-01  5.63e-01
## 52     3-Indolepropionic acid; 41    -0.05100 4.18e-01  6.03e-01
## 53         Glutamic acid, 3TMS; 8     0.04750 4.51e-01  6.38e-01
## 54 alpha-ketoglutaric acid, TMS M     0.04480 4.77e-01  6.63e-01
## 55              Pyruvic acid; 31     0.04120 5.13e-01  6.99e-01
## 56         11-Eicosenoic acid; 35    -0.04000 5.26e-01  7.01e-01
## 57          Palmitic acid, TMS; 5    -0.03860 5.40e-01  7.01e-01
## 58         Heptadecanoic acid; 61    -0.03840 5.42e-01  7.01e-01
## 59              Hydroxylamine; 62    -0.03520 5.76e-01  7.33e-01
## 60            Dodecanoic acid; 54    -0.03020 6.32e-01  7.79e-01
## 61           alpha-Tocopherol; 26    -0.03000 6.34e-01  7.79e-01
## 62        2-Palmitoylglycerol; 39    -0.02720 6.65e-01  8.02e-01
## 63              Proline, 2TMS; 21     0.02650 6.74e-01  8.02e-01
## 64        Phenylalanine, 2TMS; 13    -0.02410 7.02e-01  8.23e-01
## 65 3-Hydroxybutyric acid, 2TMS; 1    -0.02300 7.15e-01  8.24e-01
## 66           Cholesterol, TMS; 23    -0.01960 7.55e-01  8.55e-01
## 67                Bisphenol A; 48     0.01890 7.64e-01  8.55e-01
## 68          Aminomalonic acid; 45    -0.01320 8.34e-01  9.06e-01
## 69                Campesterol; 49     0.01160 8.54e-01  9.06e-01
## 70               Ethanolamine; 56    -0.01040 8.68e-01  9.06e-01
## 71           Myristoleic acid; 65    -0.01030 8.70e-01  9.06e-01
## 72         Heptadecanoic acid; 60    -0.01030 8.70e-01  9.06e-01
## 73              Oleic acid, TMS; 3     0.00788 9.00e-01  9.17e-01
## 74  2-hydroxy Isovaleric acid; 38     0.00753 9.05e-01  9.17e-01
## 75           Linoleic acid, TMS; 4     0.00255 9.68e-01  9.68e-01
```

3.1.2 Forest Plot of Model Coefficients

```
## Warning: Ignoring unknown aesthetics: x
```

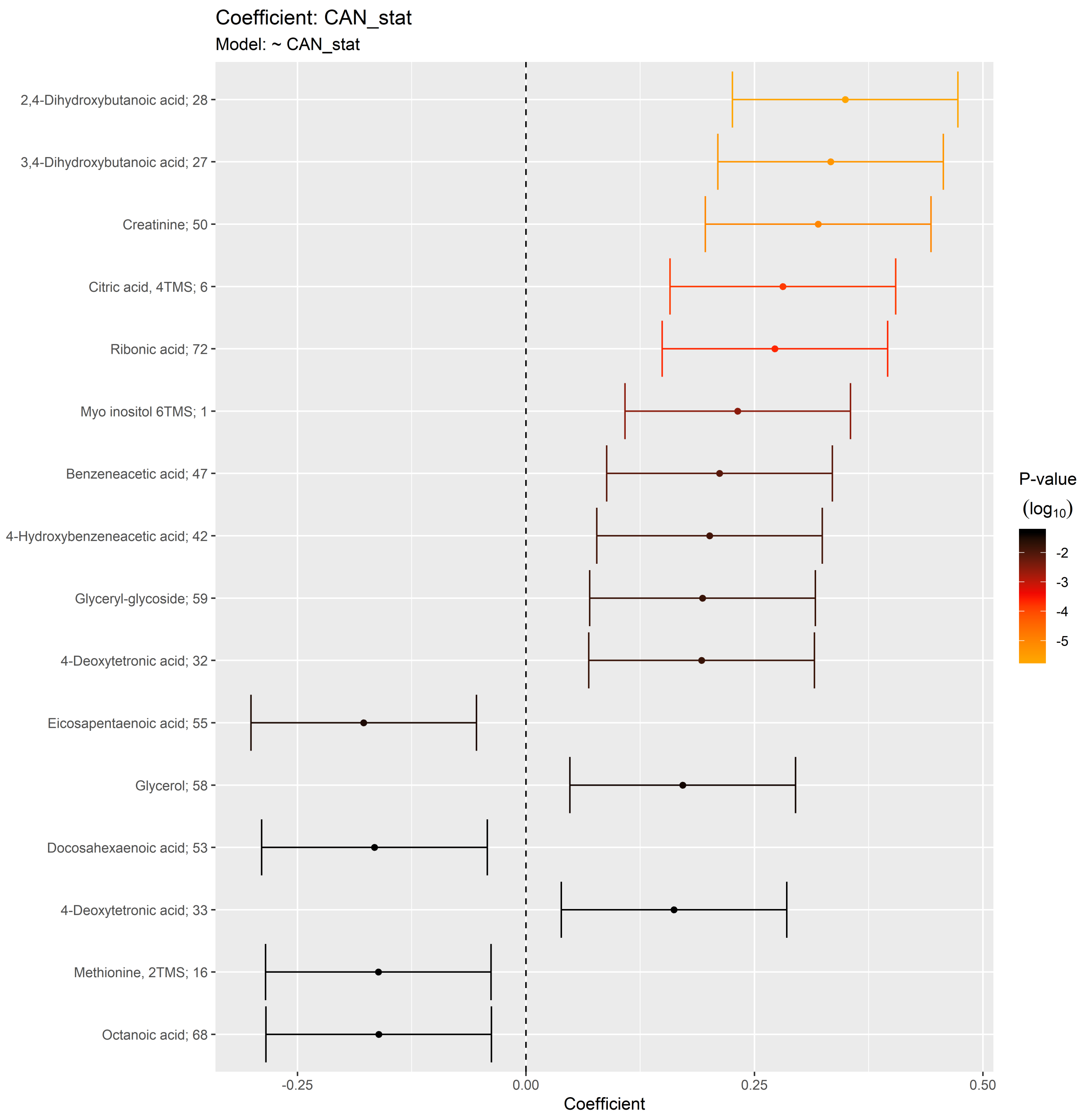

3.2 Adjusted Model

```
## [1] "Fitting models:"
## [1] "~ CAN_stat + Age + bmi + Blood_glucose + Duration_DM + Gender + Hba1c_baseline + log_Blood_TGA
## [1] ""
```

3.2.1 Tables of Model Coefficients

```
## [1] ""
## [1] "Table: CAN_stat"
## [1] "  (from model: "
## [1] "   ~ CAN_stat + Age + bmi + Blood_glucose + Duration_DM +"
## [1] "     Gender + Hba1c_baseline + log_Blood_TGA + Smoking + Statin +"
## [1] "     Total_cholesterol)"
## [1] ""
##                               Name Coefficient  P.Value adj.P.Val
## 1            Citric acid, 4TMS; 6    0.313000 1.82e-06  0.000101
## 2                  Creatinine; 50    0.307000 2.70e-06  0.000101
## 3  2,4-Dihydroxybutanoic acid; 28    0.297000 5.58e-06  0.000140
## 4  3,4-Dihydroxybutanoic acid; 27    0.261000 6.64e-05  0.001240
## 5         Benzeneacetic acid; 47    0.255000 9.84e-05  0.001480
## 6               Ribonic acid; 72    0.230000 4.46e-04  0.005570
## 7            Isoleucine, 2TMS; 18   -0.197000 2.62e-03  0.027600
## 8        4-Deoxytetronic acid; 32    0.195000 2.95e-03  0.027600
## 9            Myo inositol 6TMS; 1    0.189000 3.84e-03  0.032000
## 10     4-Hydroxybutanoic acid; 43    0.184000 5.00e-03  0.037500
## 11                   Glycerol; 58    0.177000 6.79e-03  0.046300
## 12         Succinic acid, 2TMS; 7    0.175000 7.59e-03  0.047400
## 13 4-Hydroxybenzeneacetic acid; 4    0.168000 1.05e-02  0.060500
## 14           Tridecanoic acid; 74    0.158000 1.61e-02  0.081800
## 15               Valine, 2TMS; 20   -0.157000 1.64e-02  0.081800
## 16              Leucine, 2TMS; 19   -0.147000 2.50e-02  0.109000
## 17              Glycine, 3TMS; 17    0.146000 2.58e-02  0.109000
## 18         Glyceryl-glycoside; 59    0.146000 2.62e-02  0.109000
## 19           Methionine, 2TMS; 16   -0.142000 3.02e-02  0.116000
## 20       Eicosapentaenoic acid; 55   -0.141000 3.10e-02  0.116000
## 21               Serine, 3TMS; 14   -0.137000 3.68e-02  0.128000
## 22              Nonanoic acid; 67    0.136000 3.78e-02  0.128000
## 23              Octanoic acid; 68   -0.135000 3.92e-02  0.128000
## 24             Hydroxyproline; 64    0.132000 4.42e-02  0.138000
## 25             L-5-Oxoproline; 63    0.127000 5.31e-02  0.159000
## 26 2-Hydroxybutyric acid, 2TMS; 2   -0.124000 5.86e-02  0.169000
## 27                    Ribitol; 70    0.121000 6.46e-02  0.176000
## 28 4-Hydroxyphenyllactic acid; 44    0.121000 6.56e-02  0.176000
## 29               Hydroxylamine; 62   -0.119000 6.95e-02  0.180000
## 30        Docosahexaenoic acid; 53   -0.117000 7.30e-02  0.183000
## 31                 1-Dodecanol; 36    0.113000 8.52e-02  0.206000
## 32       4-Deoxytetronic acid; 33    0.111000 9.03e-02  0.212000
## 33           Malic acid, 3TMS; 11    0.110000 9.43e-02  0.214000
## 34             Arachidic acid; 46   -0.108000 9.79e-02  0.216000
## 35          Fumaric acid, 2TMS; 9    0.092900 1.56e-01  0.334000
## 36        3-Indoleacetic acid; 40    0.091100 1.64e-01  0.342000
## 37             1-Monopalmitin; 37    0.079400 2.25e-01  0.449000
## 38          Pyroglutamic acid; 69    0.078100 2.33e-01  0.449000
## 39          Nonadecanoic acid; 66   -0.077200 2.38e-01  0.449000
## 40                    Ribitol; 71    0.077100 2.39e-01  0.449000
## 41          Aminomalonic acid; 45    0.068600 2.95e-01  0.540000
## 42           Stearic acid, TMS; 2   -0.067000 3.06e-01  0.547000
## 43              Decanoic acid; 52   -0.051400 4.32e-01  0.754000
## 44 3-Hydroxybutyric acid, 2TMS; 1   -0.049200 4.52e-01  0.770000
```

```
## 45                   Tyrosine; 75   -0.047600 4.68e-01  0.776000
## 46            Cholesterol, TMS; 23    0.046700 4.76e-01  0.776000
## 47          Palmitic acid, TMS; 5   -0.045000 4.92e-01  0.786000
## 48              1,3-Propanediol; 34   -0.041100 5.30e-01  0.818000
## 49      Arachidonic acid, TMS; 24    0.040700 5.35e-01  0.818000
## 50                Campesterol; 49   -0.038300 5.59e-01  0.835000
## 51             Arabinopyranose; 51    0.036100 5.82e-01  0.835000
## 52             Threonine, 3TMS; 12   -0.036100 5.82e-01  0.835000
## 53         2-Palmitoylglycerol; 39   -0.035300 5.90e-01  0.835000
## 54               Alanine, 2TMS; 25    0.031300 6.33e-01  0.839000
## 55                Bisphenol A; 48    0.028900 6.59e-01  0.839000
## 56          Heptadecanoic acid; 60    0.028700 6.61e-01  0.839000
## 57                Lactic acid; 29    0.028700 6.61e-01  0.839000
## 58               Pyruvic acid; 31    0.027800 6.71e-01  0.839000
## 59            alpha-Tocopherol; 26   -0.026500 6.86e-01  0.839000
## 60                   Glycerol; 57    0.026400 6.87e-01  0.839000
## 61             Tartronic acid; 73   -0.026000 6.92e-01  0.839000
## 62          11-Eicosenoic acid; 35   -0.025800 6.94e-01  0.839000
## 63             Dodecanoic acid; 54   -0.023300 7.22e-01  0.845000
## 64               Ethanolamine; 56   -0.022200 7.35e-01  0.845000
## 65              Glyceric acid; 30   -0.022200 7.35e-01  0.845000
## 66          Heptadecanoic acid; 61    0.021400 7.43e-01  0.845000
## 67 alpha-ketoglutaric acid, TMS M    0.020500 7.54e-01  0.845000
## 68  2-hydroxy Isovaleric acid; 38    0.019400 7.67e-01  0.846000
## 69          Glutamic acid, 3TMS; 8    0.017800 7.86e-01  0.854000
## 70           Linoleic acid, TMS; 4    0.009920 8.80e-01  0.939000
## 71              Oleic acid, TMS; 3    0.008350 8.99e-01  0.939000
## 72            Myristoleic acid; 65    0.008090 9.02e-01  0.939000
## 73              Proline, 2TMS; 21    0.006730 9.18e-01  0.943000
## 74     3-Indolepropionic acid; 41    0.002080 9.75e-01  0.988000
## 75         Phenylalanine, 2TMS; 13   -0.000666 9.92e-01  0.992000
```

3.2.2 Forest Plot of Model Coefficients

```
## Warning: Ignoring unknown aesthetics: x

## Warning: Ignoring unknown aesthetics: x

## Warning: Ignoring unknown aesthetics: x

## Warning: Ignoring unknown aesthetics: x

## Warning: Ignoring unknown aesthetics: x

## Warning: Ignoring unknown aesthetics: x

## Warning: Ignoring unknown aesthetics: x
```

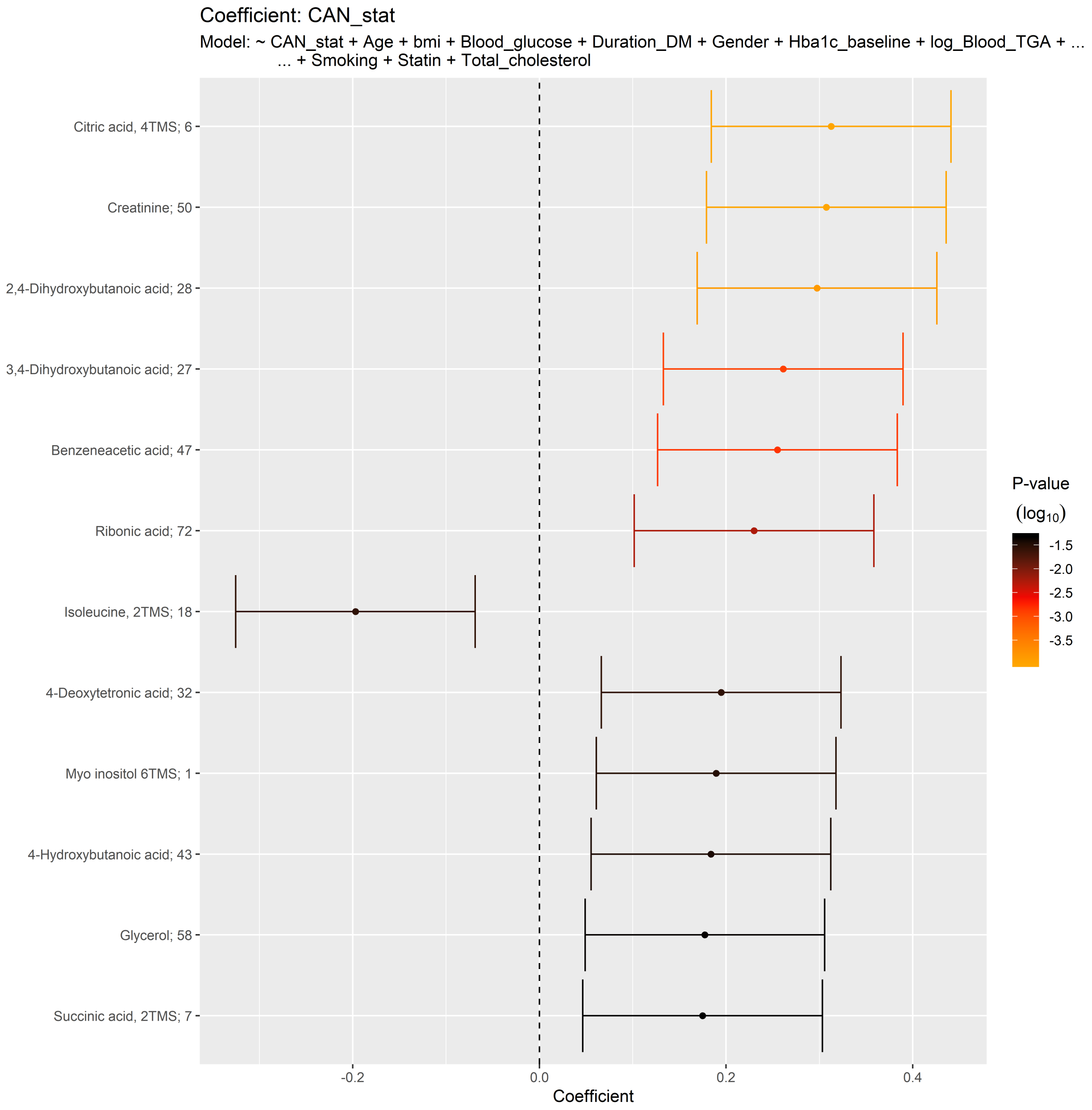

Coefficient: CAN_stat
Model: ~ CAN_stat + Age + bmi + Blood_glucose + Duration_DM + Gender + Hba1c_baseline + log_Blood_TGA + ...
... + Smoking + Statin + Total_cholesterol
Citric acid, 4TMS; 6
Creatinine; 50
2,4-Dihydroxybutanoic acid; 28
3,4-Dihydroxybutanoic acid; 27
Benzeneacetic acid; 47
Ribonic acid; 72
Isoleucine, 2TMS; 18
4-Deoxytetronic acid; 32
Myo inositol 6TMS; 1
4-Hydroxybutanoic acid; 43
Glycerol; 58
Succinic acid, 2TMS; 7
-0.2
0.0
0.2
0.4
Coefficient
P-value
($\log_{10}$)
-1.5
-2.0
-2.5
-3.0
-3.5

3.2.3 Bipartite Network of Model Coefficients

```
## [1] "bipartite_network_from_limma was created by Tommi Suvitaival"
## [1] "tommi.raimo.leo.suvitaival@regionh.dk"
## [1] "2019-05-06"

## Warning in if (drop.variables != "none") {: the condition has length > 1 and
## only the first element will be used

## Warning: Removed 4 rows containing missing values (geom_segment).

## Warning: Removed 1 rows containing missing values (geom_text).
```

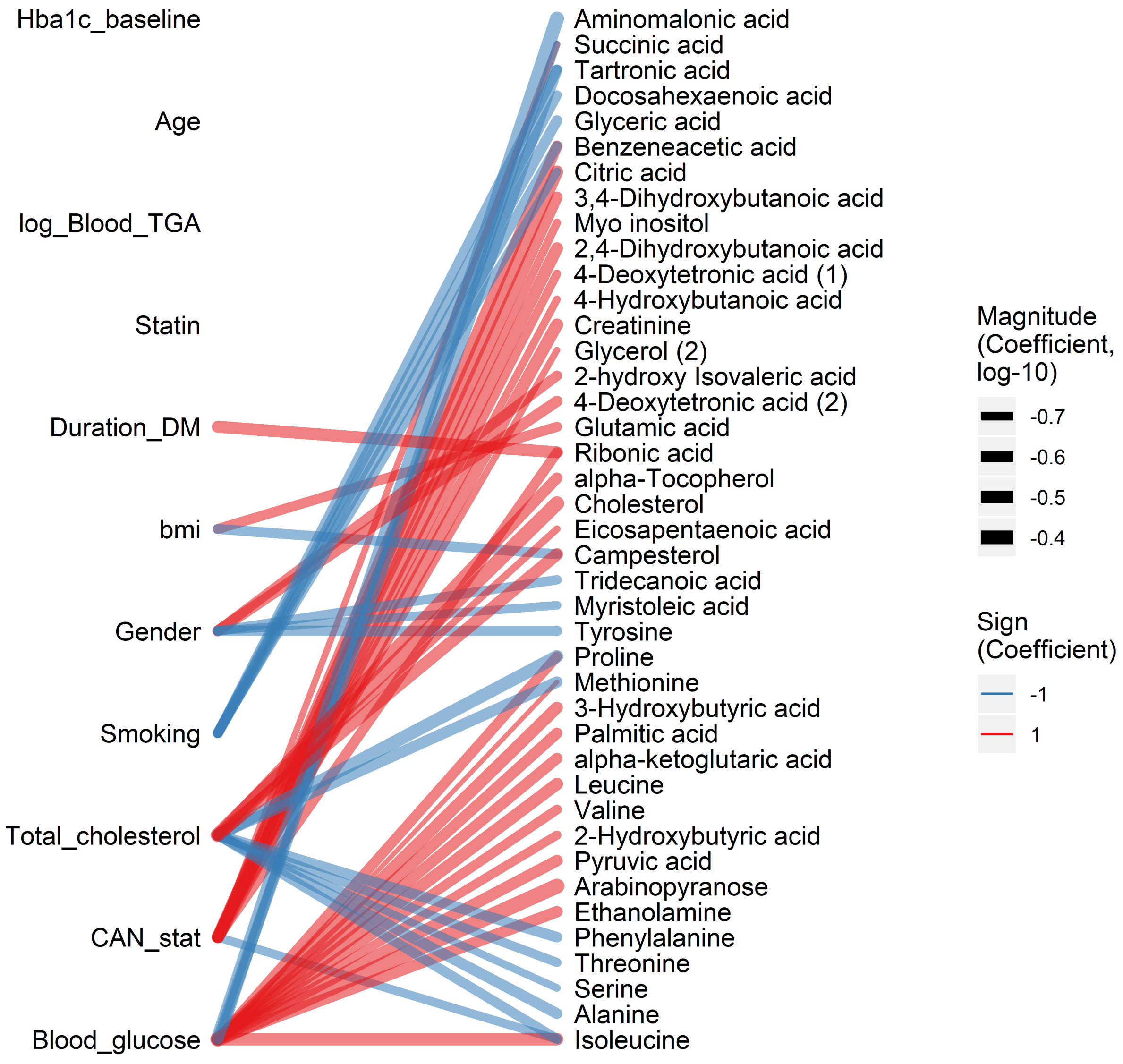

```
## [1] "bipartite_network_from_limma was created by Tommi Suvitaival"
## [1] "tommi.raimo.leo.suvitaival@regionh.dk"
## [1] "2019-05-06"

## Warning in if (drop.variables != "none") {: the condition has length > 1 and
## only the first element will be used

## Warning: Removed 4 rows containing missing values (geom_segment).

## Warning: Removed 1 rows containing missing values (geom_text).
```

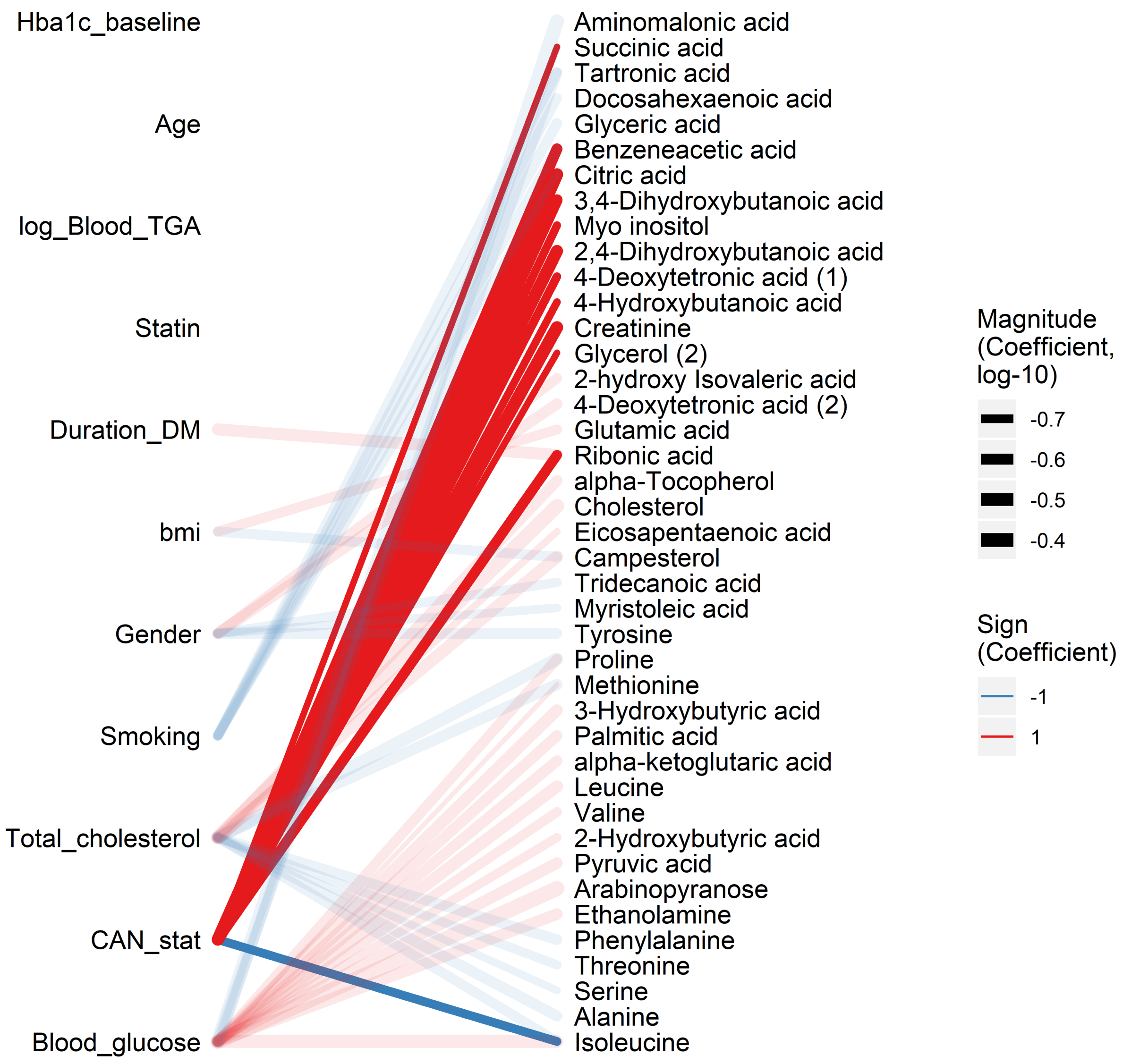

3.3 Fully Adjusted Model

```
## [1] "Fitting models:"
## [1] "~ CAN_stat + Age + bmi + Blood_glucose + Duration_DM + Gender + Hba1c_baseline + log_Blood_TGA +
## [1] ""
```

3.3.1 Tables of Model Coefficients

```
## [1] ""
## [1] "Table: CAN_stat"
## [1] "  (from model: "
## [1] "   ~ CAN_stat + Age + bmi + Blood_glucose + Duration_DM +"
## [1] "     Gender + Hba1c_baseline + log_Blood_TGA + Smoking + Statin +"
## [1] "     Total_cholesterol + egfr)"
## [1] ""
##                               Name Coefficient  P.Value adj.P.Val
## 1            Citric acid, 4TMS; 6     0.25800 0.000137    0.0103
## 2          Benzeneacetic acid; 47     0.23900 0.000425    0.0159
## 3                  Creatinine; 50     0.20900 0.002030    0.0508
## 4      4-Hydroxybutanoic acid; 43     0.18500 0.006790    0.1040
## 5          Succinic acid, 2TMS; 7     0.18400 0.006960    0.1040
## 6  2,4-Dihydroxybutanoic acid; 28     0.16400 0.014500    0.1820
## 7             Tridecanoic acid; 74     0.15900 0.019800    0.1990
## 8                Hydroxylamine; 62    -0.15700 0.021200    0.1990
## 9               L-5-Oxoproline; 63     0.14800 0.030400    0.2520
## 10 3,4-Dihydroxybutanoic acid; 27     0.14300 0.033600    0.2520
## 11                    Glycerol; 58     0.14000 0.039300    0.2680
## 12              Nonanoic acid; 67     0.13200 0.052700    0.3300
## 13      Eicosapentaenoic acid; 55    -0.12400 0.067500    0.3900
## 14           Isoleucine, 2TMS; 18    -0.11900 0.078000    0.3930
## 15                1-Dodecanol; 36     0.12000 0.078700    0.3930
## 16               Ribonic acid; 72     0.11400 0.091400    0.4280
## 17              Glycine, 3TMS; 17     0.10200 0.133000    0.5160
## 18       Docosahexaenoic acid; 53    -0.10100 0.135000    0.5160
## 19       4-Deoxytetronic acid; 32     0.10000 0.140000    0.5160
## 20             Arachidic acid; 46    -0.09970 0.144000    0.5160
## 21              Leucine, 2TMS; 19    -0.09900 0.145000    0.5160
## 22                    Ribitol; 70     0.09590 0.160000    0.5450
## 23          Cholesterol, TMS; 23     0.08970 0.183000    0.5980
## 24               Valine, 2TMS; 20    -0.08820 0.193000    0.6030
## 25              Octanoic acid; 68    -0.08510 0.211000    0.6180
## 26      Arachidonic acid, TMS; 24     0.08280 0.225000    0.6180
## 27  2-hydroxy Isovaleric acid; 38     0.08180 0.229000    0.6180
## 28             1-Monopalmitin; 37     0.08180 0.231000    0.6180
## 29          Methionine, 2TMS; 16    -0.07950 0.241000    0.6240
## 30                   Glycerol; 57     0.07640 0.263000    0.6560
## 31                    Ribitol; 71    -0.07400 0.272000    0.6580
## 32         Glyceryl-glycoside; 59     0.07130 0.294000    0.6890
## 33           Aminomalonic acid; 45     0.06830 0.313000    0.6950
## 34          Malic acid, 3TMS; 11     0.06810 0.317000    0.6950
## 35              Serine, 3TMS; 14    -0.06700 0.324000    0.6950
## 36                Lactic acid; 29     0.06600 0.334000    0.6950
## 37          Fumaric acid, 2TMS; 9     0.06180 0.364000    0.7260
## 38 4-Hydroxyphenyllactic acid; 44     0.06120 0.369000    0.7260
## 39         Glutamic acid, 3TMS; 8     0.05980 0.377000    0.7260
## 40             Hydroxyproline; 64     0.05500 0.419000    0.7670
## 41 3-Hydroxybutyric acid, 2TMS; 1    -0.05490 0.419000    0.7670
## 42          Nonadecanoic acid; 66    -0.05220 0.444000    0.7930
## 43 4-Hydroxybenzeneacetic acid; 4     0.05010 0.459000    0.8000
## 44              Decanoic acid; 52    -0.04630 0.496000    0.8450
```

```
## 45           Myo inositol 6TMS; 1     0.04100 0.541000    0.8790
## 46               Pyruvic acid; 31     0.04100 0.547000    0.8790
## 47         Heptadecanoic acid; 61     0.03940 0.563000    0.8790
## 48            1,3-Propanediol; 34    -0.03890 0.569000    0.8790
## 49                Bisphenol A; 48     0.03840 0.575000    0.8790
## 50        2-Palmitoylglycerol; 39    -0.03570 0.602000    0.9030
## 51                Campesterol; 49    -0.03220 0.635000    0.9080
## 52              Alanine, 2TMS; 25     0.02760 0.686000    0.9080
## 53            Arabinopyranose; 51     0.02570 0.703000    0.9080
## 54 alpha-ketoglutaric acid, TMS M     0.02590 0.704000    0.9080
## 55         11-Eicosenoic acid; 35    -0.02540 0.710000    0.9080
## 56          Linoleic acid, TMS; 4     0.02530 0.710000    0.9080
## 57              Glyceric acid; 30     0.02490 0.713000    0.9080
## 58 2-Hydroxybutyric acid, 2TMS; 2    -0.02440 0.718000    0.9080
## 59            Oleic acid, TMS; 3     0.02450 0.719000    0.9080
## 60         Heptadecanoic acid; 60     0.02340 0.731000    0.9080
## 61            Tartronic acid; 73     -0.02260 0.739000    0.9080
## 62           alpha-Tocopherol; 26    -0.01900 0.780000    0.9410
## 63               Ethanolamine; 56     0.01800 0.791000    0.9410
## 64           Stearic acid, TMS; 2    -0.01660 0.808000    0.9420
## 65          Pyroglutamic acid; 69     0.01580 0.817000    0.9420
## 66       4-Deoxytetronic acid; 33     0.01380 0.838000    0.9490
## 67                   Tyrosine; 75     0.01300 0.848000    0.9490
## 68     3-Indolepropionic acid; 41     0.00961 0.888000    0.9690
## 69         Palmitic acid, TMS; 5     -0.00917 0.893000    0.9690
## 70        3-Indoleacetic acid; 40     0.00802 0.906000    0.9690
## 71            Threonine, 3TMS; 12    -0.00589 0.931000    0.9690
## 72              Proline, 2TMS; 21    -0.00441 0.948000    0.9690
## 73            Dodecanoic acid; 54     0.00370 0.957000    0.9690
## 74        Phenylalanine, 2TMS; 13    -0.00281 0.967000    0.9690
## 75           Myristoleic acid; 65     0.00268 0.969000    0.9690
```

3.3.2 Forest Plot of Model Coefficients

```
## Warning: Ignoring unknown aesthetics: x
## Warning: Ignoring unknown aesthetics: x
## Warning: Ignoring unknown aesthetics: x
## Warning: Ignoring unknown aesthetics: x
## Warning: Ignoring unknown aesthetics: x
## Warning: Ignoring unknown aesthetics: x
## Warning: Ignoring unknown aesthetics: x
```

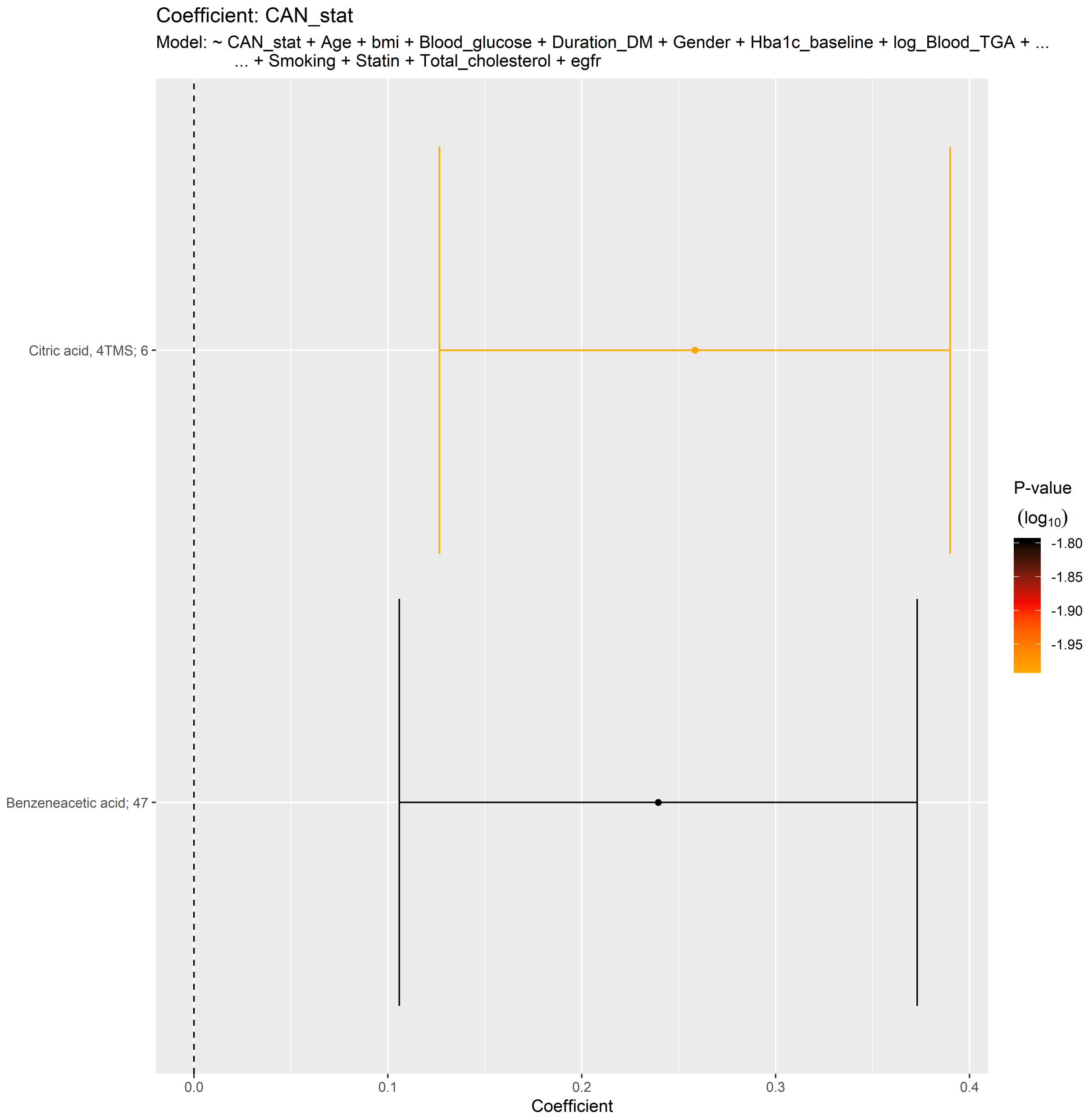
Coefficient: CAN_stat
Model: ~ CAN_stat + Age + bmi + Blood_glucose + Duration_DM + Gender + Hba1c_baseline + log_Blood_TGA + ...
... + Smoking + Statin + Total_cholesterol + egfr
Citric acid, 4TMS; 6
Benzeneacetic acid; 47
0.0
0.1
0.2
0.3
0.4
Coefficient
P-value
(log10)
-1.80
-1.85
-1.90
-1.95

3.3.3 Bipartite Network of Model Coefficients

```
## [1] "bipartite_network_from_limma was created by Tommi Suvitaival"
## [1] "tommi.raimo.leo.suvitaival@regionh.dk"
## [1] "2019-05-06"

## Warning in if (drop.variables != "none") {: the condition has length > 1 and
## only the first element will be used

## Warning: Removed 5 rows containing missing values (geom_segment).

## Warning: Removed 1 rows containing missing values (geom_text).
```

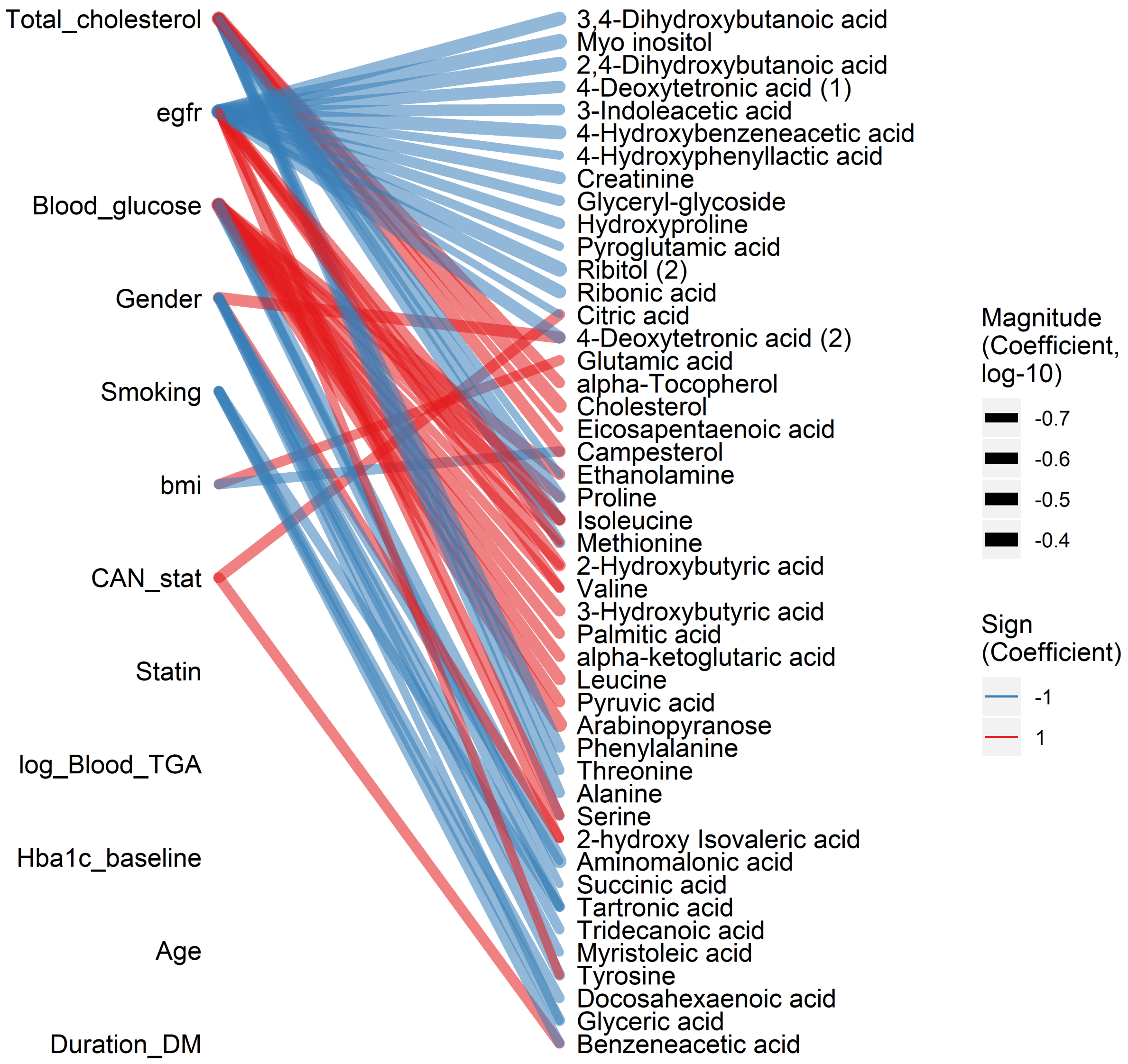

```
## [1] "bipartite_network_from_limma was created by Tommi Suvitaival"
## [1] "tommi.raimo.leo.suvitaival@regionh.dk"
## [1] "2019-05-06"

## Warning in if (drop.variables != "none") {: the condition has length > 1 and
## only the first element will be used

## Warning: Removed 5 rows containing missing values (geom_segment).

## Warning: Removed 1 rows containing missing values (geom_text).
```

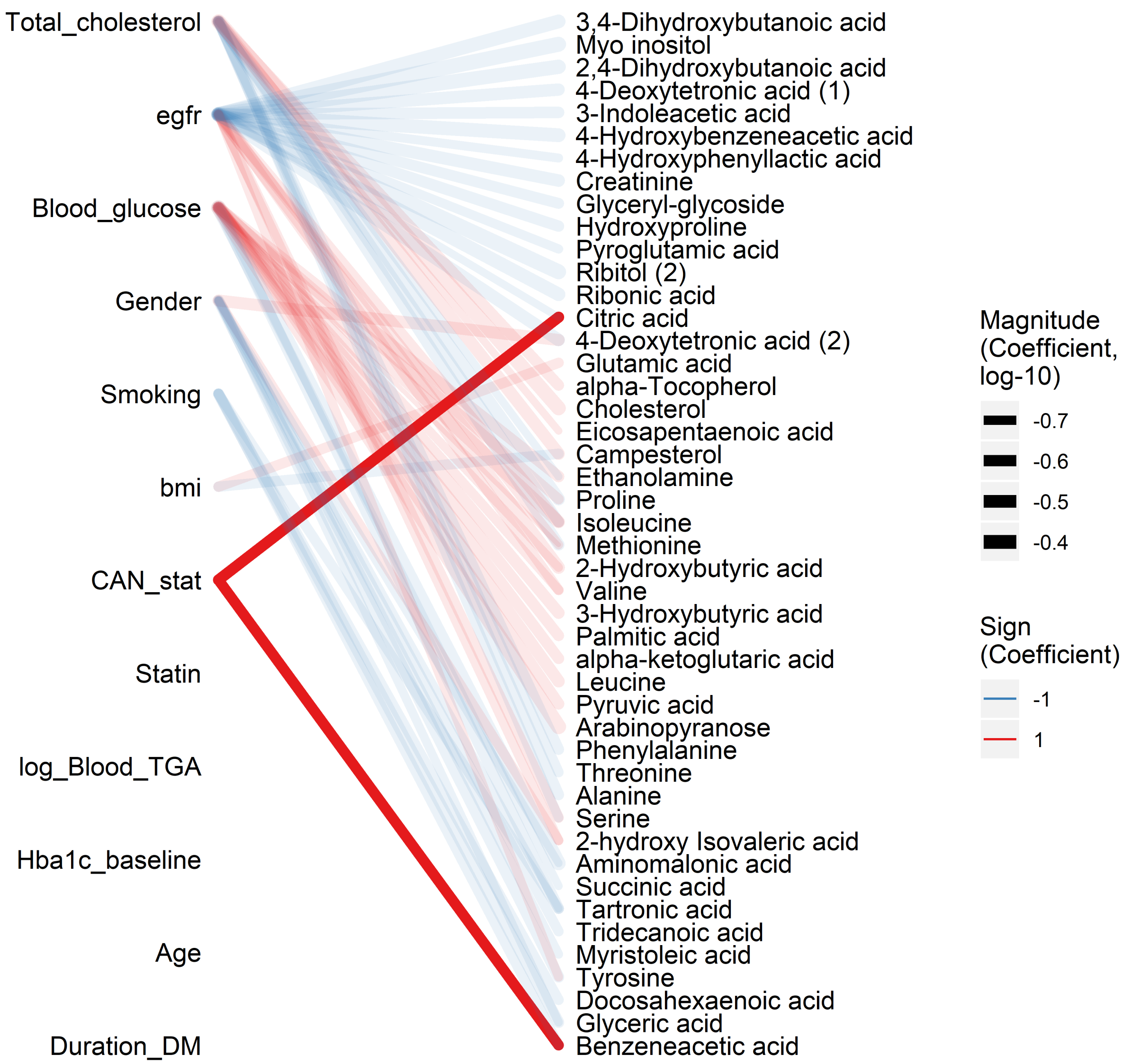

3.4 Combined Forest Plot

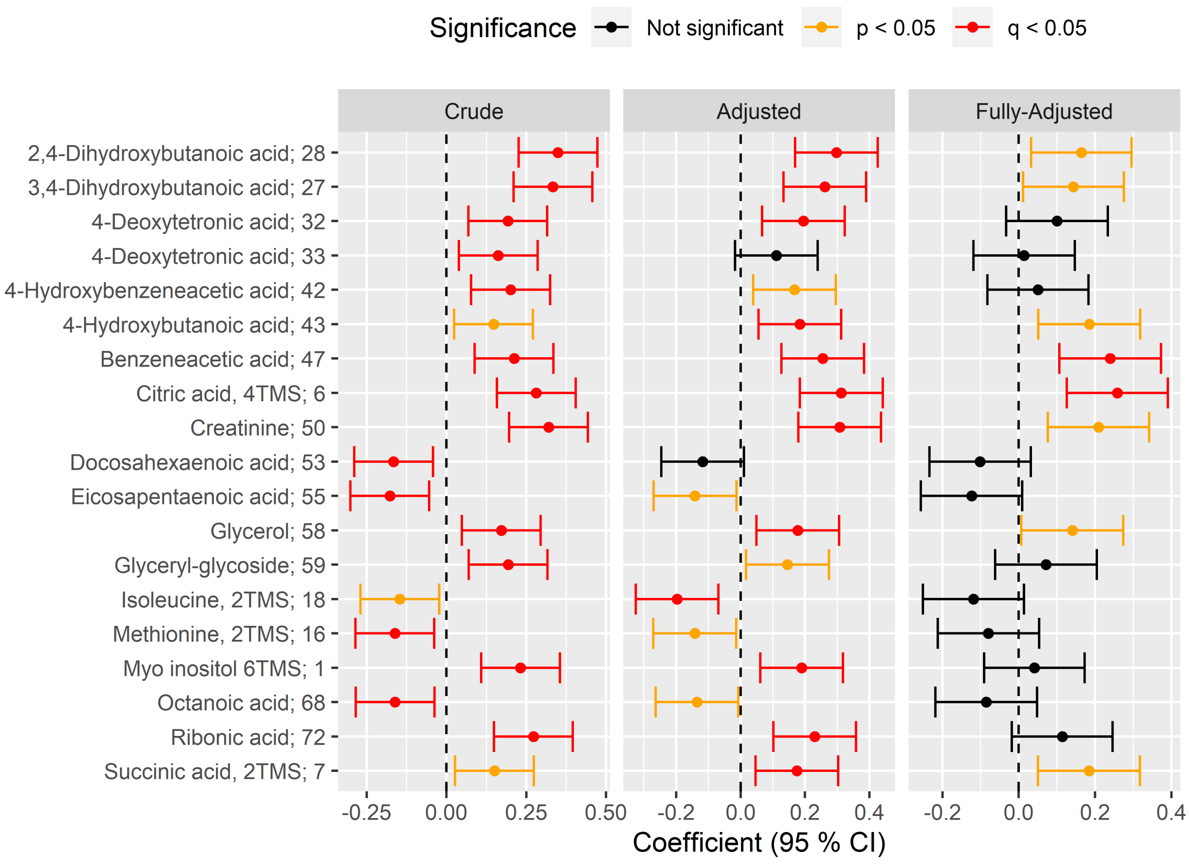

4 Vibration Sensation Threshold

4.1 Crude Model

```
## [1] "Fitting models:"
## [1] "~ Vib_pat"
## [1] ""
```

4.1.1 Tables of Model Coefficients

```
## [1] ""
## [1] "Table: Vib_pat"
## [1] "  (from model: "
## [1] "   ~ Vib_pat)"
## [1] ""
##                                Name Coefficient  P.Value adj.P.Val
## 1                       Ribitol; 71     0.23400 6.47e-05   0.00485
## 2  4-Hydroxyphenyllactic acid; 44     0.18700 1.43e-03   0.05360
## 3  3,4-Dihydroxybutanoic acid; 27     0.17100 3.54e-03   0.08840
## 4  2,4-Dihydroxybutanoic acid; 28     0.16400 5.19e-03   0.09720
## 5            Myo inositol 6TMS; 1     0.14700 1.21e-02   0.18200
## 6                      Ribitol; 70     0.13800 1.85e-02   0.23100
## 7  2-Hydroxybutyric acid, 2TMS; 2    -0.13400 2.21e-02   0.23700
## 8  4-Hydroxybenzeneacetic acid; 4     0.12700 3.04e-02   0.24400
## 9            alpha-Tocopherol; 26     0.12500 3.20e-02   0.24400
## 10               Ethanolamine; 56    -0.12500 3.25e-02   0.24400
## 11               L-5-Oxoproline; 63     0.12200 3.65e-02   0.24900
## 12            1,3-Propanediol; 34    -0.12000 3.98e-02   0.24900
## 13         Glyceryl-glycoside; 59     0.11400 5.08e-02   0.29300
## 14                 Creatinine; 50     0.11200 5.64e-02   0.29300
## 15         Nonadecanoic acid; 66    -0.11000 5.94e-02   0.29300
## 16              Hydroxyproline; 64     0.10700 6.62e-02   0.29300
## 17         Fumaric acid, 2TMS; 9     0.10700 6.64e-02   0.29300
## 18      4-Deoxytetronic acid; 32     0.10500 7.21e-02   0.30000
## 19               Lactic acid; 29    -0.10100 8.54e-02   0.33000
## 20        Heptadecanoic acid; 60    -0.09980 8.79e-02   0.33000
## 21                  Glycerol; 58     0.09720 9.66e-02   0.33300
## 22     Arachidonic acid, TMS; 24    -0.09430 1.07e-01   0.33300
## 23     Eicosapentaenoic acid; 55     0.09330 1.11e-01   0.33300
## 24          Aminomalonic acid; 45    -0.09290 1.12e-01   0.33300
## 25             1-Monopalmitin; 37     0.09250 1.14e-01   0.33300
## 26               Ribonic acid; 72     0.09210 1.15e-01   0.33300
## 27      4-Deoxytetronic acid; 33     0.08350 1.54e-01   0.41900
## 28              Octanoic acid; 68    -0.08230 1.60e-01   0.41900
## 29          Methionine, 2TMS; 16     0.08080 1.67e-01   0.41900
## 30       2-Palmitoylglycerol; 39     0.08060 1.68e-01   0.41900
## 31      Docosahexaenoic acid; 53    -0.07970 1.73e-01   0.41900
## 32              Nonanoic acid; 67    -0.07380 2.07e-01   0.48500
## 33              Decanoic acid; 52     0.07090 2.26e-01   0.48500
## 34            Arachidic acid; 46    -0.07080 2.26e-01   0.48500
## 35            Tartronic acid; 73    -0.06850 2.42e-01   0.48500
## 36              Alanine, 2TMS; 25     0.06750 2.49e-01   0.48500
## 37         Cholesterol, TMS; 23    -0.06550 2.63e-01   0.48500
## 38          Stearic acid, TMS; 2    -0.06480 2.68e-01   0.48500
## 39        3-Indoleacetic acid; 40     0.06480 2.68e-01   0.48500
## 40          Citric acid, 4TMS; 6     0.06470 2.69e-01   0.48500
## 41            Arabinopyranose; 51     0.06440 2.71e-01   0.48500
## 42                  Tyrosine; 75     0.06420 2.72e-01   0.48500
## 43        Heptadecanoic acid; 61    -0.06350 2.78e-01   0.48500
## 44             Glycine, 3TMS; 17     0.06190 2.90e-01   0.49500
## 45               Bisphenol A; 48    -0.06050 3.01e-01   0.50100
## 46              Glyceric acid; 30    -0.05880 3.15e-01   0.51300
```

```
## 47           Threonine, 3TMS; 12     0.05510 3.46e-01   0.55300
## 48             Pyruvic acid; 31     0.05320 3.63e-01   0.56700
## 49        Benzeneacetic acid; 47     0.04830 4.09e-01   0.62600
## 50              Proline, 2TMS; 21     0.04510 4.41e-01   0.66100
## 51          Pyroglutamic acid; 69     0.04140 4.79e-01   0.70400
## 52                1-Dodecanol; 36    -0.03940 5.00e-01   0.72200
## 53    4-Hydroxybutanoic acid; 43     0.03560 5.43e-01   0.76500
## 54                  Glycerol; 57    -0.03490 5.51e-01   0.76500
## 55        11-Eicosenoic acid; 35     0.03240 5.80e-01   0.78500
## 56              Serine, 3TMS; 14    -0.03190 5.86e-01   0.78500
## 57           Dodecanoic acid; 54     0.02810 6.30e-01   0.83000
## 58          Linoleic acid, TMS; 4     0.02600 6.57e-01   0.84900
## 59  2-hydroxy Isovaleric acid; 38    -0.02160 7.12e-01   0.90100
## 60           Myristoleic acid; 65     0.02090 7.21e-01   0.90100
## 61 alpha-ketoglutaric acid, TMS M     0.01900 7.45e-01   0.91600
## 62              Hydroxylamine; 62    -0.01620 7.82e-01   0.94600
## 63            Oleic acid, TMS; 3     0.01260 8.29e-01   0.96900
## 64               Campesterol; 49     0.01230 8.34e-01   0.96900
## 65           Tridecanoic acid; 74    -0.01180 8.40e-01   0.96900
## 66           Malic acid, 3TMS; 11     0.00984 8.67e-01   0.97200
## 67         Succinic acid, 2TMS; 7     0.00970 8.68e-01   0.97200
## 68              Valine, 2TMS; 20     0.00762 8.96e-01   0.97200
## 69             Leucine, 2TMS; 19    -0.00682 9.07e-01   0.97200
## 70 3-Hydroxybutyric acid, 2TMS; 1    -0.00633 9.14e-01   0.97200
## 71        Phenylalanine, 2TMS; 13     0.00375 9.49e-01   0.97200
## 72          Isoleucine, 2TMS; 18    -0.00335 9.54e-01   0.97200
## 73        Glutamic acid, 3TMS; 8     0.00330 9.55e-01   0.97200
## 74    3-Indolepropionic acid; 41     0.00299 9.59e-01   0.97200
## 75          Palmitic acid, TMS; 5    -0.00130 9.82e-01   0.98200
```

4.1.2 Forest Plot of Model Coefficients

```
## Warning: Ignoring unknown aesthetics: x
```

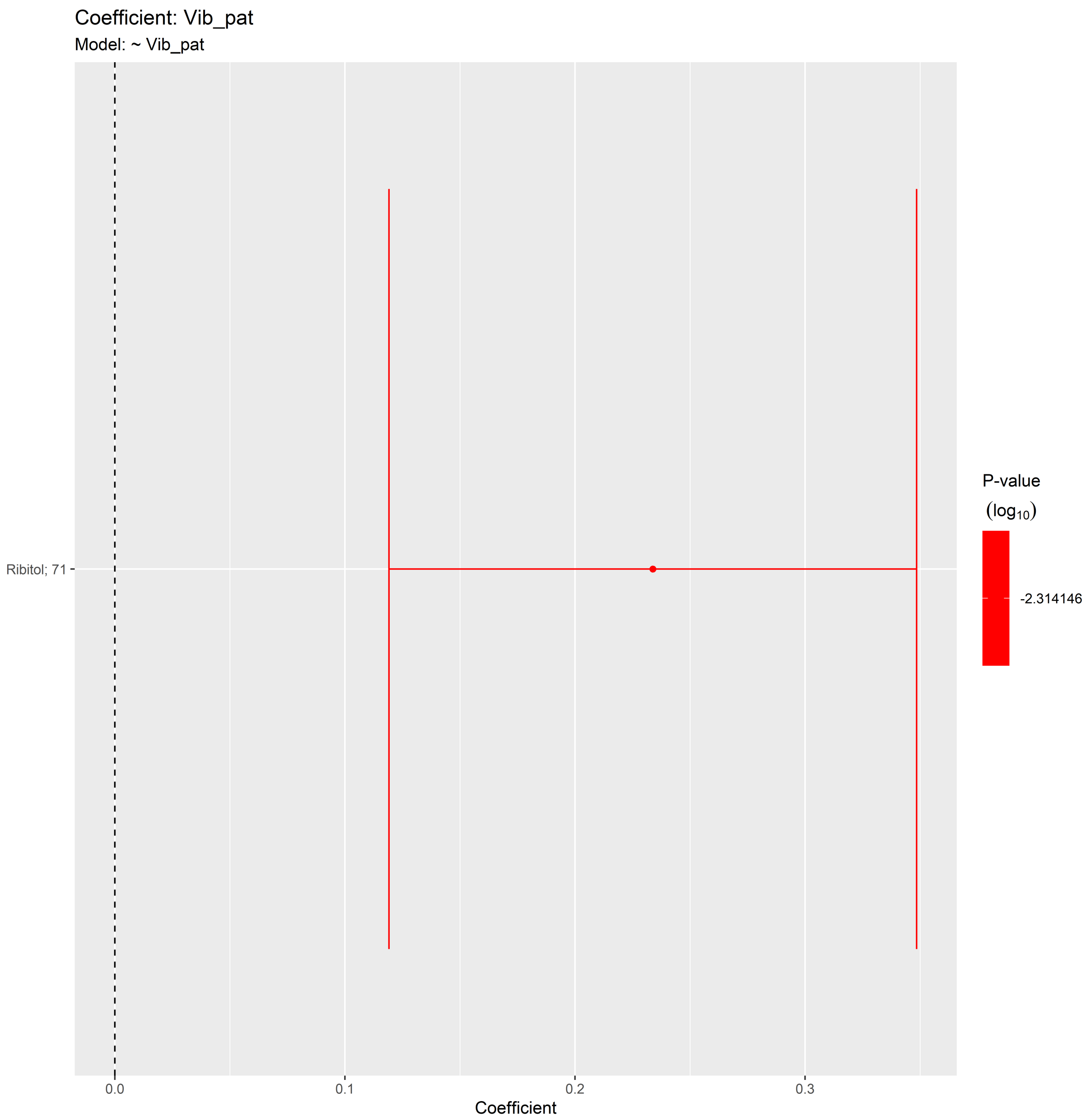

4.2 Adjusted Model

```
## [1] "Fitting models:"
## [1] "~ Vib_pat + Age + bmi + Blood_glucose + Duration_DM + Gender + Hba1c_baseline + log_Blood_TGA +
## [1] ""
```

4.2.1 Tables of Model Coefficients

```
## [1] ""
## [1] "Table: Vib_pat"
## [1] "  (from model: "
## [1] "   ~ Vib_pat + Age + bmi + Blood_glucose + Duration_DM +"
## [1] "     Gender + Hba1c_baseline + log_Blood_TGA + Smoking + Statin +"
## [1] "     Total_cholesterol)"
## [1] ""
##                               Name Coefficient P.Value adj.P.Val
## 1  2-Hydroxybutyric acid, 2TMS; 2    -0.19100 0.00377     0.225
## 2       Arachidonic acid, TMS; 24    -0.17500 0.00764     0.225
## 3                     Ribitol; 71     0.17200 0.00899     0.225
## 4  4-Hydroxyphenyllactic acid; 44     0.15300 0.02030     0.323
## 5        Docosahexaenoic acid; 53    -0.15100 0.02150     0.323
## 6              Hydroxyproline; 64     0.14000 0.03370     0.415
## 7              Arachidic acid; 46    -0.13300 0.04270     0.415
## 8                Ethanolamine; 56    -0.13200 0.04440     0.415
## 9          Nonadecanoic acid; 66    -0.12800 0.05160     0.415
## 10          Stearic acid, TMS; 2    -0.12600 0.05530     0.415
## 11        Heptadecanoic acid; 60    -0.11900 0.06950     0.454
## 12               Lactic acid; 29    -0.11800 0.07390     0.454
## 13           1,3-Propanediol; 34    -0.11500 0.07910     0.454
## 14            Threonine, 3TMS; 12     0.11300 0.08480     0.454
## 15             Octanoic acid; 68    -0.10900 0.09640     0.482
## 16 2,4-Dihydroxybutanoic acid; 28     0.10400 0.11500     0.539
## 17        Heptadecanoic acid; 61    -0.09730 0.13900     0.613
## 18          Myo inositol 6TMS; 1     0.09180 0.16300     0.639
## 19 3-Hydroxybutyric acid, 2TMS; 1    -0.09000 0.17100     0.639
## 20      4-Deoxytetronic acid; 32     0.08950 0.17400     0.639
## 21                Creatinine; 50     0.08840 0.17900     0.639
## 22           L-5-Oxoproline; 63     0.08090 0.21800     0.651
## 23 alpha-ketoglutaric acid, TMS M    -0.07940 0.22700     0.651
## 24          Aminomalonic acid; 45    -0.07830 0.23400     0.651
## 25        Glyceryl-glycoside; 59     0.07750 0.23800     0.651
## 26                   Ribitol; 70     0.07750 0.23800     0.651
## 27          Isoleucine, 2TMS; 18    -0.07590 0.24800     0.651
## 28                  Glycerol; 58     0.07560 0.25000     0.651
## 29             Glycine, 3TMS; 17     0.07540 0.25200     0.651
## 30       Phenylalanine, 2TMS; 13     0.07030 0.28500     0.690
## 31  2-hydroxy Isovaleric acid; 38    -0.06710 0.30800     0.690
## 32 3,4-Dihydroxybutanoic acid; 27     0.06690 0.30900     0.690
## 33                  Tyrosine; 75     0.06630 0.31300     0.690
## 34          Methionine, 2TMS; 16     0.06570 0.31800     0.690
## 35          Linoleic acid, TMS; 4     0.06350 0.33400     0.690
## 36              Ribonic acid; 72     0.06310 0.33800     0.690
## 37     4-Hydroxybutanoic acid; 43     0.06220 0.34400     0.690
## 38    3-Indolepropionic acid; 41     0.06030 0.35900     0.690
## 39             Leucine, 2TMS; 19    -0.05820 0.37600     0.690
## 40              Pyruvic acid; 31    -0.05800 0.37700     0.690
## 41 4-Hydroxybenzeneacetic acid; 4     0.05710 0.38500     0.690
## 42             Decanoic acid; 52     0.05700 0.38600     0.690
## 43      4-Deoxytetronic acid; 33     0.05300 0.42000     0.703
## 44             Alanine, 2TMS; 25     0.05250 0.42400     0.703
```

```
## 45                 Bisphenol A; 48    -0.05240 0.42600     0.703
## 46             1-Monopalmitin; 37     0.05180 0.43100     0.703
## 47              Glyceric acid; 30    -0.04920 0.45500     0.716
## 48           Citric acid, 4TMS; 6     0.04880 0.45800     0.716
## 49           alpha-Tocopherol; 26     0.04530 0.49100     0.739
## 50                 1-Dodecanol; 36    -0.04510 0.49300     0.739
## 51            Arabinopyranose; 51     0.04280 0.51500     0.753
## 52          Palmitic acid, TMS; 5    -0.04210 0.52200     0.753
## 53        Glutamic acid, 3TMS; 8    -0.03860 0.55800     0.779
## 54          Fumaric acid, 2TMS; 9     0.03830 0.56100     0.779
## 55           Malic acid, 3TMS; 11    -0.03610 0.58300     0.787
## 56           Tridecanoic acid; 74     0.03560 0.58800     0.787
## 57              Nonanoic acid; 67    -0.03470 0.59800     0.787
## 58              Hydroxylamine; 62    -0.03140 0.63300     0.787
## 59                   Glycerol; 57     0.03130 0.63400     0.787
## 60           Cholesterol, TMS; 23    -0.03030 0.64500     0.787
## 61        2-Palmitoylglycerol; 39     0.02970 0.65200     0.787
## 62             Oleic acid, TMS; 3    -0.02950 0.65400     0.787
## 63             Tartronic acid; 73    -0.02880 0.66100     0.787
## 64           Myristoleic acid; 65     0.02750 0.67600     0.792
## 65         Benzeneacetic acid; 47     0.02250 0.73200     0.845
## 66               Valine, 2TMS; 20     0.02010 0.76000     0.863
## 67            Dodecanoic acid; 54     0.01790 0.78600     0.880
## 68          Pyroglutamic acid; 69    -0.01460 0.82400     0.904
## 69        Succinic acid, 2TMS; 7     0.01400 0.83200     0.904
## 70               Serine, 3TMS; 14     0.00880 0.89400     0.945
## 71                Campesterol; 49    -0.00810 0.90200     0.945
## 72        3-Indoleacetic acid; 40    -0.00767 0.90700     0.945
## 73         11-Eicosenoic acid; 35    -0.00562 0.93200     0.948
## 74     Eicosapentaenoic acid; 55    -0.00513 0.93800     0.948
## 75              Proline, 2TMS; 21     0.00427 0.94800     0.948
```

4.2.2 Forest Plot of Model Coefficients

```
## Warning: Ignoring unknown aesthetics: x

## Warning: Ignoring unknown aesthetics: x

## Warning: Ignoring unknown aesthetics: x

## Warning: Ignoring unknown aesthetics: x

## Warning: Ignoring unknown aesthetics: x

## Warning: Ignoring unknown aesthetics: x

## Warning: Ignoring unknown aesthetics: x

## NULL
```

4.2.3 Bipartite Network of Model Coefficients

```
## [1] "bipartite_network_from_limma was created by Tommi Suvitaival"
## [1] "tommi.raimo.leo.suvitaival@regionh.dk"
## [1] "2019-05-06"

## Warning in if (drop.variables != "none") {: the condition has length > 1 and
## only the first element will be used

## Warning: Removed 4 rows containing missing values (geom_segment).

## Warning: Removed 1 rows containing missing values (geom_text).
```

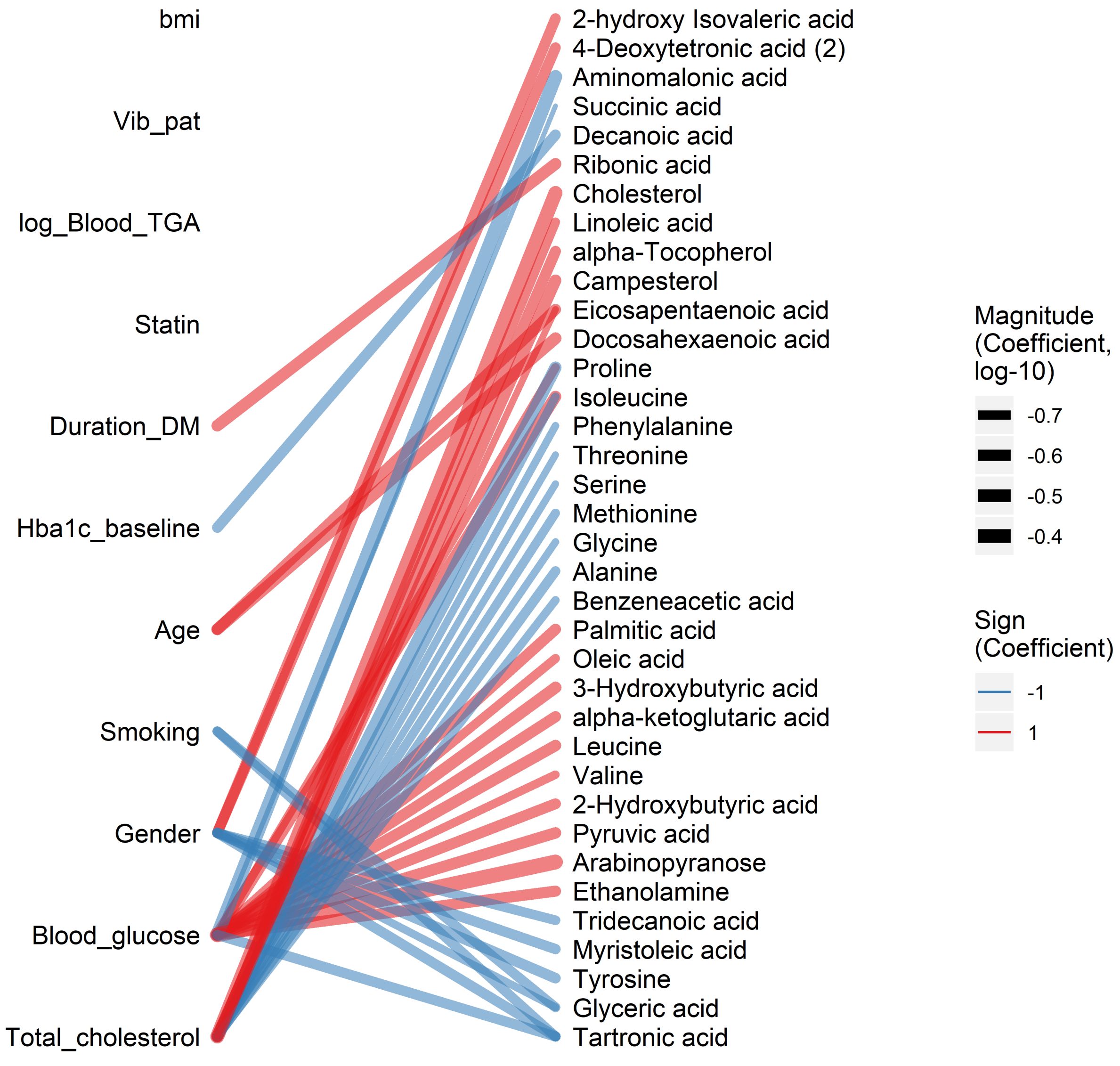

```
## [1] "bipartite_network_from_limma was created by Tommi Suvitaival"
## [1] "tommi.raimo.leo.suvitaival@regionh.dk"
## [1] "2019-05-06"

## Warning in if (drop.variables != "none") {: the condition has length > 1 and
## only the first element will be used

## Warning: Removed 4 rows containing missing values (geom_segment).

## Warning: Removed 1 rows containing missing values (geom_text).
```

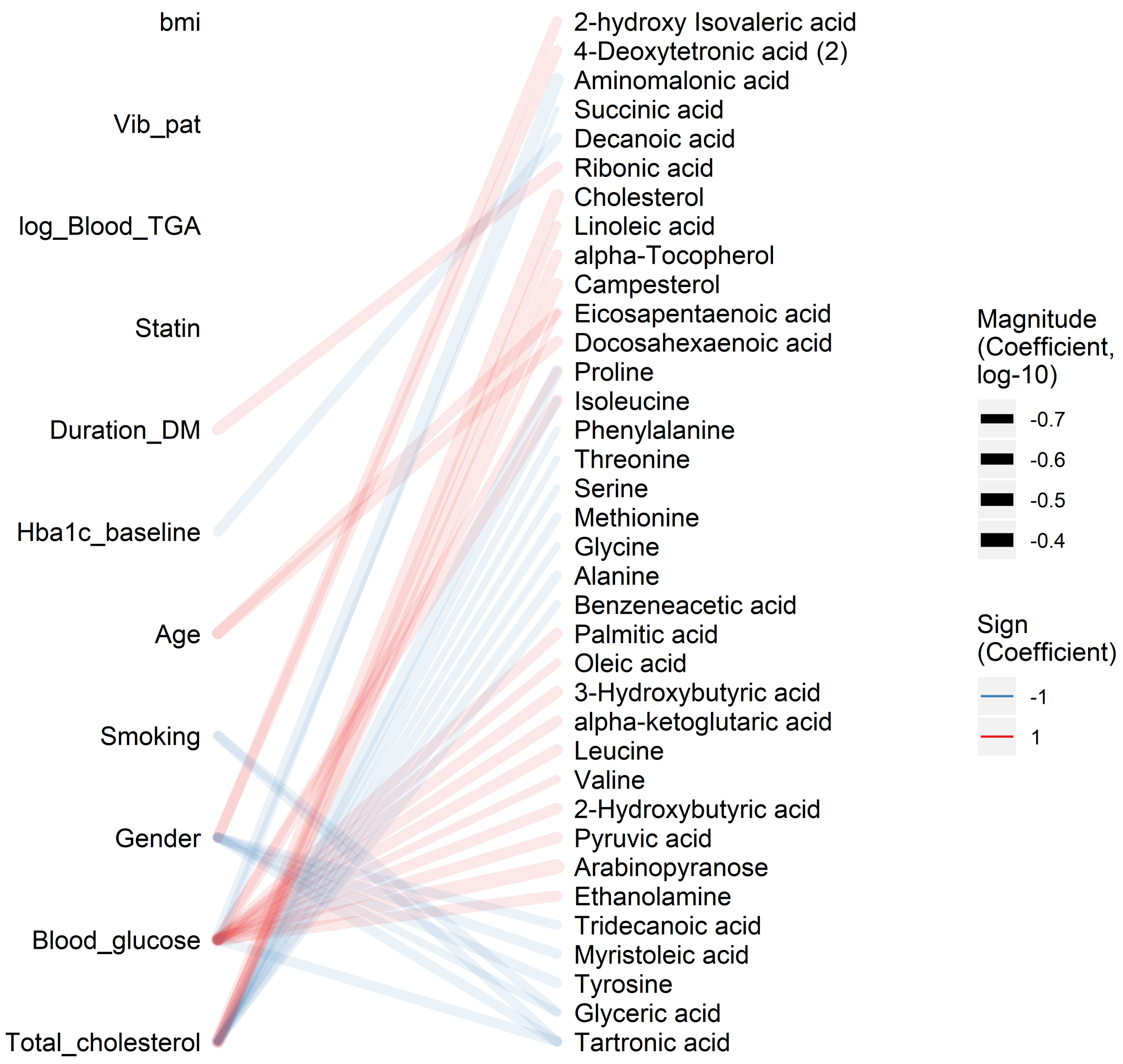

4.3 Fully Adjusted Model

```
## [1] "Fitting models:"
## [1] "~ Vib_pat + Age + bmi + Blood_glucose + Duration_DM + Gender + Hba1c_baseline + log_Blood_TGA +
## [1] ""
```

4.3.1 Tables of Model Coefficients

```
## [1] ""
## [1] "Table: Vib_pat"
## [1] "  (from model: "
## [1] "   ~ Vib_pat + Age + bmi + Blood_glucose + Duration_DM +"
## [1] "     Gender + Hba1c_baseline + log_Blood_TGA + Smoking + Statin +"
## [1] "     Total_cholesterol + egfr)"
## [1] ""
##                                Name Coefficient P.Value adj.P.Val
## 1       Arachidonic acid, TMS; 24    -0.16500  0.0128     0.610
## 2             Threonine, 3TMS; 12     0.13700  0.0385     0.610
## 3        Docosahexaenoic acid; 53    -0.13600  0.0386     0.610
## 4  2-Hydroxybutyric acid, 2TMS; 2    -0.13300  0.0432     0.610
## 5              Arachidic acid; 46    -0.12900  0.0511     0.610
## 6          Heptadecanoic acid; 60    -0.12500  0.0594     0.610
## 7            Nonadecanoic acid; 66    -0.12000  0.0698     0.610
## 8              1,3-Propanediol; 34    -0.11900  0.0709     0.610
## 9                 Ethanolamine; 56    -0.11800  0.0732     0.610
## 10 4-Hydroxyphenyllactic acid; 44     0.11500  0.0822     0.616
## 11                 Lactic acid; 29    -0.10700  0.1060     0.634
## 12                    Tyrosine; 75     0.10600  0.1090     0.634
## 13            Methionine, 2TMS; 16     0.10500  0.1100     0.634
## 14            Stearic acid, TMS; 2    -0.10200  0.1230     0.661
## 15 3-Hydroxybutyric acid, 2TMS; 1    -0.09160  0.1650     0.750
## 16              Hydroxyproline; 64     0.09040  0.1710     0.750
## 17          Heptadecanoic acid; 61    -0.09030  0.1720     0.750
## 18              L-5-Oxoproline; 63     0.08860  0.1800     0.750
## 19 alpha-ketoglutaric acid, TMS M    -0.08480  0.1990     0.784
## 20           Aminomalonic acid; 45    -0.08140  0.2160     0.811
## 21               Octanoic acid; 68    -0.07690  0.2440     0.849
## 22            Malic acid, 3TMS; 11    -0.07480  0.2570     0.849
## 23                     Ribitol; 71     0.07040  0.2830     0.849
## 24                Valine, 2TMS; 20     0.07070  0.2830     0.849
## 25               Decanoic acid; 52     0.06850  0.2990     0.849
## 26         Phenylalanine, 2TMS; 13     0.06340  0.3370     0.849
## 27     3-Indolepropionic acid; 41     0.06300  0.3400     0.849
## 28                Serine, 3TMS; 14     0.06120  0.3530     0.849
## 29                     Ribitol; 70     0.05960  0.3670     0.849
## 30           Linoleic acid, TMS; 4     0.05870  0.3740     0.849
## 31                    Glycerol; 57     0.05760  0.3830     0.849
## 32        3-Indoleacetic acid; 40    -0.05610  0.3960     0.849
## 33           Pyroglutamic acid; 69    -0.05590  0.3980     0.849
## 34                Pyruvic acid; 31    -0.05400  0.4130     0.849
## 35            alpha-Tocopherol; 26     0.05210  0.4290     0.849
## 36     4-Hydroxybutanoic acid; 43     0.05180  0.4340     0.849
## 37               Glycine, 3TMS; 17     0.04970  0.4520     0.849
## 38               Alanine, 2TMS; 25     0.04850  0.4630     0.849
## 39                 1-Dodecanol; 36    -0.04850  0.4630     0.849
## 40               Hydroxylamine; 62    -0.04750  0.4730     0.849
## 41                 Bisphenol A; 48    -0.04660  0.4810     0.849
## 42                    Glycerol; 58     0.04610  0.4860     0.849
## 43               1-Monopalmitin; 37     0.04600  0.4870     0.849
## 44               Nonanoic acid; 67    -0.04130  0.5330     0.909
```

```
## 45           Arabinopyranose; 51     0.03440  0.6000     0.930
## 46       2-Palmitoylglycerol; 39     0.03370  0.6100     0.930
## 47            Tartronic acid; 73    -0.03170  0.6300     0.930
## 48  2-hydroxy Isovaleric acid; 38    -0.03130  0.6350     0.930
## 49          Tridecanoic acid; 74     0.03050  0.6440     0.930
## 50             Leucine, 2TMS; 19    -0.02790  0.6730     0.930
## 51           Dodecanoic acid; 54     0.02790  0.6730     0.930
## 52          Isoleucine, 2TMS; 18    -0.02730  0.6790     0.930
## 53          Myristoleic acid; 65     0.02520  0.7030     0.930
## 54        Glyceryl-glycoside; 59     0.02490  0.7060     0.930
## 55            Oleic acid, TMS; 3    -0.02480  0.7070     0.930
## 56         Palmitic acid, TMS; 5    -0.02430  0.7120     0.930
## 57 4-Hydroxybenzeneacetic acid; 4    -0.02380  0.7180     0.930
## 58             Glyceric acid; 30    -0.02250  0.7330     0.930
## 59               Ribonic acid; 72    -0.02230  0.7340     0.930
## 60      4-Deoxytetronic acid; 32     0.02150  0.7440     0.930
## 61        Benzeneacetic acid; 47     0.01710  0.7960     0.937
## 62 3,4-Dihydroxybutanoic acid; 27    -0.01690  0.7960     0.937
## 63     Eicosapentaenoic acid; 55     0.01680  0.7980     0.937
## 64      4-Deoxytetronic acid; 33    -0.01670  0.7990     0.937
## 65        11-Eicosenoic acid; 35    -0.01260  0.8490     0.939
## 66          Myo inositol 6TMS; 1    -0.01130  0.8630     0.939
## 67        Glutamic acid, 3TMS; 8    -0.00937  0.8870     0.939
## 68 2,4-Dihydroxybutanoic acid; 28     0.00906  0.8900     0.939
## 69          Citric acid, 4TMS; 6    -0.00805  0.9030     0.939
## 70               Campesterol; 49    -0.00788  0.9050     0.939
## 71        Succinic acid, 2TMS; 7     0.00643  0.9230     0.939
## 72             Proline, 2TMS; 21    -0.00637  0.9230     0.939
## 73                Creatinine; 50     0.00613  0.9260     0.939
## 74         Fumaric acid, 2TMS; 9     0.00578  0.9300     0.939
## 75          Cholesterol, TMS; 23    -0.00498  0.9390     0.939
```

4.3.2 Forest Plot of Model Coefficients

```
## Warning: Ignoring unknown aesthetics: x

## Warning: Ignoring unknown aesthetics: x

## Warning: Ignoring unknown aesthetics: x

## Warning: Ignoring unknown aesthetics: x

## Warning: Ignoring unknown aesthetics: x

## Warning: Ignoring unknown aesthetics: x

## Warning: Ignoring unknown aesthetics: x
```

```
## NULL
```

4.3.3 Bipartite Network of Model Coefficients

```
## [1] "bipartite_network_from_limma was created by Tommi Suvitaival"
## [1] "tommi.raimo.leo.suvitaival@regionh.dk"
## [1] "2019-05-06"

## Warning in if (drop.variables != "none") {: the condition has length > 1 and
## only the first element will be used

## Warning: Removed 5 rows containing missing values (geom_segment).

## Warning: Removed 1 rows containing missing values (geom_text).
```

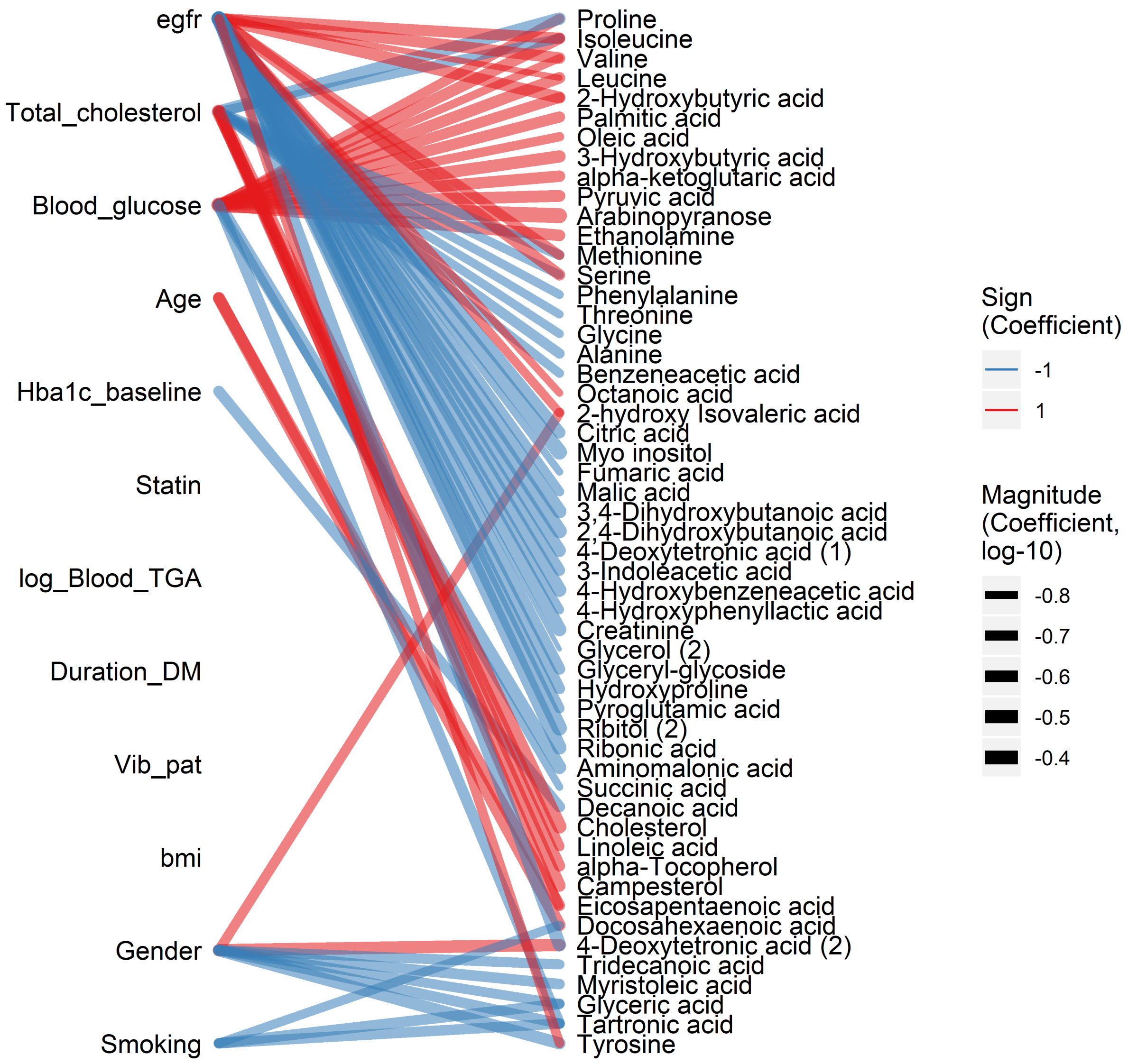

```
## [1] "bipartite_network_from_limma was created by Tommi Suvitaival"
## [1] "tommi.raimo.leo.suvitaival@regionh.dk"
## [1] "2019-05-06"

## Warning in if (drop.variables != "none") {: the condition has length > 1 and
## only the first element will be used

## Warning: Removed 5 rows containing missing values (geom_segment).

## Warning: Removed 1 rows containing missing values (geom_text).
```

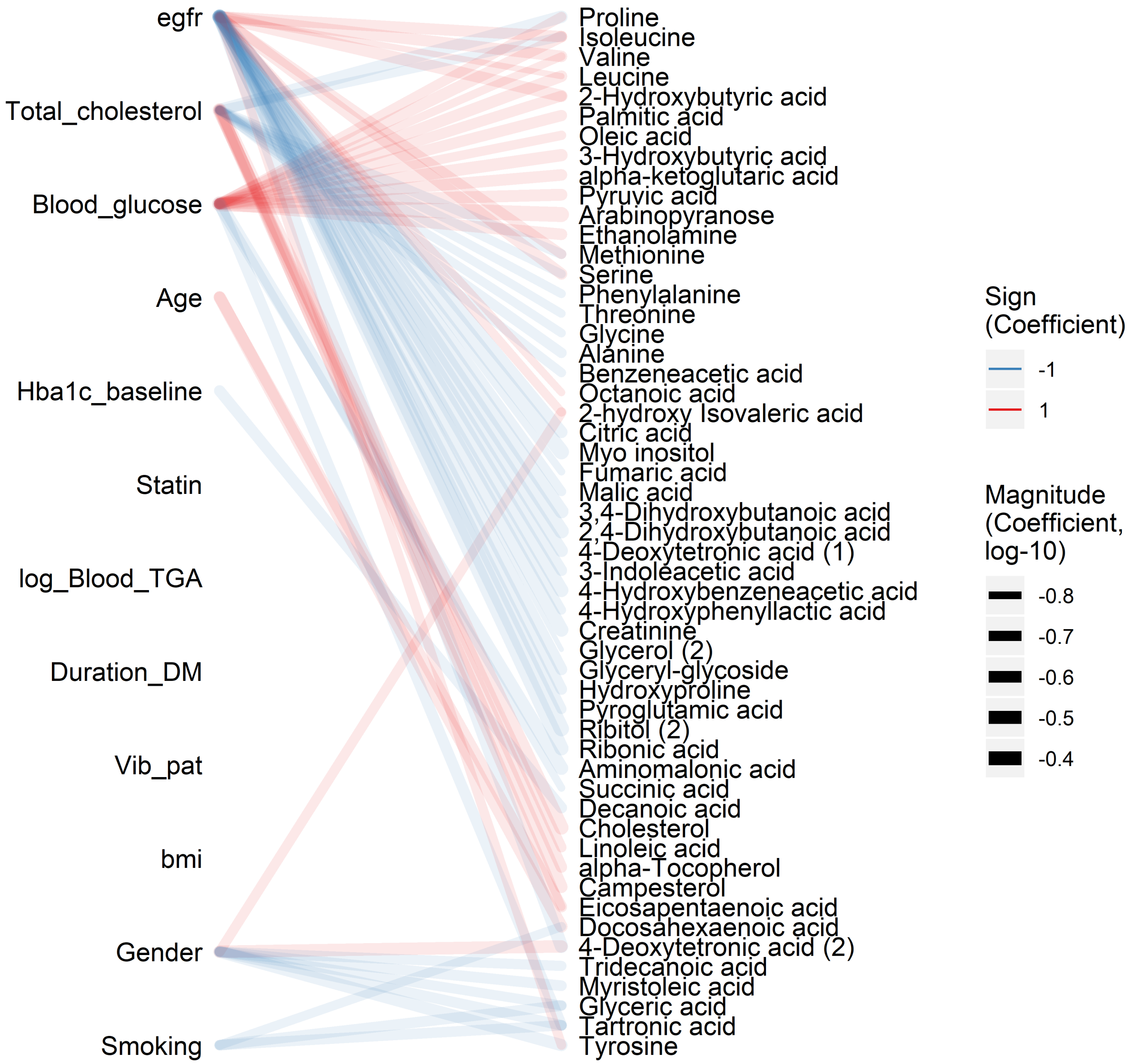

5 Secondary Analyses

5.1 Resting HR Vagus

5.1.1 Crude Model

```
## [1] "Fitting models:"
## [1] "~ rest_HR_vag"
## [1] ""
```

5.1.1.1 Tables of Model Coefficients

```
## [1] ""
## [1] "Table: rest_HR_vag"
## [1] "  (from model: "
## [1] "    ~ rest_HR_vag)"
## [1] ""
##                              Name Coefficient  P.Value adj.P.Val
## 1           Methionine, 2TMS; 16    -0.20500 0.000588    0.0441
## 2             Oleic acid, TMS; 3     0.17200 0.003820    0.1040
## 3            Threonine, 3TMS; 12    -0.17100 0.004140    0.1040
## 4  3,4-Dihydroxybutanoic acid; 27     0.15800 0.008000    0.1500
## 5        Docosahexaenoic acid; 53    -0.14900 0.012400    0.1660
## 6               Valine, 2TMS; 20    -0.14800 0.013300    0.1660
## 7  4-Hydroxyphenyllactic acid; 44     0.13800 0.020500    0.1860
## 8  2-Hydroxybutyric acid, 2TMS; 2     0.13600 0.022200    0.1860
## 9      3-Indolepropionic acid; 41    -0.13500 0.022900    0.1860
## 10        Succinic acid, 2TMS; 7     0.13300 0.025900    0.1860
## 11            Tartronic acid; 73    -0.13100 0.027200    0.1860
## 12           1,3-Propanediol; 34    -0.11500 0.053500    0.2840
## 13                   Ribitol; 71     0.11500 0.053600    0.2840
## 14         Palmitic acid, TMS; 5     0.11400 0.055300    0.2840
## 15               Lactic acid; 29     0.11300 0.056900    0.2840
## 16                  Glycerol; 57     0.11000 0.065800    0.2990
## 17 4-Hydroxybenzeneacetic acid; 4     0.10900 0.067700    0.2990
## 18          Stearic acid, TMS; 2     0.10600 0.074900    0.3120
## 19        11-Eicosenoic acid; 35     0.10400 0.079500    0.3140
## 20         Nonadecanoic acid; 66    -0.09580 0.108000    0.4040
## 21         Fumaric acid, 2TMS; 9     0.08680 0.145000    0.4830
## 22       Phenylalanine, 2TMS; 13    -0.08620 0.148000    0.4830
## 23              Serine, 3TMS; 14    -0.08540 0.151000    0.4830
## 24              Ethanolamine; 56    -0.08310 0.163000    0.4830
## 25             L-5-Oxoproline; 63    -0.08260 0.166000    0.4830
## 26 3-Hydroxybutyric acid, 2TMS; 1     0.08120 0.173000    0.4830
## 27             Leucine, 2TMS; 19    -0.07900 0.185000    0.4830
## 28 2,4-Dihydroxybutanoic acid; 28     0.07600 0.202000    0.4830
## 29     Eicosapentaenoic acid; 55    -0.07560 0.204000    0.4830
## 30          Myristoleic acid; 65     0.07550 0.205000    0.4830
## 31           Dodecanoic acid; 54     0.07490 0.209000    0.4830
## 32              Ribonic acid; 72     0.07390 0.214000    0.4830
## 33      4-Deoxytetronic acid; 33     0.07310 0.219000    0.4830
## 34          alpha-Tocopherol; 26    -0.07170 0.229000    0.4830
## 35        Glutamic acid, 3TMS; 8     0.07070 0.235000    0.4830
## 36          Myo inositol 6TMS; 1     0.07020 0.239000    0.4830
## 37                  Glycerol; 58     0.07020 0.239000    0.4830
## 38             Glyceric acid; 30    -0.06930 0.245000    0.4830
## 39                Creatinine; 50     0.06760 0.257000    0.4880
## 40               Campesterol; 49     0.06660 0.263000    0.4880
## 41     Arachidonic acid, TMS; 24     0.06620 0.267000    0.4880
## 42         Aminomalonic acid; 45    -0.06320 0.289000    0.5040
## 43        Heptadecanoic acid; 61     0.06310 0.289000    0.5040
## 44                   Ribitol; 70     0.06180 0.300000    0.5110
## 45        Heptadecanoic acid; 60     0.05380 0.366000    0.6060
## 46             Nonanoic acid; 67     0.05320 0.372000    0.6060
```

```
## 47                Bisphenol A; 48    -0.05010 0.400000    0.6390
## 48  2-hydroxy Isovaleric acid; 38     0.04790 0.421000    0.6580
## 49           Malic acid, 3TMS; 11     0.04700 0.430000    0.6590
## 50              Decanoic acid; 52     0.04600 0.440000    0.6600
## 51             Arachidic acid; 46     0.04430 0.457000    0.6720
## 52           Isoleucine, 2TMS; 18    -0.04140 0.487000    0.7020
## 53                1-Dodecanol; 36     0.03880 0.515000    0.7160
## 54                   Tyrosine; 75    -0.03870 0.516000    0.7160
## 55     4-Hydroxybutanoic acid; 43     0.03730 0.531000    0.7250
## 56       4-Deoxytetronic acid; 32    -0.03590 0.547000    0.7320
## 57              Octanoic acid; 68    -0.03420 0.566000    0.7450
## 58           Tridecanoic acid; 74    -0.03250 0.585000    0.7570
## 59              Hydroxylamine; 62    -0.03040 0.610000    0.7760
## 60              Alanine, 2TMS; 25    -0.02550 0.668000    0.8360
## 61         Benzeneacetic acid; 47    -0.02140 0.720000    0.8850
## 62              Proline, 2TMS; 21    -0.01970 0.741000    0.8940
## 63             Hydroxyproline; 64     0.01750 0.769000    0.8940
## 64            Arabinopyranose; 51    -0.01690 0.776000    0.8940
## 65           Citric acid, 4TMS; 6     0.01600 0.788000    0.8940
## 66          Pyroglutamic acid; 69    -0.01550 0.795000    0.8940
## 67              Glycine, 3TMS; 17    -0.01490 0.802000    0.8940
## 68        2-Palmitoylglycerol; 39    -0.01430 0.810000    0.8940
## 69               Pyruvic acid; 31    -0.01170 0.845000    0.9050
## 70           Cholesterol, TMS; 23     0.01110 0.852000    0.9050
## 71 alpha-ketoglutaric acid, TMS M    -0.01010 0.866000    0.9050
## 72         Glyceryl-glycoside; 59     0.00982 0.869000    0.9050
## 73        3-Indoleacetic acid; 40    -0.00800 0.893000    0.9180
## 74          Linoleic acid, TMS; 4    -0.00385 0.948000    0.9610
## 75             1-Monopalmitin; 37     0.00277 0.963000    0.9630
```

5.1.1.2 Forest Plot of Model Coefficients

```
## Warning: Ignoring unknown aesthetics: x
```

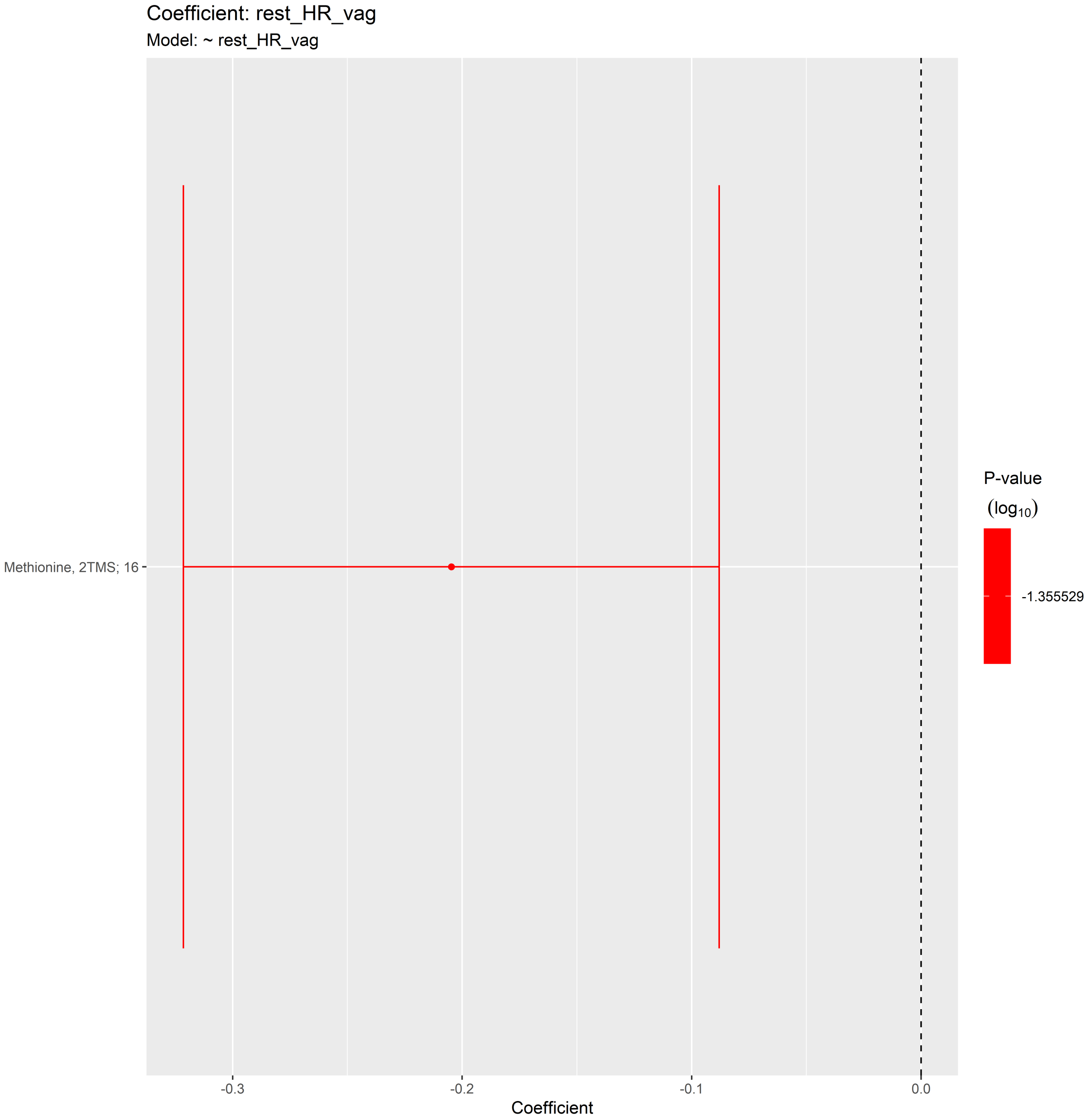

5.1.2 Adjusted Model

```
## [1] "Fitting models:"
## [1] "~ rest_HR_vag + Age + bmi + Blood_glucose + Duration_DM + Gender + Hba1c_baseline + log_Blood_T
## [1] ""
```

5.1.2.1 Tables of Model Coefficients

```
## [1] ""
## [1] "Table: rest_HR_vag"
## [1] "  (from model: "
## [1] "   ~ rest_HR_vag + Age + bmi + Blood_glucose + Duration_DM +"
## [1] "     Gender + Hba1c_baseline + log_Blood_TGA + Smoking + Statin +"
## [1] "     Total_cholesterol)"
## [1] ""
##                               Name Coefficient P.Value adj.P.Val
## 1             Threonine, 3TMS; 12    -0.18600 0.00280     0.102
## 2            Methionine, 2TMS; 16    -0.18200 0.00337     0.102
## 3              Oleic acid, TMS; 3     0.17800 0.00410     0.102
## 4  4-Hydroxyphenyllactic acid; 44     0.16400 0.00853     0.159
## 5                Valine, 2TMS; 20    -0.15500 0.01260     0.159
## 6          Succinic acid, 2TMS; 7     0.15500 0.01270     0.159
## 7           Palmitic acid, TMS; 5     0.13900 0.02550     0.273
## 8                    Glycerol; 58     0.13400 0.03130     0.293
## 9          11-Eicosenoic acid; 35     0.12300 0.04810     0.390
## 10         Heptadecanoic acid; 61     0.11900 0.05550     0.390
## 11 4-Hydroxybenzeneacetic acid; 4     0.11400 0.06620     0.390
## 12           Stearic acid, TMS; 2     0.11100 0.07510     0.390
## 13                Lactic acid; 29     0.11100 0.07530     0.390
## 14              Leucine, 2TMS; 19    -0.11000 0.07640     0.390
## 15 2-Hydroxybutyric acid, 2TMS; 2     0.11000 0.07810     0.390
## 16                    Ribitol; 71     0.10400 0.09300     0.436
## 17 3,4-Dihydroxybutanoic acid; 27     0.10000 0.10700     0.470
## 18  2-hydroxy Isovaleric acid; 38     0.09610 0.12200     0.496
## 19        Phenylalanine, 2TMS; 13    -0.09130 0.14200     0.496
## 20       Docosahexaenoic acid; 53    -0.09050 0.14500     0.496
## 21               Serine, 3TMS; 14    -0.08950 0.15000     0.496
## 22           Malic acid, 3TMS; 11     0.08930 0.15100     0.496
## 23     3-Indolepropionic acid; 41    -0.08900 0.15200     0.496
## 24             Tartronic acid; 73    -0.08590 0.16700     0.512
## 25         Fumaric acid, 2TMS; 9     0.08510 0.17100     0.512
## 26               Ethanolamine; 56    -0.08350 0.17900     0.517
## 27 3-Hydroxybutyric acid, 2TMS; 1     0.07950 0.20100     0.541
## 28           Myristoleic acid; 65     0.07800 0.20900     0.541
## 29              Hydroxylamine; 62    -0.07750 0.21300     0.541
## 30       4-Deoxytetronic acid; 32    -0.07680 0.21600     0.541
## 31              Nonanoic acid; 67     0.07550 0.22500     0.543
## 32            Dodecanoic acid; 54     0.07420 0.23300     0.545
## 33                   Tyrosine; 75    -0.07020 0.25900     0.561
## 34                   Glycerol; 57     0.06930 0.26500     0.561
## 35            1,3-Propanediol; 34    -0.06910 0.26600     0.561
## 36          Nonadecanoic acid; 66    -0.06830 0.27200     0.561
## 37            Arabinopyranose; 51    -0.06580 0.29000     0.561
## 38           alpha-Tocopherol; 26    -0.06550 0.29200     0.561
## 39           Citric acid, 4TMS; 6     0.06460 0.29900     0.561
## 40             Arachidic acid; 46     0.06450 0.29900     0.561
## 41      Arachidonic acid, TMS; 24     0.06210 0.31800     0.582
## 42         Heptadecanoic acid; 60     0.05860 0.34600     0.611
## 43              Decanoic acid; 52     0.05800 0.35000     0.611
## 44           Isoleucine, 2TMS; 18    -0.05450 0.38000     0.632
```

```
## 45           Myo inositol 6TMS; 1     0.05430 0.38200     0.632
## 46             L-5-Oxoproline; 63    -0.05370 0.38800     0.632
## 47                1-Dodecanol; 36     0.05090 0.41300     0.647
## 48               Ribonic acid; 72     0.05080 0.41400     0.647
## 49              Alanine, 2TMS; 25    -0.04490 0.47000     0.719
## 50          Glyceryl-glycoside; 59    -0.03990 0.52100     0.754
## 51 2,4-Dihydroxybutanoic acid; 28     0.03970 0.52300     0.754
## 52                Bisphenol A; 48    -0.03970 0.52300     0.754
## 53          Benzeneacetic acid; 47     0.03830 0.53800     0.758
## 54              Proline, 2TMS; 21    -0.03750 0.54600     0.758
## 55        4-Deoxytetronic acid; 33     0.03630 0.55900     0.763
## 56             1-Monopalmitin; 37     0.03390 0.58500     0.784
## 57                Campesterol; 49     0.03100 0.61800     0.796
## 58     4-Hydroxybutanoic acid; 43     0.03020 0.62700     0.796
## 59                 Creatinine; 50     0.03020 0.62700     0.796
## 60      Eicosapentaenoic acid; 55    -0.02940 0.63700     0.796
## 61              Octanoic acid; 68    -0.02740 0.65900     0.802
## 62           Tridecanoic acid; 74    -0.02710 0.66300     0.802
## 63              Glycine, 3TMS; 17     0.02250 0.71700     0.854
## 64 alpha-ketoglutaric acid, TMS M    -0.02000 0.74800     0.876
## 65              Glyceric acid; 30    -0.01700 0.78500     0.906
## 66           Pyroglutamic acid; 69    -0.01350 0.82800     0.941
## 67         3-Indoleacetic acid; 40    -0.01220 0.84400     0.945
## 68               Pyruvic acid; 31    -0.01070 0.86300     0.952
## 69             Hydroxyproline; 64     0.00942 0.88000     0.956
## 70           Aminomalonic acid; 45    -0.00787 0.89900     0.957
## 71         2-Palmitoylglycerol; 39    -0.00732 0.90600     0.957
## 72          Glutamic acid, 3TMS; 8     0.00611 0.92200     0.960
## 73            Cholesterol, TMS; 23     0.00414 0.94700     0.973
## 74           Linoleic acid, TMS; 4     0.00233 0.97000     0.983
## 75                     Ribitol; 70    -0.00113 0.98500     0.985
```

5.1.2.2 Forest Plot of Model Coefficients

```
## Warning: Ignoring unknown aesthetics: x

## Warning: Ignoring unknown aesthetics: x

## Warning: Ignoring unknown aesthetics: x

## Warning: Ignoring unknown aesthetics: x

## Warning: Ignoring unknown aesthetics: x

## Warning: Ignoring unknown aesthetics: x

## NULL
```

5.1.3 Fully-Adjusted Model

```
## [1] "Fitting models:"
## [1] "~ rest_HR_vag + Age + bmi + Blood_glucose + Duration_DM + Gender + Hba1c_baseline + log_Blood_TG
## [1] ""
```

5.1.3.1 Tables of Model Coefficients

```
## [1] ""
## [1] "Table: rest_HR_vag"
## [1] "  (from model: "
## [1] "   ~ rest_HR_vag + Age + bmi + Blood_glucose + Duration_DM +"
## [1] "     Gender + Hba1c_baseline + log_Blood_TGA + Smoking + Statin +"
## [1] "     Total_cholesterol + egfr)"
## [1] ""
##                               Name Coefficient P.Value adj.P.Val
## 1              Oleic acid, TMS; 3     0.18000 0.00334     0.128
## 2             Threonine, 3TMS; 12    -0.18000 0.00340     0.128
## 3            Methionine, 2TMS; 16    -0.17000 0.00556     0.139
## 4           Succinic acid, 2TMS; 7     0.15400 0.01250     0.221
## 5  4-Hydroxyphenyllactic acid; 44     0.14900 0.01530     0.221
## 6           Palmitic acid, TMS; 5     0.14600 0.01770     0.221
## 7                Valine, 2TMS; 20    -0.14000 0.02210     0.236
## 8  2-Hydroxybutyric acid, 2TMS; 2     0.13200 0.03100     0.290
## 9                    Glycerol; 58     0.12400 0.04340     0.311
## 10         Heptadecanoic acid; 61     0.12300 0.04560     0.311
## 11         11-Eicosenoic acid; 35     0.12100 0.04850     0.311
## 12           Stearic acid, TMS; 2     0.12100 0.04970     0.311
## 13                Lactic acid; 29     0.11600 0.05920     0.342
## 14  2-hydroxy Isovaleric acid; 38     0.10900 0.07580     0.406
## 15       4-Deoxytetronic acid; 32    -0.10200 0.09760     0.479
## 16              Leucine, 2TMS; 19    -0.10000 0.10200     0.479
## 17        Phenylalanine, 2TMS; 13    -0.09560 0.12000     0.530
## 18     3-Indolepropionic acid; 41    -0.08920 0.14700     0.540
## 19            Tartronic acid; 73    -0.08720 0.15400     0.540
## 20 4-Hydroxybenzeneacetic acid; 4     0.08630 0.15900     0.540
## 21              Hydroxylamine; 62    -0.08600 0.16200     0.540
## 22       Docosahexaenoic acid; 53    -0.08210 0.18000     0.540
## 23 3-Hydroxybutyric acid, 2TMS; 1     0.08030 0.19100     0.540
## 24           Malic acid, 3TMS; 11     0.07800 0.20500     0.540
## 25                   Glycerol; 57     0.07780 0.20600     0.540
## 26               Ethanolamine; 56    -0.07760 0.20700     0.540
## 27            Dodecanoic acid; 54     0.07670 0.21200     0.540
## 28           Myristoleic acid; 65     0.07640 0.21400     0.540
## 29              Nonanoic acid; 67     0.07470 0.22600     0.540
## 30          Fumaric acid, 2TMS; 9     0.07370 0.23100     0.540
## 31               Serine, 3TMS; 14    -0.07110 0.24600     0.540
## 32 3,4-Dihydroxybutanoic acid; 27     0.07070 0.24700     0.540
## 33            Arabinopyranose; 51    -0.06920 0.25700     0.540
## 34                    Ribitol; 71     0.06850 0.26200     0.540
## 35             Arachidic acid; 46     0.06900 0.26200     0.540
## 36      Arachidonic acid, TMS; 24     0.06860 0.26500     0.540
## 37            1,3-Propanediol; 34    -0.06840 0.26700     0.540
## 38          Nonadecanoic acid; 66    -0.06410 0.29800     0.576
## 39           alpha-Tocopherol; 26    -0.06360 0.29900     0.576
## 40              Decanoic acid; 52     0.06130 0.31700     0.595
## 41                   Tyrosine; 75    -0.05840 0.34200     0.601
## 42         Glyceryl-glycoside; 59    -0.05820 0.34300     0.601
## 43         Heptadecanoic acid; 60     0.05820 0.34500     0.601
## 44                1-Dodecanol; 36     0.05240 0.39500     0.665
```

```
## 45              L-5-Oxoproline; 63    -0.05190 0.39900     0.665
## 46               Alanine, 2TMS; 25    -0.04630 0.45100     0.735
## 47            Citric acid, 4TMS; 6     0.04500 0.46300     0.739
## 48               Proline, 2TMS; 21    -0.04200 0.49300     0.771
## 49            Isoleucine, 2TMS; 18    -0.03800 0.53500     0.807
## 50                 Bisphenol A; 48    -0.03780 0.54000     0.807
## 51          Benzeneacetic acid; 47     0.03690 0.54900     0.807
## 52                 Campesterol; 49     0.03220 0.59900     0.859
## 53              1-Monopalmitin; 37     0.03160 0.60700     0.859
## 54            Tridecanoic acid; 74    -0.02940 0.63300     0.873
## 55         3-Indoleacetic acid; 40    -0.02870 0.64000     0.873
## 56            Pyroglutamic acid; 69    -0.02640 0.66800     0.895
## 57      4-Hydroxybutanoic acid; 43     0.02540 0.68100     0.895
## 58       Eicosapentaenoic acid; 55    -0.02160 0.72400     0.922
## 59                Ribonic acid; 72     0.02130 0.72700     0.922
## 60 alpha-ketoglutaric acid, TMS M    -0.02060 0.73800     0.922
## 61            Myo inositol 6TMS; 1     0.01750 0.77400     0.942
## 62          Glutamic acid, 3TMS; 8     0.01680 0.78500     0.942
## 63               Octanoic acid; 68    -0.01630 0.79100     0.942
## 64            Cholesterol, TMS; 23     0.01500 0.80600     0.945
## 65               Glycine, 3TMS; 17     0.01380 0.82300     0.949
## 66        4-Deoxytetronic acid; 33     0.01240 0.84000     0.954
## 67                Pyruvic acid; 31    -0.00904 0.88300     0.954
## 68              Hydroxyproline; 64    -0.00891 0.88500     0.954
## 69                     Ribitol; 70    -0.00888 0.88500     0.954
## 70               Glyceric acid; 30    -0.00715 0.90700     0.954
## 71            Aminomalonic acid; 45    -0.00684 0.91100     0.954
## 72         2-Palmitoylglycerol; 39    -0.00628 0.91900     0.954
## 73 2,4-Dihydroxybutanoic acid; 28     0.00548 0.92800     0.954
## 74                  Creatinine; 50     0.00234 0.97000     0.983
## 75            Linoleic acid, TMS; 4    -0.00042 0.99500     0.995
```

5.1.3.2 Forest Plot of Model Coefficients

```
## Warning: Ignoring unknown aesthetics: x

## Warning: Ignoring unknown aesthetics: x

## Warning: Ignoring unknown aesthetics: x

## Warning: Ignoring unknown aesthetics: x

## Warning: Ignoring unknown aesthetics: x

## Warning: Ignoring unknown aesthetics: x

## Warning: Ignoring unknown aesthetics: x

## Warning: Ignoring unknown aesthetics: x
```

```
## NULL
```

5.2 Deep Breathing (E_I)

5.2.1 Crude Model

```
## [1] "Fitting models:"
## [1] "~ E_I"
## [1] ""
```

5.2.1.1 Tables of Model Coefficients

```
## [1] ""
## [1] "Table: E_I"
## [1] "  (from model: "
## [1] "   ~ E_I)"
## [1] ""
##                               Name Coefficient  P.Value adj.P.Val
## 1  2,4-Dihydroxybutanoic acid; 28    -0.24900 2.85e-05  0.000974
## 2  3,4-Dihydroxybutanoic acid; 27    -0.24700 3.35e-05  0.000974
## 3                Ribonic acid; 72    -0.24500 3.90e-05  0.000974
## 4            Myo inositol 6TMS; 1    -0.22800 1.23e-04  0.002300
## 5            Citric acid, 4TMS; 6    -0.21300 3.46e-04  0.005190
## 6                  Creatinine; 50    -0.20100 7.28e-04  0.009100
## 7         4-Deoxytetronic acid; 32    -0.19600 9.75e-04  0.010400
## 8  4-Hydroxybenzeneacetic acid; 4    -0.18100 2.37e-03  0.022200
## 9                     Ribitol; 71    -0.16800 4.77e-03  0.039800
## 10         Glyceryl-glycoside; 59    -0.16000 7.00e-03  0.052500
## 11 4-Hydroxyphenyllactic acid; 44    -0.13400 2.44e-02  0.166000
## 12            Tridecanoic acid; 74    -0.12700 3.33e-02  0.208000
## 13           Fumaric acid, 2TMS; 9    -0.12300 3.87e-02  0.223000
## 14                   Glycerol; 58    -0.11900 4.61e-02  0.247000
## 15                    Ribitol; 70    -0.11200 5.97e-02  0.298000
## 16              1-Monopalmitin; 37    -0.10800 6.87e-02  0.322000
## 17         Succinic acid, 2TMS; 7    -0.10600 7.49e-02  0.330000
## 18      Arachidonic acid, TMS; 24    -0.10400 7.92e-02  0.330000
## 19           Methionine, 2TMS; 16     0.09770 1.01e-01  0.397000
## 20           alpha-Tocopherol; 26    -0.09360 1.15e-01  0.421000
## 21           Myristoleic acid; 65    -0.09300 1.18e-01  0.421000
## 22              Oleic acid, TMS; 3    -0.08980 1.31e-01  0.447000
## 23                Campesterol; 49    -0.07980 1.80e-01  0.540000
## 24              Hydroxyproline; 64    -0.07970 1.80e-01  0.540000
## 25           Linoleic acid, TMS; 4    -0.07930 1.83e-01  0.540000
## 26             Tartronic acid; 73     0.07850 1.87e-01  0.540000
## 27              Proline, 2TMS; 21    -0.07300 2.20e-01  0.611000
## 28           Malic acid, 3TMS; 11    -0.06930 2.44e-01  0.651000
## 29              Octanoic acid; 68     0.06780 2.54e-01  0.651000
## 30         Heptadecanoic acid; 61    -0.06670 2.62e-01  0.651000
## 31     3-Indolepropionic acid; 41     0.06460 2.77e-01  0.651000
## 32     4-Hydroxybutanoic acid; 43    -0.06170 2.99e-01  0.651000
## 33            Arabinopyranose; 51    -0.06090 3.06e-01  0.651000
## 34              L-5-Oxoproline; 63    -0.06020 3.12e-01  0.651000
## 35           Palmitic acid, TMS; 5    -0.05930 3.19e-01  0.651000
## 36 2-Hydroxybutyric acid, 2TMS; 2    -0.05910 3.21e-01  0.651000
## 37              Glycine, 3TMS; 17    -0.05900 3.21e-01  0.651000
## 38         Glutamic acid, 3TMS; 8    -0.05800 3.30e-01  0.651000
## 39                1-Dodecanol; 36    -0.05610 3.45e-01  0.664000
## 40               Serine, 3TMS; 14     0.05430 3.61e-01  0.670000
## 41 3-Hydroxybutyric acid, 2TMS; 1    -0.05370 3.67e-01  0.670000
## 42           Pyroglutamic acid; 69    -0.04990 4.01e-01  0.677000
## 43                   Tyrosine; 75     0.04890 4.11e-01  0.677000
## 44             1,3-Propanediol; 34     0.04870 4.13e-01  0.677000
## 45          Benzeneacetic acid; 47    -0.04860 4.14e-01  0.677000
## 46        Docosahexaenoic acid; 53     0.04840 4.15e-01  0.677000
```

```
## 47                 Bisphenol A; 48    -0.04340 4.66e-01  0.743000
## 48       4-Deoxytetronic acid; 33    -0.03850 5.18e-01  0.809000
## 49            Threonine, 3TMS; 12    -0.03720 5.32e-01  0.815000
## 50               Valine, 2TMS; 20     0.03550 5.50e-01  0.824000
## 51               Ethanolamine; 56     0.03460 5.61e-01  0.824000
## 52           Cholesterol, TMS; 23    -0.03170 5.94e-01  0.852000
## 53          Nonadecanoic acid; 66     0.03100 6.03e-01  0.852000
## 54            Dodecanoic acid; 54    -0.02980 6.16e-01  0.852000
## 55  2-hydroxy Isovaleric acid; 38    -0.02730 6.46e-01  0.852000
## 56             Arachidic acid; 46     0.02660 6.55e-01  0.852000
## 57           Stearic acid, TMS; 2    -0.02580 6.64e-01  0.852000
## 58              Nonanoic acid; 67    -0.02460 6.79e-01  0.852000
## 59              Glyceric acid; 30     0.02410 6.85e-01  0.852000
## 60     Eicosapentaenoic acid; 55     0.02220 7.09e-01  0.852000
## 61       Phenylalanine, 2TMS; 13     0.02200 7.11e-01  0.852000
## 62                Lactic acid; 29     0.02160 7.16e-01  0.852000
## 63       3-Indoleacetic acid; 40    -0.02040 7.32e-01  0.852000
## 64              Leucine, 2TMS; 19    -0.02010 7.35e-01  0.852000
## 65                   Glycerol; 57     0.01980 7.39e-01  0.852000
## 66       2-Palmitoylglycerol; 39    -0.01560 7.93e-01  0.895000
## 67               Pyruvic acid; 31     0.01510 8.00e-01  0.895000
## 68        11-Eicosenoic acid; 35     0.01250 8.33e-01  0.911000
## 69 alpha-ketoglutaric acid, TMS M    -0.01140 8.48e-01  0.911000
## 70              Hydroxylamine; 62     0.01120 8.50e-01  0.911000
## 71             Decanoic acid; 52    -0.01020 8.64e-01  0.912000
## 72         Heptadecanoic acid; 60     0.00782 8.95e-01  0.927000
## 73              Alanine, 2TMS; 25     0.00729 9.02e-01  0.927000
## 74          Aminomalonic acid; 45     0.00393 9.47e-01  0.960000
## 75           Isoleucine, 2TMS; 18    -0.00151 9.80e-01  0.980000
```

5.2.1.2 Forest Plot of Model Coefficients

```
## Warning: Ignoring unknown aesthetics: x
```

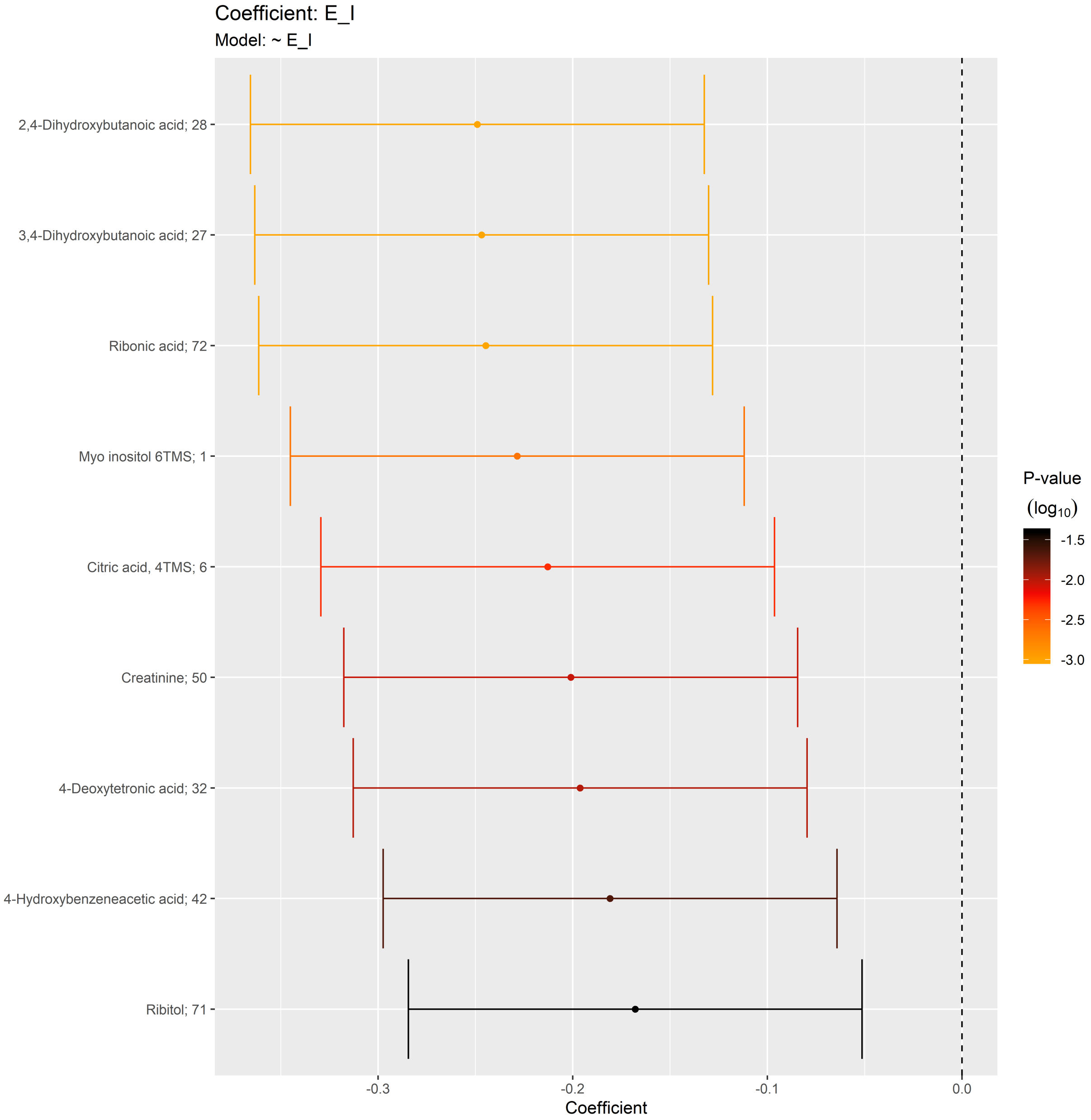

5.2.2 Adjusted Model

```
## [1] "Fitting models:"
## [1] "~ E_I + Age + bmi + Blood_glucose + Duration_DM + Gender + Hba1c_baseline + log_Blood_TGA + Smok
## [1] ""
```

5.2.2.1 Tables of Model Coefficients

```
## [1] ""
## [1] "Table: E_I"
## [1] "  (from model: "
## [1] "    ~ E_I + Age + bmi + Blood_glucose + Duration_DM + Gender"
## [1] "      + Hba1c_baseline + log_Blood_TGA + Smoking + Statin +"
## [1] "      Total_cholesterol)"
## [1] ""
##                                Name Coefficient  P.Value adj.P.Val
## 1                  Ribonic acid; 72   -0.219000 0.000682    0.0486
## 2              Citric acid, 4TMS; 6   -0.207000 0.001300    0.0486
## 3  2,4-Dihydroxybutanoic acid; 28   -0.198000 0.002100    0.0526
## 4                    Creatinine; 50   -0.176000 0.006270    0.1170
## 5              Myo inositol 6TMS; 1   -0.160000 0.012700    0.1500
## 6              Tridecanoic acid; 74   -0.159000 0.013500    0.1500
## 7          4-Deoxytetronic acid; 32   -0.158000 0.014000    0.1500
## 8  3,4-Dihydroxybutanoic acid; 27   -0.153000 0.017400    0.1630
## 9            Linoleic acid, TMS; 4   -0.123000 0.055200    0.4600
## 10      4-Hydroxybutanoic acid; 43   -0.115000 0.074200    0.5160
## 11          Glyceryl-glycoside; 59   -0.114000 0.075700    0.5160
## 12             Threonine, 3TMS; 12   -0.108000 0.092200    0.5560
## 13              1-Monopalmitin; 37   -0.107000 0.096700    0.5560
## 14 4-Hydroxybenzeneacetic acid; 4   -0.102000 0.113000    0.5560
## 15          Succinic acid, 2TMS; 7   -0.101000 0.115000    0.5560
## 16                 Bisphenol A; 48   -0.099400 0.122000    0.5560
## 17              Hydroxyproline; 64   -0.096600 0.133000    0.5560
## 18                    Glycerol; 58   -0.094100 0.144000    0.5560
## 19         Heptadecanoic acid; 61   -0.090300 0.161000    0.5560
## 20            Methionine, 2TMS; 16    0.088400 0.170000    0.5560
## 21              Tartronic acid; 73    0.085200 0.186000    0.5560
## 22        Docosahexaenoic acid; 53    0.082900 0.198000    0.5560
## 23                 1-Dodecanol; 36   -0.081600 0.205000    0.5560
## 24            Myristoleic acid; 65   -0.081300 0.207000    0.5560
## 25              Oleic acid, TMS; 3   -0.081200 0.207000    0.5560
## 26                    Tyrosine; 75    0.080600 0.211000    0.5560
## 27            Cholesterol, TMS; 23   -0.079900 0.215000    0.5560
## 28               Nonanoic acid; 67   -0.078900 0.220000    0.5560
## 29       Eicosapentaenoic acid; 55    0.078500 0.223000    0.5560
## 30       Arachidonic acid, TMS; 24   -0.078200 0.225000    0.5560
## 31          Fumaric acid, 2TMS; 9   -0.077300 0.230000    0.5560
## 32              Glycine, 3TMS; 17   -0.074000 0.251000    0.5790
## 33          Palmitic acid, TMS; 5   -0.073200 0.255000    0.5790
## 34 4-Hydroxyphenyllactic acid; 44   -0.072100 0.262000    0.5790
## 35              L-5-Oxoproline; 63   -0.070900 0.271000    0.5800
## 36                     Ribitol; 71   -0.067900 0.292000    0.6070
## 37            Malic acid, 3TMS; 11   -0.061900 0.336000    0.6820
## 38              Proline, 2TMS; 21   -0.060600 0.347000    0.6840
## 39  2-hydroxy Isovaleric acid; 38   -0.058900 0.360000    0.6920
## 40          11-Eicosenoic acid; 35    0.057000 0.376000    0.7040
## 41               Hydroxylamine; 62    0.055900 0.385000    0.7040
## 42                 Campesterol; 49   -0.050800 0.430000    0.7680
## 43               Octanoic acid; 68    0.046600 0.469000    0.7900
## 44            Arabinopyranose; 51   -0.046200 0.473000    0.7900
```

```
## 45                Pyruvic acid; 31    0.045100 0.483000    0.7900
## 46                     Ribitol; 70   -0.043200 0.503000    0.7900
## 47        3-Indoleacetic acid; 40    0.040300 0.532000    0.7900
## 48          Aminomalonic acid; 45   -0.040100 0.534000    0.7900
## 49         Glutamic acid, 3TMS; 8   -0.039600 0.539000    0.7900
## 50       4-Deoxytetronic acid; 33   -0.039500 0.539000    0.7900
## 51              Alanine, 2TMS; 25    0.039000 0.544000    0.7900
## 52 2-Hydroxybutyric acid, 2TMS; 2   -0.037300 0.562000    0.7900
## 53     3-Indolepropionic acid; 41    0.037300 0.562000    0.7900
## 54         Benzeneacetic acid; 47   -0.036700 0.569000    0.7900
## 55 alpha-ketoglutaric acid, TMS M    0.034500 0.592000    0.7950
## 56                 Lactic acid; 29    0.034300 0.594000    0.7950
## 57 3-Hydroxybutyric acid, 2TMS; 1   -0.033300 0.605000    0.7960
## 58              Decanoic acid; 52   -0.031300 0.627000    0.8100
## 59          Nonadecanoic acid; 66    0.028800 0.655000    0.8240
## 60          Pyroglutamic acid; 69   -0.028400 0.659000    0.8240
## 61            1,3-Propanediol; 34    0.023700 0.713000    0.8760
## 62             Stearic acid, TMS; 2   -0.021000 0.744000    0.9000
## 63         Heptadecanoic acid; 60   -0.016800 0.794000    0.9270
## 64              Glyceric acid; 30    0.015800 0.806000    0.9270
## 65               Valine, 2TMS; 20    0.015000 0.816000    0.9270
## 66             Arachidic acid; 46    0.014300 0.824000    0.9270
## 67                   Glycerol; 57    0.014000 0.828000    0.9270
## 68            alpha-Tocopherol; 26   -0.012700 0.843000    0.9300
## 69           Isoleucine, 2TMS; 18    0.010200 0.874000    0.9350
## 70              Leucine, 2TMS; 19   -0.009470 0.883000    0.9350
## 71                Ethanolamine; 56    0.009310 0.885000    0.9350
## 72             Dodecanoic acid; 54   -0.007530 0.907000    0.9450
## 73        Phenylalanine, 2TMS; 13   -0.001640 0.980000    0.9970
## 74               Serine, 3TMS; 14    0.001230 0.985000    0.9970
## 75        2-Palmitoylglycerol; 39    0.000248 0.997000    0.9970
```

5.2.2.2 Forest Plot of Model Coefficients

```
## Warning: Ignoring unknown aesthetics: x

## Warning: Ignoring unknown aesthetics: x

## Warning: Ignoring unknown aesthetics: x

## Warning: Ignoring unknown aesthetics: x

## Warning: Ignoring unknown aesthetics: x

## Warning: Ignoring unknown aesthetics: x

## Warning: Ignoring unknown aesthetics: x

## Warning: Ignoring unknown aesthetics: x
```

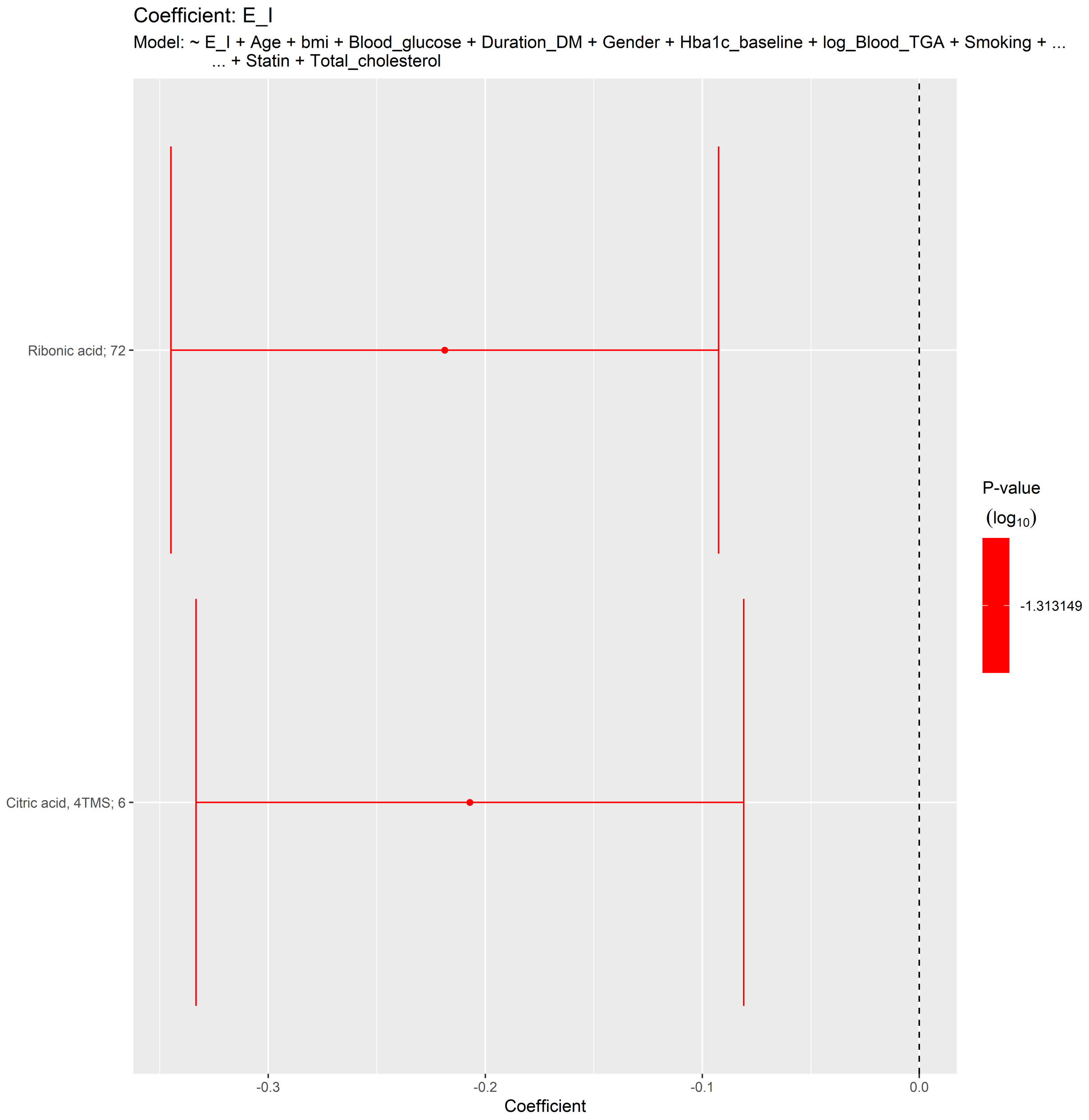

Coefficient: E_I
Model: ~ E_I + Age + bmi + Blood_glucose + Duration_DM + Gender + Hba1c_baseline + log_Blood_TGA + Smoking + ...
... + Statin + Total_cholesterol
Ribonic acid; 72
Citric acid, 4TMS; 6
-0.3
-0.2
-0.1
0.0
Coefficient
P-value
(log10)
-1.313149

5.2.3 Fully-Adjusted Model

```
## [1] "Fitting models:"
## [1] "~ E_I + Age + bmi + Blood_glucose + Duration_DM + Gender + Hba1c_baseline + log_Blood_TGA + Smo
## [1] ""
```

5.2.3.1 Tables of Model Coefficients

```
## [1] ""
## [1] "Table: E_I"
## [1] "  (from model: "
## [1] "   ~ E_I + Age + bmi + Blood_glucose + Duration_DM + Gender"
## [1] "     + Hba1c_baseline + log_Blood_TGA + Smoking + Statin +"
## [1] "     Total_cholesterol + egfr)"
## [1] ""
##                               Name Coefficient P.Value adj.P.Val
## 1            Citric acid, 4TMS; 6    -0.15900  0.0143     0.526
## 2            Tridecanoic acid; 74    -0.15900  0.0149     0.526
## 3           Linoleic acid, TMS; 4    -0.14700  0.0240     0.526
## 4             Threonine, 3TMS; 12    -0.13800  0.0339     0.526
## 5                Ribonic acid; 72    -0.13200  0.0402     0.526
## 6  2-Hydroxybutyric acid, 2TMS; 2    -0.11800  0.0674     0.526
## 7            Cholesterol, TMS; 23    -0.11300  0.0790     0.526
## 8                 Bisphenol A; 48    -0.11400  0.0827     0.526
## 9   2-hydroxy Isovaleric acid; 38    -0.11000  0.0904     0.526
## 10         Heptadecanoic acid; 61    -0.10900  0.0942     0.526
## 11          Palmitic acid, TMS; 5    -0.10700  0.0979     0.526
## 12             1-Monopalmitin; 37    -0.10700  0.1000     0.526
## 13        3-Indoleacetic acid; 40     0.10600  0.1030     0.526
## 14     4-Hydroxybutanoic acid; 43    -0.10600  0.1050     0.526
## 15      Arachidonic acid, TMS; 24    -0.10600  0.1050     0.526
## 16         Succinic acid, 2TMS; 7    -0.10200  0.1190     0.560
## 17 2,4-Dihydroxybutanoic acid; 28    -0.09640  0.1330     0.565
## 18             Oleic acid, TMS; 3    -0.09700  0.1360     0.565
## 19             Tartronic acid; 73     0.08800  0.1730     0.635
## 20                 Creatinine; 50    -0.08790  0.1750     0.635
## 21       4-Deoxytetronic acid; 32    -0.08560  0.1870     0.635
## 22           Myristoleic acid; 65    -0.08410  0.1960     0.635
## 23               1-Dodecanol; 36    -0.08240  0.2080     0.635
## 24             L-5-Oxoproline; 63    -0.08210  0.2080     0.635
## 25              Hydroxylamine; 62     0.08150  0.2120     0.635
## 26         Glutamic acid, 3TMS; 8    -0.07490  0.2480     0.691
## 27              Nonanoic acid; 67    -0.07550  0.2490     0.691
## 28         11-Eicosenoic acid; 35     0.07090  0.2770     0.741
## 29       Docosahexaenoic acid; 53     0.06850  0.2900     0.750
## 30           Stearic acid, TMS; 2    -0.06340  0.3300     0.782
## 31      Eicosapentaenoic acid; 55     0.06280  0.3320     0.782
## 32               Serine, 3TMS; 14    -0.05930  0.3610     0.782
## 33 3,4-Dihydroxybutanoic acid; 27    -0.05880  0.3610     0.782
## 34                Campesterol; 49    -0.05890  0.3640     0.782
## 35                   Glycerol; 58    -0.05900  0.3660     0.782
## 36         Glyceryl-glycoside; 59    -0.05480  0.3990     0.782
## 37           Isoleucine, 2TMS; 18    -0.05220  0.4190     0.782
## 38            Myo inositol 6TMS; 1   -0.05160  0.4210     0.782
## 39              Alanine, 2TMS; 25     0.05100  0.4330     0.782
## 40              Proline, 2TMS; 21    -0.05000  0.4410     0.782
## 41              Leucine, 2TMS; 19    -0.04950  0.4460     0.782
## 42           Methionine, 2TMS; 16     0.04930  0.4480     0.782
## 43                    Ribitol; 71     0.04610  0.4720     0.782
## 44                Pyruvic acid; 31     0.04660  0.4740     0.782
```

```
## 45                   Tyrosine; 75     0.04490  0.4900     0.782
## 46          Fumaric acid, 2TMS; 9    -0.04300  0.5100     0.782
## 47              Decanoic acid; 52    -0.04280  0.5100     0.782
## 48              Glycine, 3TMS; 17    -0.04250  0.5140     0.782
## 49               Valine, 2TMS; 20    -0.04150  0.5220     0.782
## 50       4-Deoxytetronic acid; 33     0.04120  0.5250     0.782
## 51            Arabinopyranose; 51    -0.03960  0.5380     0.782
## 52 alpha-ketoglutaric acid, TMS M     0.03970  0.5420     0.782
## 53          Aminomalonic acid; 45    -0.03810  0.5560     0.782
## 54 3-Hydroxybutyric acid, 2TMS; 1    -0.03730  0.5670     0.782
## 55     3-Indolepropionic acid; 41     0.03670  0.5740     0.782
## 56             Hydroxyproline; 64    -0.03040  0.6410     0.858
## 57         Benzeneacetic acid; 47    -0.02550  0.6960     0.873
## 58            1,3-Propanediol; 34     0.02500  0.7020     0.873
## 59 4-Hydroxyphenyllactic acid; 44    -0.02370  0.7160     0.873
## 60           Malic acid, 3TMS; 11    -0.02340  0.7200     0.873
## 61            Dodecanoic acid; 54    -0.02340  0.7200     0.873
## 62           alpha-Tocopherol; 26    -0.02210  0.7330     0.873
## 63                   Glycerol; 57    -0.02050  0.7530     0.873
## 64                    Ribitol; 70    -0.01980  0.7610     0.873
## 65         Heptadecanoic acid; 60    -0.01930  0.7670     0.873
## 66          Pyroglutamic acid; 69     0.01920  0.7680     0.873
## 67                Lactic acid; 29     0.01710  0.7930     0.888
## 68              Glyceric acid; 30    -0.01550  0.8110     0.891
## 69          Nonadecanoic acid; 66     0.01480  0.8200     0.891
## 70               Ethanolamine; 56    -0.01380  0.8320     0.892
## 71 4-Hydroxybenzeneacetic acid; 4    -0.00965  0.8810     0.925
## 72        2-Palmitoylglycerol; 39    -0.00874  0.8940     0.925
## 73              Octanoic acid; 68     0.00815  0.9000     0.925
## 74        Phenylalanine, 2TMS; 13     0.00370  0.9550     0.956
## 75             Arachidic acid; 46     0.00359  0.9560     0.956
```

5.2.3.2 Forest Plot of Model Coefficients

```
## Warning: Ignoring unknown aesthetics: x

## Warning: Ignoring unknown aesthetics: x

## Warning: Ignoring unknown aesthetics: x

## Warning: Ignoring unknown aesthetics: x

## Warning: Ignoring unknown aesthetics: x

## Warning: Ignoring unknown aesthetics: x

## Warning: Ignoring unknown aesthetics: x

## NULL
```

5.3 Lying to Standing Test (lig_staa)

5.3.1 Crude Model

```
## [1] "Fitting models:"
## [1] "~ lig_staa"
## [1] ""
```

5.3.1.1 Tables of Model Coefficients

```
## [1] ""
## [1] "Table: lig_staa"
## [1] "  (from model: "
## [1] "   ~ lig_staa)"
## [1] ""
##                                Name Coefficient  P.Value adj.P.Val
## 1  3,4-Dihydroxybutanoic acid; 27   -0.206000 0.000663    0.0394
## 2  2,4-Dihydroxybutanoic acid; 28   -0.198000 0.001050    0.0394
## 3                  Creatinine; 50   -0.155000 0.010200    0.1670
## 4            Myo inositol 6TMS; 1   -0.153000 0.011100    0.1670
## 5                Ethanolamine; 56    0.153000 0.011200    0.1670
## 6  4-Hydroxybenzeneacetic acid; 4   -0.148000 0.014400    0.1800
## 7                     Ribitol; 71   -0.117000 0.052500    0.5620
## 8       Arachidonic acid, TMS; 24   -0.109000 0.070200    0.6430
## 9                Ribonic acid; 72   -0.107000 0.077200    0.6430
## 10             Nonanoic acid; 67   -0.100000 0.097500    0.7300
## 11            L-5-Oxoproline; 63   -0.097300 0.107000    0.7300
## 12             Hydroxylamine; 62    0.088500 0.143000    0.8650
## 13                  Glycerol; 58   -0.083500 0.167000    0.8650
## 14             Octanoic acid; 68    0.080900 0.180000    0.8650
## 15          Isoleucine, 2TMS; 18    0.077100 0.202000    0.8650
## 16          Methionine, 2TMS; 16    0.076600 0.205000    0.8650
## 17             Glycine, 3TMS; 17   -0.075500 0.211000    0.8650
## 18         Pyroglutamic acid; 69   -0.071200 0.238000    0.8650
## 19                  Glycerol; 57   -0.068900 0.254000    0.8650
## 20      4-Deoxytetronic acid; 32   -0.068500 0.257000    0.8650
## 21          Myristoleic acid; 65   -0.066200 0.273000    0.8650
## 22        2-Palmitoylglycerol; 39    0.064900 0.282000    0.8650
## 23            Oleic acid, TMS; 3   -0.061000 0.313000    0.8650
## 24         Nonadecanoic acid; 66    0.060900 0.313000    0.8650
## 25            Arachidic acid; 46    0.059000 0.328000    0.8650
## 26              Valine, 2TMS; 20    0.057400 0.342000    0.8650
## 27             Glyceric acid; 30    0.056900 0.346000    0.8650
## 28        Succinic acid, 2TMS; 7   -0.056900 0.346000    0.8650
## 29 4-Hydroxyphenyllactic acid; 44   -0.055300 0.360000    0.8650
## 30         Glyceryl-glycoside; 59   -0.054700 0.365000    0.8650
## 31 alpha-ketoglutaric acid, TMS M   -0.051900 0.390000    0.8650
## 32             Hydroxyproline; 64   -0.050900 0.399000    0.8650
## 33         Fumaric acid, 2TMS; 9   -0.049900 0.409000    0.8650
## 34         Aminomalonic acid; 45   -0.047100 0.436000    0.8650
## 35        Glutamic acid, 3TMS; 8   -0.045800 0.448000    0.8650
## 36              Serine, 3TMS; 14    0.042700 0.480000    0.8650
## 37     3-Indolepropionic acid; 41    0.042600 0.481000    0.8650
## 38                   Ribitol; 70   -0.042100 0.486000    0.8650
## 39         Linoleic acid, TMS; 4    0.041400 0.493000    0.8650
## 40      4-Deoxytetronic acid; 33   -0.040400 0.503000    0.8650
## 41      Docosahexaenoic acid; 53    0.039800 0.510000    0.8650
## 42          alpha-Tocopherol; 26    0.038300 0.526000    0.8650
## 43          Citric acid, 4TMS; 6   -0.036200 0.548000    0.8650
## 44          Tridecanoic acid; 74   -0.035800 0.554000    0.8650
## 45             Leucine, 2TMS; 19    0.035700 0.554000    0.8650
## 46 3-Hydroxybutyric acid, 2TMS; 1   -0.033800 0.576000    0.8650
```

```
## 47             1-Monopalmitin; 37   -0.033400 0.580000    0.8650
## 48  2-hydroxy Isovaleric acid; 38   -0.031200 0.606000    0.8650
## 49               Proline, 2TMS; 21   -0.030500 0.614000    0.8650
## 50               Alanine, 2TMS; 25   -0.029800 0.621000    0.8650
## 51 2-Hydroxybutyric acid, 2TMS; 2    0.029300 0.628000    0.8650
## 52                 1-Dodecanol; 36   -0.028900 0.633000    0.8650
## 53                Pyruvic acid; 31   -0.028000 0.643000    0.8650
## 54           Benzeneacetic acid; 47   -0.027700 0.646000    0.8650
## 55          Phenylalanine, 2TMS; 13    0.027500 0.649000    0.8650
## 56       Eicosapentaenoic acid; 55    0.026400 0.662000    0.8650
## 57          3-Indoleacetic acid; 40   -0.026100 0.665000    0.8650
## 58               Tartronic acid; 73    0.025000 0.679000    0.8650
## 59                  Lactic acid; 29    0.024700 0.683000    0.8650
## 60                  Bisphenol A; 48    0.023900 0.692000    0.8650
## 61                     Tyrosine; 75   -0.022600 0.709000    0.8710
## 62              Dodecanoic acid; 54   -0.018900 0.755000    0.9130
## 63             Cholesterol, TMS; 23   -0.017800 0.768000    0.9150
## 64             Malic acid, 3TMS; 11    0.015200 0.801000    0.9220
## 65           Heptadecanoic acid; 60   -0.014500 0.810000    0.9220
## 66              Threonine, 3TMS; 12   -0.014200 0.813000    0.9220
## 67                  Campesterol; 49   -0.012700 0.834000    0.9220
## 68           Palmitic acid, TMS; 5    -0.012500 0.836000    0.9220
## 69                Decanoic acid; 52   -0.010700 0.859000    0.9340
## 70           11-Eicosenoic acid; 35   -0.008240 0.891000    0.9550
## 71             1,3-Propanediol; 34    0.005340 0.930000    0.9710
## 72              Arabinopyranose; 51   -0.005140 0.932000    0.9710
## 73             Stearic acid, TMS; 2    0.003060 0.960000    0.9860
## 74           Heptadecanoic acid; 61    0.001390 0.982000    0.9880
## 75       4-Hydroxybutanoic acid; 43   -0.000927 0.988000    0.9880
```

5.3.1.2 Forest Plot of Model Coefficients

```
## Warning: Ignoring unknown aesthetics: x
```

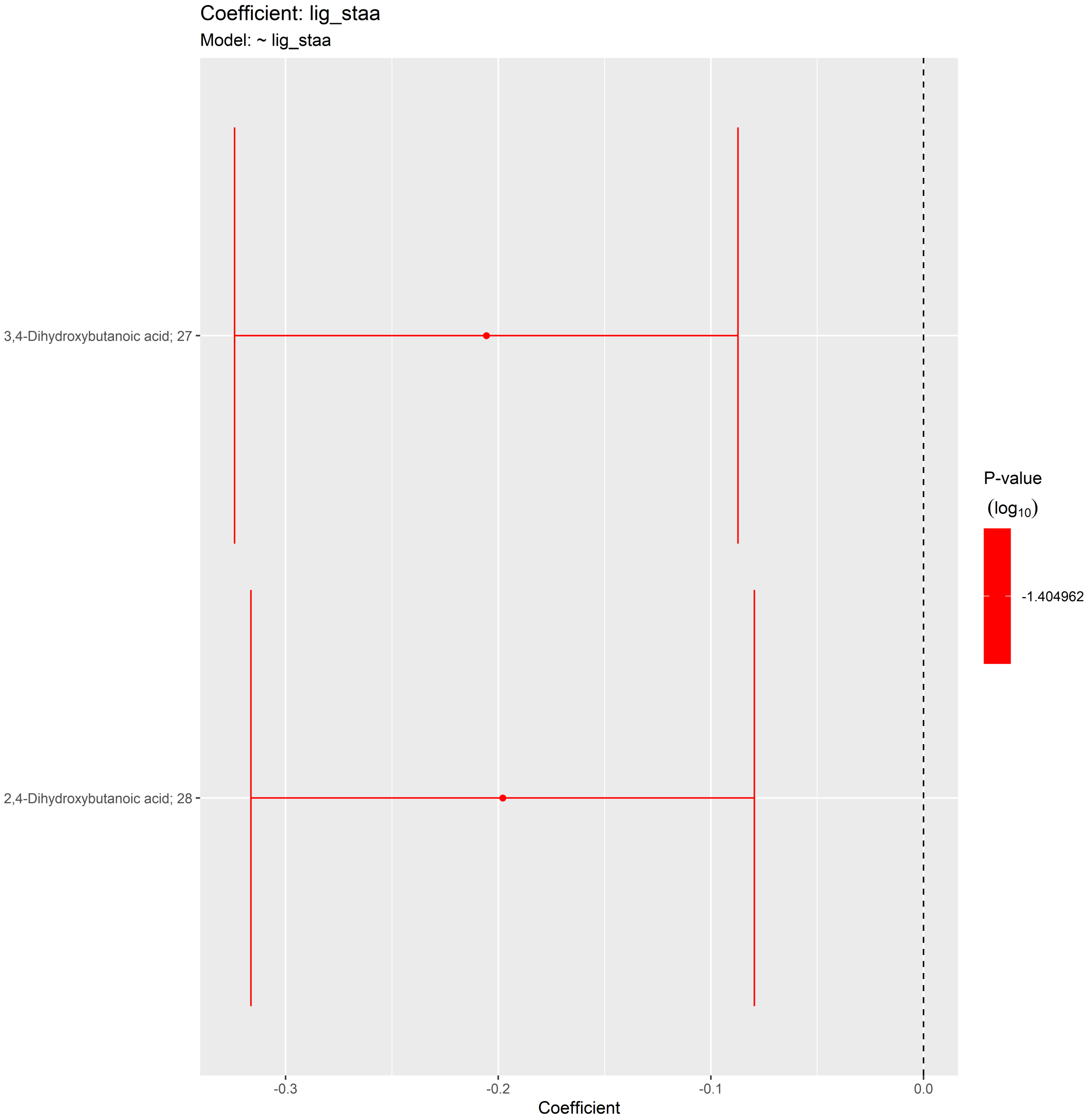

5.3.2 Adjusted Model

```
## [1] "Fitting models:"
## [1] "~ lig_staa + Age + bmi + Blood_glucose + Duration_DM + Gender + Hba1c_baseline + log_Blood_TGA +
## [1] ""
```

5.3.2.1 Tables of Model Coefficients

```
## [1] ""
## [1] "Table: lig_staa"
## [1] "  (from model: "
## [1] "   ~ lig_staa + Age + bmi + Blood_glucose + Duration_DM +"
## [1] "     Gender + Hba1c_baseline + log_Blood_TGA + Smoking + Statin +"
## [1] "     Total_cholesterol)"
## [1] ""
##                                Name Coefficient P.Value adj.P.Val
## 1  2,4-Dihydroxybutanoic acid; 28   -0.147000  0.0157     0.469
## 2                Ethanolamine; 56    0.138000  0.0231     0.469
## 3            Myo inositol 6TMS; 1   -0.135000  0.0259     0.469
## 4                  Creatinine; 50   -0.135000  0.0261     0.469
## 5               Nonanoic acid; 67   -0.131000  0.0314     0.469
## 6  3,4-Dihydroxybutanoic acid; 27   -0.126000  0.0375     0.469
## 7       Arachidonic acid, TMS; 24   -0.103000  0.0902     0.827
## 8              L-5-Oxoproline; 63   -0.101000  0.0950     0.827
## 9               Hydroxylamine; 62    0.100000  0.0992     0.827
## 10 4-Hydroxybenzeneacetic acid; 4   -0.091800  0.1310     0.980
## 11               Ribonic acid; 72   -0.084400  0.1650     0.980
## 12           Isoleucine, 2TMS; 18    0.082600  0.1740     0.980
## 13                   Glycerol; 58   -0.080500  0.1850     0.980
## 14                   Glycerol; 57   -0.076800  0.2060     0.980
## 15              Glycine, 3TMS; 17   -0.074300  0.2220     0.980
## 16           Methionine, 2TMS; 16    0.072900  0.2300     0.980
## 17        2-Palmitoylglycerol; 39    0.072700  0.2320     0.980
## 18           Nonadecanoic acid; 66    0.065900  0.2780     0.980
## 19           Cholesterol, TMS; 23   -0.063800  0.2940     0.980
## 20                    Ribitol; 71   -0.063700  0.2950     0.980
## 21              Oleic acid, TMS; 3   -0.059800  0.3250     0.980
## 22              Arachidic acid; 46    0.058300  0.3380     0.980
## 23  2-hydroxy Isovaleric acid; 38   -0.058200  0.3380     0.980
## 24              Hydroxyproline; 64   -0.057900  0.3410     0.980
## 25       4-Deoxytetronic acid; 32   -0.053000  0.3830     0.980
## 26            alpha-Tocopherol; 26    0.053000  0.3840     0.980
## 27                 1-Dodecanol; 36   -0.052800  0.3850     0.980
## 28         Succinic acid, 2TMS; 7   -0.051500  0.3970     0.980
## 29           Aminomalonic acid; 45   -0.051200  0.3990     0.980
## 30              Octanoic acid; 68    0.049600  0.4150     0.980
## 31            Threonine, 3TMS; 12   -0.045400  0.4550     0.980
## 32            Myristoleic acid; 65   -0.045300  0.4560     0.980
## 33            Tridecanoic acid; 74   -0.040200  0.5090     0.980
## 34           Pyroglutamic acid; 69   -0.038000  0.5320     0.980
## 35                 Lactic acid; 29    0.037900  0.5330     0.980
## 36        Phenylalanine, 2TMS; 13    0.033500  0.5810     0.980
## 37       Eicosapentaenoic acid; 55    0.032100  0.5970     0.980
## 38              Leucine, 2TMS; 19    0.031900  0.5990     0.980
## 39 alpha-ketoglutaric acid, TMS M   -0.030900  0.6110     0.980
## 40       Docosahexaenoic acid; 53    0.030700  0.6130     0.980
## 41         Glutamic acid, 3TMS; 8   -0.030400  0.6170     0.980
## 42              Alanine, 2TMS; 25   -0.030400  0.6170     0.980
## 43          Fumaric acid, 2TMS; 9   -0.029000  0.6340     0.980
## 44 2-Hydroxybutyric acid, 2TMS; 2    0.028600  0.6380     0.980
```

```
## 45          Citric acid, 4TMS; 6   -0.027200  0.6540     0.980
## 46 4-Hydroxyphenyllactic acid; 44   -0.027100  0.6550     0.980
## 47        Heptadecanoic acid; 60   -0.027100  0.6560     0.980
## 48        Glyceryl-glycoside; 59   -0.027000  0.6570     0.980
## 49             Proline, 2TMS; 21   -0.026800  0.6590     0.980
## 50              Valine, 2TMS; 20    0.025900  0.6700     0.980
## 51         Palmitic acid, TMS; 5   -0.025300  0.6770     0.980
## 52             Glyceric acid; 30    0.024100  0.6910     0.980
## 53      4-Deoxytetronic acid; 33   -0.022700  0.7090     0.980
## 54 3-Hydroxybutyric acid, 2TMS; 1   -0.021900  0.7190     0.980
## 55         Linoleic acid, TMS; 4    0.020400  0.7380     0.980
## 56    3-Indolepropionic acid; 41    0.019100  0.7530     0.980
## 57        Benzeneacetic acid; 47   -0.017200  0.7780     0.980
## 58            1-Monopalmitin; 37   -0.016500  0.7860     0.980
## 59              Serine, 3TMS; 14    0.015000  0.8060     0.980
## 60       3-Indoleacetic acid; 40   -0.014500  0.8110     0.980
## 61              Pyruvic acid; 31   -0.013000  0.8310     0.980
## 62           1,3-Propanediol; 34   -0.011300  0.8520     0.980
## 63          Malic acid, 3TMS; 11    0.011200  0.8530     0.980
## 64            Tartronic acid; 73    0.010500  0.8630     0.980
## 65          Stearic acid, TMS; 2    0.010400  0.8640     0.980
## 66             Decanoic acid; 52    0.007920  0.8960     0.980
## 67        Heptadecanoic acid; 61   -0.006740  0.9120     0.980
## 68           Arabinopyranose; 51   -0.006370  0.9170     0.980
## 69                   Ribitol; 70   -0.006320  0.9170     0.980
## 70    4-Hydroxybutanoic acid; 43   -0.005550  0.9270     0.980
## 71               Bisphenol A; 48    0.005540  0.9270     0.980
## 72        11-Eicosenoic acid; 35    0.002860  0.9620     0.992
## 73           Dodecanoic acid; 54   -0.001370  0.9820     0.992
## 74                  Tyrosine; 75   -0.000878  0.9880     0.992
## 75               Campesterol; 49    0.000615  0.9920     0.992
```

5.3.2.2 Forest Plot of Model Coefficients

```
## Warning: Ignoring unknown aesthetics: x

## Warning: Ignoring unknown aesthetics: x

## Warning: Ignoring unknown aesthetics: x

## Warning: Ignoring unknown aesthetics: x

## Warning: Ignoring unknown aesthetics: x

## NULL
```

5.3.3 Fully-Adjusted Model

```
## [1] "Fitting models:"
## [1] "~ lig_staa + Age + bmi + Blood_glucose + Duration_DM + Gender + Hba1c_baseline + log_Blood_TGA +
## [1] ""
```

5.3.3.1 Tables of Model Coefficients

```
## [1] ""
## [1] "Table: lig_staa"
## [1] "  (from model: "
## [1] "   ~ lig_staa + Age + bmi + Blood_glucose + Duration_DM +"
## [1] "     Gender + Hba1c_baseline + log_Blood_TGA + Smoking + Statin +"
## [1] "     Total_cholesterol + egfr)"
## [1] ""
##                              Name Coefficient P.Value adj.P.Val
## 1               Nonanoic acid; 67   -0.130000  0.0322     0.988
## 2                Ethanolamine; 56    0.128000  0.0344     0.988
## 3        Arachidonic acid, TMS; 24   -0.119000  0.0495     0.988
## 4               Hydroxylamine; 62    0.113000  0.0633     0.988
## 5              L-5-Oxoproline; 63   -0.105000  0.0840     0.988
## 6                    Glycerol; 57   -0.096900  0.1110     0.988
## 7    2-hydroxy Isovaleric acid; 38   -0.085000  0.1610     0.988
## 8            Cholesterol, TMS; 23   -0.084200  0.1620     0.988
## 9  2,4-Dihydroxybutanoic acid; 28   -0.075700  0.2080     0.988
## 10                 Creatinine; 50   -0.076100  0.2080     0.988
## 11        2-Palmitoylglycerol; 39    0.076000  0.2110     0.988
## 12             Oleic acid, TMS; 3   -0.067000  0.2680     0.988
## 13 3,4-Dihydroxybutanoic acid; 27   -0.066300  0.2710     0.988
## 14                   Glycerol; 58   -0.059100  0.3310     0.988
## 15           Myo inositol 6TMS; 1   -0.057900  0.3350     0.988
## 16          Nonadecanoic acid; 66    0.058500  0.3350     0.988
## 17            Threonine, 3TMS; 12   -0.058600  0.3350     0.988
## 18         Glutamic acid, 3TMS; 8   -0.055700  0.3570     0.988
## 19           alpha-Tocopherol; 26    0.054200  0.3700     0.988
## 20             Arachidic acid; 46    0.052000  0.3920     0.988
## 21          Aminomalonic acid; 45   -0.050900  0.4000     0.988
## 22               1-Dodecanol; 36   -0.049500  0.4150     0.988
## 23              Glycine, 3TMS; 17   -0.048700  0.4210     0.988
## 24           Myristoleic acid; 65   -0.048700  0.4210     0.988
## 25         Succinic acid, 2TMS; 7   -0.048200  0.4280     0.988
## 26           Methionine, 2TMS; 16    0.048000  0.4290     0.988
## 27           Isoleucine, 2TMS; 18    0.046900  0.4380     0.988
## 28          Palmitic acid, TMS; 5   -0.043200  0.4760     0.988
## 29        Phenylalanine, 2TMS; 13    0.038000  0.5320     0.988
## 30           Malic acid, 3TMS; 11    0.037700  0.5350     0.988
## 31           Tridecanoic acid; 74   -0.037000  0.5420     0.988
## 32 4-Hydroxybenzeneacetic acid; 4   -0.035400  0.5580     0.988
## 33                   Tyrosine; 75   -0.030900  0.6110     0.988
## 34 alpha-ketoglutaric acid, TMS M   -0.030900  0.6110     0.988
## 35       4-Deoxytetronic acid; 33    0.030400  0.6150     0.988
## 36          Linoleic acid, TMS; 4    0.029500  0.6270     0.988
## 37         Heptadecanoic acid; 60   -0.027300  0.6530     0.988
## 38              Alanine, 2TMS; 25   -0.027200  0.6540     0.988
## 39                Lactic acid; 29    0.026500  0.6630     0.988
## 40               Ribonic acid; 72   -0.024200  0.6880     0.988
## 41     3-Indolepropionic acid; 41    0.021800  0.7190     0.988
## 42               Serine, 3TMS; 14   -0.021400  0.7230     0.988
## 43        3-Indoleacetic acid; 40    0.021200  0.7260     0.988
## 44              Octanoic acid; 68    0.021200  0.7270     0.988
```

```
## 45             Proline, 2TMS; 21   -0.019900  0.7430     0.988
## 46           Citric acid, 4TMS; 6    0.019300  0.7500     0.988
## 47 3-Hydroxybutyric acid, 2TMS; 1   -0.019100  0.7530     0.988
## 48              Pyruvic acid; 31   -0.018400  0.7610     0.988
## 49      Eicosapentaenoic acid; 55    0.018100  0.7650     0.988
## 50             Hydroxyproline; 64   -0.017700  0.7700     0.988
## 51       Docosahexaenoic acid; 53    0.017400  0.7730     0.988
## 52 2-Hydroxybutyric acid, 2TMS; 2   -0.016700  0.7820     0.988
## 53                    Ribitol; 71    0.015300  0.7980     0.988
## 54         Heptadecanoic acid; 61   -0.015100  0.8040     0.988
## 55         Glyceryl-glycoside; 59    0.013900  0.8190     0.988
## 56           Stearic acid, TMS; 2   -0.012300  0.8400     0.988
## 57            Dodecanoic acid; 54   -0.011700  0.8470     0.988
## 58         Benzeneacetic acid; 47   -0.011300  0.8520     0.988
## 59             Tartronic acid; 73    0.009970  0.8690     0.988
## 60                    Ribitol; 70    0.010000  0.8690     0.988
## 61              Valine, 2TMS; 20   -0.008790  0.8850     0.988
## 62             Leucine, 2TMS; 19    0.008770  0.8850     0.988
## 63             1-Monopalmitin; 37   -0.008760  0.8850     0.988
## 64            1,3-Propanediol; 34   -0.008710  0.8860     0.988
## 65           Pyroglutamic acid; 69   -0.007380  0.9030     0.988
## 66         11-Eicosenoic acid; 35    0.006590  0.9140     0.988
## 67 4-Hydroxyphenyllactic acid; 44    0.005900  0.9230     0.988
## 68          Fumaric acid, 2TMS; 9   -0.005700  0.9250     0.988
## 69                Bisphenol A; 48    0.004570  0.9400     0.988
## 70     4-Hydroxybutanoic acid; 43    0.003780  0.9500     0.988
## 71              Glyceric acid; 30    0.003320  0.9560     0.988
## 72                Campesterol; 49    0.003290  0.9570     0.988
## 73            Arabinopyranose; 51    0.002010  0.9730     0.988
## 74       4-Deoxytetronic acid; 32   -0.001920  0.9750     0.988
## 75              Decanoic acid; 52    0.000269  0.9960     0.996
```

5.3.3.2 Forest Plot of Model Coefficients

```
## Warning: Ignoring unknown aesthetics: x

## Warning: Ignoring unknown aesthetics: x

## Warning: Ignoring unknown aesthetics: x

## Warning: Ignoring unknown aesthetics: x

## Warning: Ignoring unknown aesthetics: x

## Warning: Ignoring unknown aesthetics: x

## NULL
```

5.4 Valsalva Maneuver (Valsal)

5.4.1 Crude Model

```
## [1] "Fitting models:"
## [1] "~ Valsal"
## [1] ""
```

5.4.1.1 Tables of Model Coefficients

```
## [1] ""
## [1] "Table: Valsal"
## [1] "  (from model: "
## [1] "    ~ Valsal)"
## [1] ""
##                               Name Coefficient  P.Value adj.P.Val
## 1  3,4-Dihydroxybutanoic acid; 27   -3.05e-01 2.51e-05   0.00188
## 2  2,4-Dihydroxybutanoic acid; 28   -2.74e-01 1.59e-04   0.00417
## 3  4-Hydroxybenzeneacetic acid; 4   -2.73e-01 1.67e-04   0.00417
## 4                    Glycerol; 58   -2.37e-01 1.06e-03   0.01790
## 5                     Ribitol; 70   -2.35e-01 1.20e-03   0.01790
## 6                     Ribitol; 71   -2.29e-01 1.54e-03   0.01930
## 7            Citric acid, 4TMS; 6   -2.20e-01 2.43e-03   0.02610
## 8            Myo inositol 6TMS; 1   -2.14e-01 3.16e-03   0.02960
## 9                Ribonic acid; 72   -2.04e-01 4.90e-03   0.04080
## 10               Valine, 2TMS; 20    1.74e-01 1.63e-02   0.12200
## 11       4-Deoxytetronic acid; 32   -1.71e-01 1.84e-02   0.12600
## 12          Fumaric acid, 2TMS; 9   -1.68e-01 2.02e-02   0.12600
## 13             Oleic acid, TMS; 3   -1.57e-01 3.05e-02   0.17200
## 14               Ethanolamine; 56    1.55e-01 3.21e-02   0.17200
## 15             Tartronic acid; 73    1.34e-01 6.51e-02   0.32100
## 16              Glyceric acid; 30    1.31e-01 7.15e-02   0.32100
## 17                 Creatinine; 50   -1.30e-01 7.31e-02   0.32100
## 18           Methionine, 2TMS; 16    1.27e-01 8.06e-02   0.32100
## 19         Benzeneacetic acid; 47   -1.25e-01 8.40e-02   0.32100
## 20              Leucine, 2TMS; 19    1.24e-01 8.69e-02   0.32100
## 21           Isoleucine, 2TMS; 18    1.23e-01 8.99e-02   0.32100
## 22              Alanine, 2TMS; 25    1.07e-01 1.41e-01   0.48200
## 23             Hydroxyproline; 64   -1.05e-01 1.48e-01   0.48400
## 24     3-Indolepropionic acid; 41    1.01e-01 1.64e-01   0.51100
## 25               Serine, 3TMS; 14    9.81e-02 1.76e-01   0.52000
## 26      Eicosapentaenoic acid; 55   -9.71e-02 1.80e-01   0.52000
## 27  2-hydroxy Isovaleric acid; 38    8.26e-02 2.55e-01   0.64100
## 28 4-Hydroxyphenyllactic acid; 44   -8.21e-02 2.57e-01   0.64100
## 29             1,3-Propanediol; 34    8.12e-02 2.62e-01   0.64100
## 30         Glyceryl-glycoside; 59   -8.06e-02 2.66e-01   0.64100
## 31         Succinic acid, 2TMS; 7   -7.98e-02 2.71e-01   0.64100
## 32              Glycine, 3TMS; 17   -7.82e-02 2.81e-01   0.64100
## 33           Cholesterol, TMS; 23    7.66e-02 2.90e-01   0.64100
## 34             Threonine, 3TMS; 12    7.66e-02 2.90e-01   0.64100
## 35         11-Eicosenoic acid; 35   -7.53e-02 2.99e-01   0.64100
## 36           Pyroglutamic acid; 69   -7.37e-02 3.09e-01   0.64300
## 37            Dodecanoic acid; 54   -6.78e-02 3.49e-01   0.69600
## 38                   Glycerol; 57   -6.68e-02 3.57e-01   0.69600
## 39           Myristoleic acid; 65   -6.61e-02 3.62e-01   0.69600
## 40      Arachidonic acid, TMS; 24   -6.21e-02 3.92e-01   0.73400
## 41        Phenylalanine, 2TMS; 13    6.02e-02 4.06e-01   0.74300
## 42              L-5-Oxoproline; 63   -5.61e-02 4.39e-01   0.77700
## 43              Octanoic acid; 68    5.42e-02 4.54e-01   0.77700
## 44 3-Hydroxybutyric acid, 2TMS; 1   -5.38e-02 4.57e-01   0.77700
## 45              1-Monopalmitin; 37   -5.23e-02 4.70e-01   0.77700
## 46              Arachidic acid; 46    5.05e-02 4.86e-01   0.77700
```

```
## 47           Malic acid, 3TMS; 11   -5.03e-02 4.87e-01   0.77700
## 48           alpha-Tocopherol; 26    4.90e-02 4.99e-01   0.77900
## 49              Nonanoic acid; 67    4.76e-02 5.12e-01   0.78300
## 50              Decanoic acid; 52   -4.53e-02 5.32e-01   0.79800
## 51         Heptadecanoic acid; 61   -4.28e-02 5.55e-01   0.80900
## 52                Campesterol; 49   -4.22e-02 5.61e-01   0.80900
## 53           Tridecanoic acid; 74   -3.70e-02 6.10e-01   0.85700
## 54                Lactic acid; 29    3.53e-02 6.26e-01   0.85700
## 55        2-Palmitoylglycerol; 39   -3.50e-02 6.29e-01   0.85700
## 56          Nonadecanoic acid; 66   -3.27e-02 6.52e-01   0.87300
## 57              Hydroxylamine; 62    2.95e-02 6.84e-01   0.89900
## 58               Pyruvic acid; 31    2.76e-02 7.03e-01   0.89900
## 59                Bisphenol A; 48    2.66e-02 7.14e-01   0.89900
## 60         Glutamic acid, 3TMS; 8   -2.49e-02 7.31e-01   0.89900
## 61          Aminomalonic acid; 45    2.42e-02 7.39e-01   0.89900
## 62       4-Deoxytetronic acid; 33   -2.37e-02 7.44e-01   0.89900
## 63    4-Hydroxybutanoic acid; 43   -1.91e-02 7.93e-01   0.93900
## 64 alpha-ketoglutaric acid, TMS M   -1.79e-02 8.05e-01   0.93900
## 65            Arabinopyranose; 51   -1.62e-02 8.23e-01   0.93900
## 66                1-Dodecanol; 36    1.59e-02 8.26e-01   0.93900
## 67              Proline, 2TMS; 21    1.24e-02 8.64e-01   0.96800
## 68      Docosahexaenoic acid; 53    9.69e-03 8.94e-01   0.98600
## 69          Linoleic acid, TMS; 4   -6.11e-03 9.33e-01   1.00000
## 70         Heptadecanoic acid; 60   -5.25e-03 9.42e-01   1.00000
## 71          Palmitic acid, TMS; 5    2.92e-03 9.68e-01   1.00000
## 72 2-Hydroxybutyric acid, 2TMS; 2   -8.25e-04 9.91e-01   1.00000
## 73        3-Indoleacetic acid; 40   -7.72e-04 9.91e-01   1.00000
## 74           Stearic acid, TMS; 2    2.88e-04 9.97e-01   1.00000
## 75                  Tyrosine; 75   -3.16e-05 1.00e+00   1.00000
```

5.4.1.2 Forest Plot of Model Coefficients

```
## Warning: Ignoring unknown aesthetics: x
```

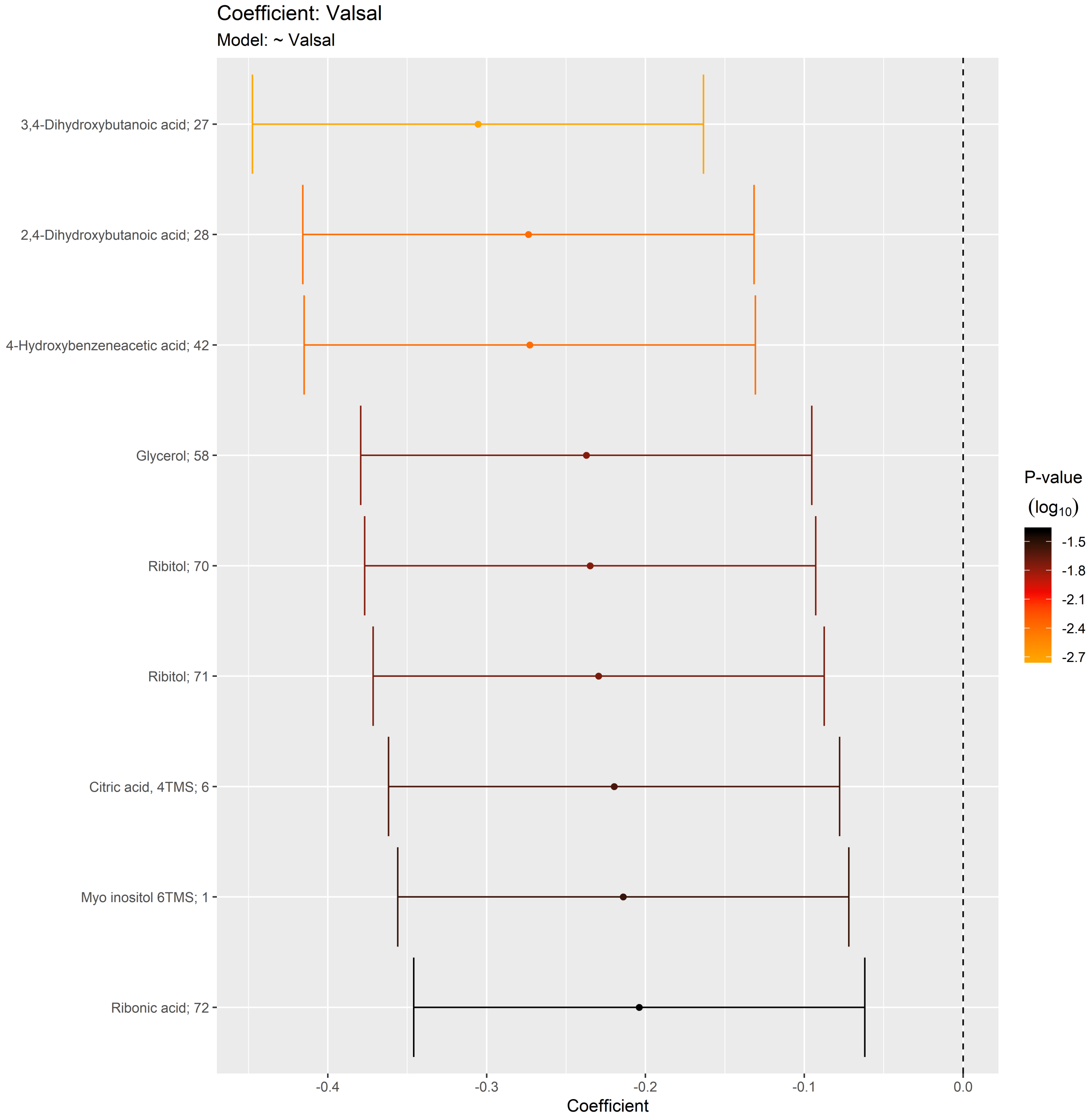

5.4.2 Adjusted Model

```
## [1] "Fitting models:"
## [1] "~ Valsal + Age + bmi + Blood_glucose + Duration_DM + Gender + Hba1c_baseline + log_Blood_TGA + [illegible]
## [1] ""
```

5.4.2.1 Tables of Model Coefficients

```
## [1] ""
## [1] "Table: Valsal"
## [1] "  (from model: "
## [1] "   ~ Valsal + Age + bmi + Blood_glucose + Duration_DM + Gender"
## [1] "     + Hba1c_baseline + log_Blood_TGA + Smoking + Statin +"
## [1] "     Total_cholesterol)"
## [1] ""
##                              Name Coefficient P.Value adj.P.Val
## 1  4-Hydroxybenzeneacetic acid; 4    -0.23400 0.00308    0.0779
## 2                    Glycerol; 58    -0.22900 0.00379    0.0779
## 3                     Ribitol; 70    -0.22700 0.00408    0.0779
## 4            Citric acid, 4TMS; 6    -0.22700 0.00416    0.0779
## 5                Ribonic acid; 72    -0.21700 0.00619    0.0929
## 6  2,4-Dihydroxybutanoic acid; 28    -0.21100 0.00775    0.0969
## 7  3,4-Dihydroxybutanoic acid; 27    -0.18900 0.01720    0.1650
## 8            Myo inositol 6TMS; 1    -0.18800 0.01760    0.1650
## 9              Oleic acid, TMS; 3    -0.16300 0.03880    0.3210
## 10                    Ribitol; 71    -0.15600 0.04840    0.3210
## 11           Isoleucine, 2TMS; 18     0.15200 0.05450    0.3210
## 12             Tartronic acid; 73     0.14800 0.06100    0.3210
## 13               Valine, 2TMS; 20     0.14700 0.06220    0.3210
## 14          Fumaric acid, 2TMS; 9    -0.14700 0.06340    0.3210
## 15              Leucine, 2TMS; 19     0.14600 0.06480    0.3210
## 16         Benzeneacetic acid; 47    -0.14400 0.06850    0.3210
## 17                   Glycerol; 57    -0.14100 0.07560    0.3330
## 18           alpha-Tocopherol; 26     0.13200 0.09510    0.3960
## 19           Methionine, 2TMS; 16     0.12700 0.10800    0.4020
## 20               Ethanolamine; 56     0.12600 0.11100    0.4020
## 21              Alanine, 2TMS; 25     0.12500 0.11500    0.4020
## 22              Glycine, 3TMS; 17    -0.12400 0.11800    0.4020
## 23                 Creatinine; 50    -0.11500 0.14800    0.4790
## 24             Hydroxyproline; 64    -0.11300 0.15300    0.4790
## 25         Succinic acid, 2TMS; 7    -0.10300 0.19200    0.5760
## 26              Glyceric acid; 30     0.09940 0.20900    0.6020
## 27               Serine, 3TMS; 14     0.07750 0.32700    0.8750
## 28     3-Indolepropionic acid; 41     0.07440 0.34700    0.8750
## 29           Tridecanoic acid; 74    -0.07310 0.35500    0.8750
## 30       4-Deoxytetronic acid; 32    -0.07250 0.36000    0.8750
## 31            Threonine, 3TMS; 12     0.07050 0.37300    0.8750
## 32      Arachidonic acid, TMS; 24    -0.07040 0.37300    0.8750
## 33         11-Eicosenoic acid; 35    -0.06700 0.39700    0.9010
## 34             Arachidic acid; 46     0.06430 0.41600    0.9010
## 35           Malic acid, 3TMS; 11    -0.06140 0.43800    0.9010
## 36             1-Monopalmitin; 37    -0.06130 0.43800    0.9010
## 37            1,3-Propanediol; 34     0.05930 0.45400    0.9010
## 38               Pyruvic acid; 31     0.05630 0.47700    0.9010
## 39              Hydroxylamine; 62     0.05290 0.50300    0.9010
## 40      Eicosapentaenoic acid; 55    -0.05270 0.50500    0.9010
## 41     4-Hydroxybutanoic acid; 43    -0.05040 0.52400    0.9010
## 42  2-hydroxy Isovaleric acid; 38     0.05000 0.52800    0.9010
## 43         Heptadecanoic acid; 61    -0.04780 0.54500    0.9010
## 44            Arabinopyranose; 51    -0.04760 0.54700    0.9010
```

```
## 45          3-Indoleacetic acid; 40    0.04650 0.55600    0.9010
## 46 4-Hydroxyphenyllactic acid; 44   -0.04500 0.57000    0.9010
## 47             L-5-Oxoproline; 63   -0.04430 0.57600    0.9010
## 48           Stearic acid, TMS; 2    0.04280 0.58900    0.9010
## 49                Campesterol; 49   -0.04270 0.58900    0.9010
## 50          Linoleic acid, TMS; 4   -0.03990 0.61400    0.9100
## 51                 1-Dodecanol; 36   -0.03930 0.61900    0.9100
## 52            Myristoleic acid; 65   -0.03740 0.63600    0.9180
## 53                   Tyrosine; 75    0.03390 0.66800    0.9300
## 54             Dodecanoic acid; 54   -0.03380 0.67000    0.9300
## 55          Heptadecanoic acid; 60   -0.03070 0.69800    0.9480
## 56        Docosahexaenoic acid; 53    0.02890 0.71500    0.9480
## 57         Phenylalanine, 2TMS; 13    0.02570 0.74500    0.9480
## 58              Proline, 2TMS; 21    0.02460 0.75600    0.9480
## 59              Nonanoic acid; 67    0.02440 0.75700    0.9480
## 60           Cholesterol, TMS; 23    0.02320 0.76900    0.9480
## 61 2-Hydroxybutyric acid, 2TMS; 2   -0.02300 0.77100    0.9480
## 62          Nonadecanoic acid; 66   -0.02030 0.79800    0.9540
## 63          Aminomalonic acid; 45   -0.01910 0.80900    0.9540
## 64 alpha-ketoglutaric acid, TMS M    0.01840 0.81600    0.9540
## 65          Pyroglutamic acid; 69   -0.01600 0.84000    0.9540
## 66                Bisphenol A; 48   -0.01400 0.85900    0.9540
## 67              Octanoic acid; 68    0.01340 0.86600    0.9540
## 68         Glyceryl-glycoside; 59   -0.01340 0.86600    0.9540
## 69              Decanoic acid; 52    0.01080 0.89200    0.9540
## 70        2-Palmitoylglycerol; 39   -0.00955 0.90400    0.9540
## 71 3-Hydroxybutyric acid, 2TMS; 1    0.00888 0.91100    0.9540
## 72          Palmitic acid, TMS; 5   -0.00837 0.91600    0.9540
## 73                Lactic acid; 29   -0.00464 0.95300    0.9730
## 74         Glutamic acid, 3TMS; 8    0.00392 0.96100    0.9730
## 75       4-Deoxytetronic acid; 33   -0.00199 0.98000    0.9800
```

5.4.2.2 Forest Plot of Model Coefficients

```
## Warning: Ignoring unknown aesthetics: x

## Warning: Ignoring unknown aesthetics: x

## Warning: Ignoring unknown aesthetics: x

## Warning: Ignoring unknown aesthetics: x

## Warning: Ignoring unknown aesthetics: x

## NULL
```

5.4.3 Fully-Adjusted Model

```
## [1] "Fitting models:"
## [1] "~ Valsal + Age + bmi + Blood_glucose + Duration_DM + Gender + Hba1c_baseline + log_Blood_TGA + S
## [1] ""
```

5.4.3.1 Tables of Model Coefficients

```
## [1] ""
## [1] "Table: Valsal"
## [1] "  (from model: "
## [1] "   ~ Valsal + Age + bmi + Blood_glucose + Duration_DM + Gender"
## [1] "     + Hba1c_baseline + log_Blood_TGA + Smoking + Statin +"
## [1] "     Total_cholesterol + egfr)"
## [1] ""
##                               Name Coefficient P.Value adj.P.Val
## 1                     Glycerol; 58   -2.23e-01 0.00548     0.219
## 2                      Ribitol; 70   -2.22e-01 0.00585     0.219
## 3               Oleic acid, TMS; 3   -1.94e-01 0.01590     0.398
## 4                     Glycerol; 57   -1.74e-01 0.03000     0.550
## 5             Citric acid, 4TMS; 6   -1.67e-01 0.03670     0.550
## 6               Tartronic acid; 73    1.56e-01 0.05070     0.580
## 7             alpha-Tocopherol; 26    1.53e-01 0.05570     0.580
## 8  4-Hydroxybenzeneacetic acid; 4   -1.49e-01 0.06180     0.580
## 9          Benzeneacetic acid; 47   -1.31e-01 0.10400     0.865
## 10              Alanine, 2TMS; 25    1.24e-01 0.12200     0.880
## 11        3-Indoleacetic acid; 40    1.18e-01 0.14300     0.880
## 12          Fumaric acid, 2TMS; 9   -1.17e-01 0.14400     0.880
## 13               Ethanolamine; 56    1.13e-01 0.15900     0.880
## 14 2,4-Dihydroxybutanoic acid; 28   -1.06e-01 0.18400     0.880
## 15               Ribonic acid; 72   -1.05e-01 0.18900     0.880
## 16 3,4-Dihydroxybutanoic acid; 27   -1.03e-01 0.19700     0.880
## 17 2-Hydroxybutyric acid, 2TMS; 2   -1.01e-01 0.20800     0.880
## 18         Succinic acid, 2TMS; 7   -9.82e-02 0.22200     0.880
## 19              Leucine, 2TMS; 19    9.66e-02 0.22800     0.880
## 20      Arachidonic acid, TMS; 24   -9.53e-02 0.23500     0.880
## 21               Glycine, 3TMS; 17   -8.34e-02 0.29800     0.894
## 22           Isoleucine, 2TMS; 18    8.23e-02 0.30400     0.894
## 23                Valine, 2TMS; 20    8.15e-02 0.30800     0.894
## 24         11-Eicosenoic acid; 35   -7.98e-02 0.32000     0.894
## 25      Eicosapentaenoic acid; 55   -7.55e-02 0.34600     0.894
## 26     3-Indolepropionic acid; 41    7.48e-02 0.35200     0.894
## 27           Myo inositol 6TMS; 1   -7.39e-02 0.35400     0.894
## 28       4-Deoxytetronic acid; 33    7.26e-02 0.36500     0.894
## 29         Heptadecanoic acid; 61   -7.15e-02 0.37300     0.894
## 30           Methionine, 2TMS; 16    7.12e-02 0.37400     0.894
## 31              L-5-Oxoproline; 63   -6.78e-02 0.39900     0.894
## 32               Glyceric acid; 30    6.58e-02 0.41100     0.894
## 33             1,3-Propanediol; 34    6.57e-02 0.41300     0.894
## 34          Linoleic acid, TMS; 4   -5.98e-02 0.45700     0.894
## 35              Hydroxyproline; 64   -5.95e-02 0.45900     0.894
## 36             Dodecanoic acid; 54   -5.81e-02 0.47000     0.894
## 37            Myristoleic acid; 65   -5.55e-02 0.48900     0.894
## 38            Tridecanoic acid; 74   -5.50e-02 0.49400     0.894
## 39                 Campesterol; 49   -5.48e-02 0.49400     0.894
## 40                Hydroxylamine; 62    5.37e-02 0.50400     0.894
## 41                 Pyruvic acid; 31    5.29e-02 0.51000     0.894
## 42              1-Monopalmitin; 37   -5.29e-02 0.51000     0.894
## 43               Octanoic acid; 68   -5.21e-02 0.51600     0.894
## 44          Nonadecanoic acid; 66   -5.11e-02 0.52500     0.894
```

```
## 45          Palmitic acid, TMS; 5   -4.83e-02 0.54700     0.895
## 46     4-Hydroxybutanoic acid; 43   -4.82e-02 0.54900     0.895
## 47            Arachidic acid; 46    4.58e-02 0.56900     0.907
## 48                   Ribitol; 71   -4.28e-02 0.59200     0.913
## 49         Glyceryl-glycoside; 59    4.25e-02 0.59600     0.913
## 50           Threonine, 3TMS; 12    3.87e-02 0.63000     0.945
## 51               1-Dodecanol; 36   -3.67e-02 0.64800     0.953
## 52           Arabinopyranose; 51   -3.48e-02 0.66300     0.957
## 53                Creatinine; 50   -3.06e-02 0.70200     0.962
## 54         Glutamic acid, 3TMS; 8   -2.96e-02 0.71100     0.962
## 55         Heptadecanoic acid; 60   -2.93e-02 0.71500     0.962
## 56          Pyroglutamic acid; 69    2.84e-02 0.72400     0.962
## 57               Bisphenol A; 48   -2.34e-02 0.77200     0.962
## 58             Proline, 2TMS; 21    2.31e-02 0.77300     0.962
## 59                  Tyrosine; 75   -2.15e-02 0.78900     0.962
## 60               Lactic acid; 29   -2.12e-02 0.79200     0.962
## 61             Nonanoic acid; 67    2.09e-02 0.79500     0.962
## 62           Malic acid, 3TMS; 11   -2.08e-02 0.79600     0.962
## 63 4-Hydroxyphenyllactic acid; 44   -1.68e-02 0.83400     0.990
## 64 alpha-ketoglutaric acid, TMS M    1.52e-02 0.85000     0.990
## 65        Phenylalanine, 2TMS; 13    9.47e-03 0.90600     0.990
## 66       Docosahexaenoic acid; 53    9.11e-03 0.90900     0.990
## 67  2-hydroxy Isovaleric acid; 38    8.38e-03 0.91700     0.990
## 68        2-Palmitoylglycerol; 39   -6.68e-03 0.93400     0.990
## 69          Aminomalonic acid; 45   -4.64e-03 0.95400     0.990
## 70       4-Deoxytetronic acid; 32    4.61e-03 0.95400     0.990
## 71           Stearic acid, TMS; 2   -3.03e-03 0.97000     0.990
## 72 3-Hydroxybutyric acid, 2TMS; 1    2.76e-03 0.97300     0.990
## 73              Serine, 3TMS; 14    2.65e-03 0.97400     0.990
## 74           Cholesterol, TMS; 23   -2.35e-03 0.97700     0.990
## 75             Decanoic acid; 52   -1.57e-05 1.00000     1.000
```

5.4.3.2 Forest Plot of Model Coefficients

```
## Warning: Ignoring unknown aesthetics: x

## Warning: Ignoring unknown aesthetics: x

## Warning: Ignoring unknown aesthetics: x

## Warning: Ignoring unknown aesthetics: x

## Warning: Ignoring unknown aesthetics: x

## NULL
```

5.5 Heart Rate Variablity (SDNN)

5.5.1 Crude Model

```
## [1] "Fitting models:"
## [1] "~ SDNN + rest_HR_vag"
## [1] ""
```

5.5.1.1 Tables of Model Coefficients

```
## [1] ""
## [1] "Table: SDNN"
## [1] "  (from model: "
## [1] "    ~ SDNN + rest_HR_vag)"
## [1] ""
##                                Name Coefficient P.Value adj.P.Val
## 1         4-Deoxytetronic acid; 32    -0.17900 0.00562     0.243
## 2             Myo inositol 6TMS; 1    -0.16600 0.01030     0.243
## 3  2,4-Dihydroxybutanoic acid; 28    -0.15900 0.01410     0.243
## 4  3,4-Dihydroxybutanoic acid; 27    -0.15700 0.01530     0.243
## 5                Valine, 2TMS; 20    -0.15500 0.01620     0.243
## 6                  Creatinine; 50    -0.14400 0.02590     0.322
## 7               Leucine, 2TMS; 19    -0.14000 0.03010     0.322
## 8             Arabinopyranose; 51    -0.13100 0.04300     0.403
## 9                 Lactic acid; 29     0.12200 0.05870     0.448
## 10          Tridecanoic acid; 74    -0.12200 0.05980     0.448
## 11                   Ribitol; 70    -0.11800 0.06720     0.458
## 12              Ribonic acid; 72    -0.11300 0.08140     0.474
## 13            Threonine, 3TMS; 12    -0.11200 0.08430     0.474
## 14        Phenylalanine, 2TMS; 13    -0.11000 0.08840     0.474
## 15          Isoleucine, 2TMS; 18    -0.09890 0.12600     0.629
## 16            Dodecanoic acid; 54     0.09200 0.15400     0.638
## 17          Glutamic acid, 3TMS; 8    -0.08810 0.17200     0.638
## 18              Octanoic acid; 68     0.08510 0.18800     0.638
## 19 4-Hydroxybenzeneacetic acid; 4    -0.08450 0.19100     0.638
## 20        3-Indoleacetic acid; 40    -0.08430 0.19200     0.638
## 21             L-5-Oxoproline; 63    -0.08280 0.20000     0.638
## 22              Nonanoic acid; 67    -0.08260 0.20100     0.638
## 23             Oleic acid, TMS; 3    -0.08200 0.20400     0.638
## 24     3-Indolepropionic acid; 41     0.07970 0.21700     0.638
## 25              Proline, 2TMS; 21    -0.07700 0.23300     0.638
## 26          Glyceryl-glycoside; 59    -0.07690 0.23400     0.638
## 27             Arachidic acid; 46     0.07590 0.24000     0.638
## 28           Citric acid, 4TMS; 6    -0.07500 0.24600     0.638
## 29        2-Palmitoylglycerol; 39     0.07480 0.24700     0.638
## 30              Glycine, 3TMS; 17    -0.06700 0.30000     0.730
## 31             Hydroxyproline; 64    -0.06550 0.31000     0.730
## 32                    Ribitol; 71    -0.06460 0.31700     0.730
## 33               Serine, 3TMS; 14    -0.06160 0.34100     0.730
## 34 3-Hydroxybutyric acid, 2TMS; 1    -0.05840 0.36600     0.730
## 35 2-Hydroxybutyric acid, 2TMS; 2    -0.05830 0.36700     0.730
## 36                   Glycerol; 57    -0.05800 0.36900     0.730
## 37     4-Hydroxybutanoic acid; 43    -0.05780 0.37100     0.730
## 38         Benzeneacetic acid; 47    -0.05680 0.37900     0.730
## 39           alpha-Tocopherol; 26    -0.05680 0.37900     0.730
## 40           Pyroglutamic acid; 69    -0.05550 0.39100     0.732
## 41           Methionine, 2TMS; 16    -0.05390 0.40400     0.740
## 42           Myristoleic acid; 65    -0.05110 0.42900     0.758
## 43      Arachidonic acid, TMS; 24    -0.05010 0.43800     0.758
## 44                 Bisphenol A; 48    -0.04860 0.45200     0.758
## 45           Malic acid, 3TMS; 11     0.04830 0.45500     0.758
## 46              Hydroxylamine; 62     0.04470 0.48900     0.797
```

```
## 47                  Tyrosine; 75    -0.04230 0.51200     0.817
## 48             1-Monopalmitin; 37    -0.04070 0.52800     0.825
## 49              Glyceric acid; 30     0.03800 0.55600     0.851
## 50         Heptadecanoic acid; 60     0.03660 0.57100     0.856
## 51  2-hydroxy Isovaleric acid; 38    -0.03260 0.61400     0.860
## 52              Decanoic acid; 52     0.03250 0.61500     0.860
## 53           Fumaric acid, 2TMS; 9    -0.02940 0.64900     0.860
## 54           Palmitic acid, TMS; 5    -0.02930 0.65000     0.860
## 55               Ethanolamine; 56     0.02900 0.65300     0.860
## 56              Alanine, 2TMS; 25    -0.02760 0.66900     0.860
## 57           Aminomalonic acid; 45    -0.02670 0.67900     0.860
## 58       4-Deoxytetronic acid; 33     0.02620 0.68500     0.860
## 59           Linoleic acid, TMS; 4    -0.02600 0.68700     0.860
## 60      Eicosapentaenoic acid; 55    -0.02490 0.70000     0.860
## 61            Tartronic acid; 73     0.02260 0.72700     0.860
## 62                Campesterol; 49    -0.02180 0.73600     0.860
## 63               Pyruvic acid; 31    -0.02120 0.74200     0.860
## 64         Heptadecanoic acid; 61    -0.02080 0.74700     0.860
## 65            1,3-Propanediol; 34     0.02060 0.75000     0.860
## 66                   Glycerol; 58    -0.02000 0.75700     0.860
## 67 alpha-ketoglutaric acid, TMS M     0.01710 0.79100     0.886
## 68 4-Hydroxyphenyllactic acid; 44     0.01550 0.81000     0.894
## 69          Nonadecanoic acid; 66    -0.01330 0.83700     0.896
## 70            Cholesterol, TMS; 23    -0.01280 0.84200     0.896
## 71         Succinic acid, 2TMS; 7     0.01240 0.84800     0.896
## 72            Stearic acid, TMS; 2     0.00640 0.92100     0.959
## 73         11-Eicosenoic acid; 35     0.00458 0.94300     0.965
## 74                1-Dodecanol; 36    -0.00305 0.96200     0.965
## 75      Docosahexaenoic acid; 53     0.00281 0.96500     0.965
```

5.5.1.2 Forest Plot of Model Coefficients

```
## Warning: Ignoring unknown aesthetics: x
```

```
## NULL
```

5.5.2 Adjusted Model

```
## [1] "Fitting models:"
## [1] "~ SDNN + rest_HR_vag + Age + bmi + Blood_glucose + Duration_DM + Gender + Hba1c_baseline + log_
## [1] ""
```

5.5.2.1 Tables of Model Coefficients

```
## [1] ""
## [1] "Table: SDNN"
## [1] "  (from model: "
## [1] "   ~ SDNN + rest_HR_vag + Age + bmi + Blood_glucose +"
## [1] "     Duration_DM + Gender + Hba1c_baseline + log_Blood_TGA +"
## [1] "     Smoking + Statin + Total_cholesterol)"
## [1] ""
##                               Name Coefficient P.Value adj.P.Val
## 1                Valine, 2TMS; 20    -0.18100 0.00636     0.477
## 2        4-Deoxytetronic acid; 32    -0.15600 0.01830     0.547
## 3             Threonine, 3TMS; 12    -0.13500 0.04080     0.547
## 4               Leucine, 2TMS; 19    -0.13200 0.04540     0.547
## 5         Phenylalanine, 2TMS; 13    -0.13000 0.05010     0.547
## 6            Tridecanoic acid; 74    -0.12800 0.05320     0.547
## 7                  Creatinine; 50    -0.12400 0.06130     0.547
## 8  2,4-Dihydroxybutanoic acid; 28    -0.12100 0.06730     0.547
## 9             Dodecanoic acid; 54     0.11700 0.07700     0.547
## 10              Nonanoic acid; 67    -0.11700 0.07790     0.547
## 11           Myo inositol 6TMS; 1    -0.11400 0.08390     0.547
## 12        2-Palmitoylglycerol; 39     0.11200 0.09100     0.547
## 13                Lactic acid; 29     0.11100 0.09490     0.547
## 14               Ribonic acid; 72    -0.10000 0.13000     0.670
## 15         Glutamic acid, 3TMS; 8    -0.09710 0.14200     0.670
## 16 3,4-Dihydroxybutanoic acid; 27    -0.09490 0.15200     0.670
## 17  2-hydroxy Isovaleric acid; 38    -0.09300 0.16000     0.670
## 18           Isoleucine, 2TMS; 18    -0.08930 0.17700     0.670
## 19              Hydroxylamine; 62     0.08820 0.18200     0.670
## 20     4-Hydroxybutanoic acid; 43    -0.08600 0.19400     0.670
## 21          Aminomalonic acid; 45    -0.08510 0.19800     0.670
## 22                    Ribitol; 70    -0.08210 0.21500     0.670
## 23              L-5-Oxoproline; 63    -0.08170 0.21700     0.670
## 24             Arabinopyranose; 51    -0.08130 0.22000     0.670
## 25              Glycine, 3TMS; 17    -0.08060 0.22300     0.670
## 26                Bisphenol A; 48    -0.07820 0.23700     0.685
## 27             Oleic acid, TMS; 3    -0.07420 0.26200     0.703
## 28             Hydroxyproline; 64    -0.07410 0.26300     0.703
## 29             Arachidic acid; 46     0.07220 0.27500     0.712
## 30               Serine, 3TMS; 14    -0.07080 0.28500     0.712
## 31        3-Indoleacetic acid; 40    -0.06880 0.29900     0.718
## 32           Malic acid, 3TMS; 11     0.06630 0.31700     0.718
## 33                   Glycerol; 57    -0.06540 0.32300     0.718
## 34 alpha-ketoglutaric acid, TMS M     0.06510 0.32600     0.718
## 35     3-Indolepropionic acid; 41     0.06120 0.35500     0.759
## 36              Proline, 2TMS; 21    -0.06010 0.36400     0.759
## 37 2-Hydroxybutyric acid, 2TMS; 2    -0.05480 0.40700     0.809
## 38           Cholesterol, TMS; 23    -0.05450 0.41000     0.809
## 39              Octanoic acid; 68     0.05290 0.42400     0.816
## 40      Arachidonic acid, TMS; 24    -0.05070 0.44400     0.832
## 41           Methionine, 2TMS; 16    -0.04940 0.45600     0.833
## 42          Heptadecanoic acid; 61    -0.04650 0.48200     0.861
## 43 4-Hydroxybenzeneacetic acid; 4    -0.04320 0.51400     0.881
## 44        Docosahexaenoic acid; 53     0.04130 0.53200     0.881
```

```
## 45         Benzeneacetic acid; 47    -0.04070 0.53900     0.881
## 46          Palmitic acid, TMS; 5    -0.03830 0.56300     0.881
## 47 4-Hydroxyphenyllactic acid; 44     0.03820 0.56400     0.881
## 48          Linoleic acid, TMS; 4    -0.03720 0.57400     0.881
## 49         Glyceryl-glycoside; 59    -0.03690 0.57800     0.881
## 50              Decanoic acid; 52     0.03580 0.58900     0.881
## 51           Myristoleic acid; 65    -0.03480 0.59900     0.881
## 52              Glyceric acid; 30     0.03010 0.64900     0.931
## 53 3-Hydroxybutyric acid, 2TMS; 1    -0.02910 0.66100     0.931
## 54         11-Eicosenoic acid; 35     0.02630 0.69100     0.931
## 55           Citric acid, 4TMS; 6    -0.02570 0.69800     0.931
## 56          Pyroglutamic acid; 69    -0.02480 0.70800     0.931
## 57               Ethanolamine; 56     0.02440 0.71200     0.931
## 58           Stearic acid, TMS; 2     0.02370 0.72000     0.931
## 59                   Tyrosine; 75    -0.02010 0.76200     0.952
## 60     Eicosapentaenoic acid; 55      0.01840 0.78100     0.952
## 61                1-Dodecanol; 36    -0.01700 0.79700     0.952
## 62             Tartronic acid; 73     0.01680 0.79900     0.952
## 63          Nonadecanoic acid; 66    -0.01510 0.81900     0.952
## 64                   Glycerol; 58    -0.01440 0.82700     0.952
## 65               Pyruvic acid; 31     0.01390 0.83400     0.952
## 66             1-Monopalmitin; 37    -0.01330 0.84100     0.952
## 67               Campesterol; 49      0.01150 0.86300     0.952
## 68          alpha-Tocopherol; 26     -0.01120 0.86600     0.952
## 69         Succinic acid, 2TMS; 7    -0.01040 0.87600     0.952
## 70            1,3-Propanediol; 34     0.00917 0.89000     0.953
## 71         Heptadecanoic acid; 60    -0.00461 0.94400     0.974
## 72                    Ribitol; 71     0.00392 0.95300     0.974
## 73       4-Deoxytetronic acid; 33    -0.00294 0.96500     0.974
## 74              Alanine, 2TMS; 25    -0.00288 0.96500     0.974
## 75          Fumaric acid, 2TMS; 9    -0.00215 0.97400     0.974
```

5.5.2.2 Forest Plot of Model Coefficients

```
## Warning: Ignoring unknown aesthetics: x

## Warning: Ignoring unknown aesthetics: x

## Warning: Ignoring unknown aesthetics: x

## Warning: Ignoring unknown aesthetics: x

## Warning: Ignoring unknown aesthetics: x

## Warning: Ignoring unknown aesthetics: x

## Warning: Ignoring unknown aesthetics: x

## NULL
```

5.5.3 Fully-Adjusted Model

```
## [1] "Fitting models:"
## [1] "~ SDNN + rest_HR_vag + Age + bmi + Blood_glucose + Duration_DM + Gender + Hba1c_baseline + log_
## [1] ""
```

5.5.3.1 Tables of Model Coefficients

```
## [1] ""
## [1] "Table: SDNN"
## [1] "  (from model: "
## [1] "   ~ SDNN + rest_HR_vag + Age + bmi + Blood_glucose +"
## [1] "     Duration_DM + Gender + Hba1c_baseline + log_Blood_TGA +"
## [1] "     Smoking + Statin + Total_cholesterol + egfr)"
## [1] ""
##                                Name Coefficient P.Value adj.P.Val
## 1               Valine, 2TMS; 20   -0.210000 0.00137     0.103
## 2              Leucine, 2TMS; 19   -0.152000 0.02060     0.563
## 3            Threonine, 3TMS; 12   -0.147000 0.02510     0.563
## 4           Tridecanoic acid; 74   -0.126000 0.05590     0.563
## 5        Phenylalanine, 2TMS; 13   -0.124000 0.05990     0.563
## 6           Isoleucine, 2TMS; 18   -0.120000 0.06740     0.563
## 7          Glutamic acid, 3TMS; 8   -0.117000 0.07340     0.563
## 8   2-hydroxy Isovaleric acid; 38   -0.117000 0.07400     0.563
## 9              Nonanoic acid; 67   -0.117000 0.07610     0.563
## 10       4-Deoxytetronic acid; 32   -0.114000 0.08120     0.563
## 11            Dodecanoic acid; 54    0.114000 0.08260     0.563
## 12        2-Palmitoylglycerol; 39    0.112000 0.09030     0.564
## 13              Hydroxylamine; 62    0.105000 0.11200     0.601
## 14               Serine, 3TMS; 14   -0.104000 0.11200     0.601
## 15                Lactic acid; 29    0.102000 0.12000     0.601
## 16 2-Hydroxybutyric acid, 2TMS; 2   -0.095600 0.14400     0.676
## 17           Aminomalonic acid; 45   -0.088200 0.17800     0.714
## 18           Malic acid, 3TMS; 11    0.087200 0.18500     0.714
## 19              L-5-Oxoproline; 63   -0.086100 0.19100     0.714
## 20                 Bisphenol A; 48   -0.082800 0.20900     0.714
## 21                    Glycerol; 57   -0.081500 0.21500     0.714
## 22     4-Hydroxybutanoic acid; 43   -0.078600 0.23300     0.714
## 23              Oleic acid, TMS; 3   -0.078200 0.23300     0.714
## 24             Arabinopyranose; 51   -0.076400 0.24200     0.714
## 25                  Creatinine; 50   -0.076300 0.24500     0.714
## 26           Cholesterol, TMS; 23   -0.074500 0.25400     0.714
## 27           Methionine, 2TMS; 16   -0.071400 0.27700     0.714
## 28                     Ribitol; 70   -0.069600 0.29000     0.714
## 29                     Ribitol; 71    0.067600 0.30000     0.714
## 30 alpha-ketoglutaric acid, TMS M    0.067100 0.30800     0.714
## 31              Glycine, 3TMS; 17   -0.066300 0.31400     0.714
## 32             Arachidic acid; 46    0.065300 0.32100     0.714
## 33 4-Hydroxyphenyllactic acid; 44    0.064300 0.32800     0.714
## 34      Arachidonic acid, TMS; 24   -0.063100 0.33800     0.714
## 35 2,4-Dihydroxybutanoic acid; 28   -0.062200 0.34000     0.714
## 36     3-Indolepropionic acid; 41    0.062400 0.34300     0.714
## 37         Heptadecanoic acid; 61   -0.054200 0.41000     0.822
## 38              Proline, 2TMS; 21   -0.053000 0.41900     0.822
## 39           Myo inositol 6TMS; 1   -0.050900 0.43400     0.822
## 40          Palmitic acid, TMS; 5   -0.050800 0.43900     0.822
## 41               Ribonic acid; 72   -0.049400 0.44900     0.822
## 42 3,4-Dihydroxybutanoic acid; 27   -0.043900 0.50100     0.886
## 43              Hydroxyproline; 64   -0.042700 0.51600     0.886
## 44                    Tyrosine; 75   -0.041200 0.53000     0.886
```

```
## 45        3-Indoleacetic acid; 40   -0.040500 0.53800     0.886
## 46      4-Deoxytetronic acid; 33    0.039300 0.54800     0.886
## 47         Benzeneacetic acid; 47   -0.038800 0.55500     0.886
## 48              Octanoic acid; 68    0.034000 0.60500     0.927
## 49          Linoleic acid, TMS; 4   -0.032900 0.61700     0.927
## 50           Myristoleic acid; 65   -0.032400 0.62200     0.927
## 51 3-Hydroxybutyric acid, 2TMS; 1   -0.031000 0.63700     0.927
## 52              Decanoic acid; 52    0.030500 0.64300     0.927
## 53         11-Eicosenoic acid; 35    0.029400 0.65500     0.927
## 54      Docosahexaenoic acid; 53    0.027000 0.68000     0.945
## 55          Nonadecanoic acid; 66   -0.022800 0.72900     0.985
## 56                1-Dodecanol; 36   -0.019900 0.76200     0.985
## 57             Tartronic acid; 73    0.019400 0.76700     0.985
## 58          Fumaric acid, 2TMS; 9    0.018100 0.78400     0.985
## 59           alpha-Tocopherol; 26   -0.014700 0.82200     0.985
## 60               Ethanolamine; 56    0.014300 0.82800     0.985
## 61              Glyceric acid; 30    0.013200 0.84100     0.985
## 62               Pyruvic acid; 31    0.011100 0.86600     0.985
## 63                Campesterol; 49    0.009490 0.88500     0.985
## 64            1-Monopalmitin; 37   -0.009430 0.88600     0.985
## 65           Citric acid, 4TMS; 6    0.008590 0.89600     0.985
## 66         Succinic acid, 2TMS; 7   -0.008460 0.89800     0.985
## 67            1,3-Propanediol; 34    0.008190 0.90100     0.985
## 68           Stearic acid, TMS; 2    0.006220 0.92500     0.985
## 69 4-Hydroxybenzeneacetic acid; 4    0.005520 0.93300     0.985
## 70     Eicosapentaenoic acid; 55    0.004930 0.94000     0.985
## 71         Glyceryl-glycoside; 59   -0.004840 0.94100     0.985
## 72         Heptadecanoic acid; 60   -0.003960 0.95200     0.985
## 73                   Glycerol; 58    0.002490 0.97000     0.985
## 74          Pyroglutamic acid; 69   -0.002310 0.97200     0.985
## 75              Alanine, 2TMS; 25   -0.000462 0.99400     0.994
```

5.5.3.2 Forest Plot of Model Coefficients

```
## Warning: Ignoring unknown aesthetics: x

## Warning: Ignoring unknown aesthetics: x

## Warning: Ignoring unknown aesthetics: x

## Warning: Ignoring unknown aesthetics: x

## Warning: Ignoring unknown aesthetics: x

## Warning: Ignoring unknown aesthetics: x

## Warning: Ignoring unknown aesthetics: x

## NULL
```

5.6 Neuropathy Questionnaire (mnsineuropat)

5.6.1 Crude Model

```
## [1] "Fitting models:"
## [1] "~ mnsineuropat"
## [1] ""
```

5.6.1.1 Tables of Model Coefficients

```
## [1] ""
## [1] "Table: mnsineuropat"
## [1] "  (from model: "
## [1] "   ~ mnsineuropat)"
## [1] ""
## [1] "No significant associations at p.adj < 0.05"
```

5.6.1.2 Forest Plot of Model Coefficients

```
## NULL
```

5.7 Adjusted Model

```
## [1] "Fitting models:"
## [1] "~ mnsineuropat + Age + bmi + Blood_glucose + Duration_DM + Gender + Hba1c_baseline + log_Blood_
## [1] ""
```

5.7.0.1 Tables of Model Coefficients

```
## [1] ""
## [1] "Table: mnsineuropat"
## [1] "  (from model: "
## [1] "   ~ mnsineuropat + Age + bmi + Blood_glucose + Duration_DM +"
## [1] "     Gender + Hba1c_baseline + log_Blood_TGA + Smoking + Statin +"
## [1] "     Total_cholesterol)"
## [1] ""
##                               Name Coefficient P.Value adj.P.Val
## 1                  Creatinine; 50     0.15300  0.0132     0.414
## 2            Myo inositol 6TMS; 1     0.14900  0.0154     0.414
## 3               Glycine, 3TMS; 17     0.14800  0.0166     0.414
## 4            Tridecanoic acid; 74    -0.13300  0.0305     0.509
## 5            Fumaric acid, 2TMS; 9    0.12500  0.0424     0.509
## 6                     Glycerol; 58     0.12100  0.0504     0.509
## 7                      Ribitol; 70     0.11500  0.0617     0.509
## 8             Malic acid, 3TMS; 11     0.11400  0.0643     0.509
## 9                  Bisphenol A; 48     0.11100  0.0717     0.509
## 10 4-Hydroxyphenyllactic acid; 44     0.11000  0.0735     0.509
## 11                Ribonic acid; 72     0.11000  0.0747     0.509
## 12               Decanoic acid; 52    -0.10500  0.0892     0.551
## 13        4-Deoxytetronic acid; 32     0.10300  0.0955     0.551
## 14              Oleic acid, TMS; 3     0.10000  0.1030     0.554
## 15            Stearic acid, TMS; 2     0.08600  0.1630     0.711
## 16             Tartronic acid; 73    -0.08240  0.1810     0.711
## 17              Proline, 2TMS; 21    -0.08170  0.1850     0.711
## 18             1,3-Propanediol; 34     0.07890  0.2000     0.711
## 19          Palmitic acid, TMS; 5     0.07830  0.2040     0.711
## 20 2,4-Dihydroxybutanoic acid; 28     0.07750  0.2080     0.711
## 21  2-hydroxy Isovaleric acid; 38     0.07750  0.2080     0.711
## 22          Glyceryl-glycoside; 59     0.07560  0.2200     0.711
## 23           Linoleic acid, TMS; 4     0.07420  0.2290     0.711
## 24 4-Hydroxybenzeneacetic acid; 4     0.07180  0.2440     0.711
## 25              Octanoic acid; 68    -0.07050  0.2520     0.711
## 26 3-Hydroxybutyric acid, 2TMS; 1     0.06980  0.2570     0.711
## 27 3,4-Dihydroxybutanoic acid; 27     0.06810  0.2690     0.711
## 28                    Ribitol; 71     0.06730  0.2740     0.711
## 29         Succinic acid, 2TMS; 7     0.06650  0.2810     0.711
## 30           alpha-Tocopherol; 26    -0.06550  0.2880     0.711
## 31           Citric acid, 4TMS; 6     0.06460  0.2940     0.711
## 32         11-Eicosenoic acid; 35     0.06230  0.3120     0.711
## 33         Heptadecanoic acid; 61     0.06050  0.3260     0.711
## 34           Isoleucine, 2TMS; 18    -0.05980  0.3320     0.711
## 35            Arabinopyranose; 51     0.05570  0.3660     0.711
## 36         Heptadecanoic acid; 60    -0.05530  0.3690     0.711
## 37           Methionine, 2TMS; 16    -0.05480  0.3730     0.711
## 38            Threonine, 3TMS; 12    -0.05420  0.3790     0.711
## 39           Nonadecanoic acid; 66     0.05370  0.3840     0.711
## 40          3-Indoleacetic acid; 40     0.05290  0.3900     0.711
## 41                Pyruvic acid; 31    -0.05220  0.3970     0.711
## 42     3-Indolepropionic acid; 41     0.05200  0.3980     0.711
## 43           Aminomalonic acid; 45     0.04770  0.4380     0.755
## 44              Alanine, 2TMS; 25     0.04730  0.4430     0.755
```

```
## 45          Myristoleic acid; 65     0.04540  0.4610     0.768
## 46               1-Dodecanol; 36    -0.04410  0.4740     0.768
## 47             Hydroxylamine; 62    -0.04340  0.4810     0.768
## 48             Glyceric acid; 30    -0.04230  0.4930     0.770
## 49             Leucine, 2TMS; 19    -0.03990  0.5170     0.791
## 50      Eicosapentaenoic acid; 55     0.03860  0.5300     0.796
## 51              Ethanolamine; 56    -0.03730  0.5450     0.799
## 52               Valine, 2TMS; 20    -0.03600  0.5590     0.799
## 53                   Tyrosine; 75    -0.03550  0.5650     0.799
## 54               Serine, 3TMS; 14     0.03390  0.5820     0.809
## 55            1-Monopalmitin; 37    -0.03270  0.5960     0.813
## 56 alpha-ketoglutaric acid, TMS M    -0.03140  0.6100     0.817
## 57          Pyroglutamic acid; 69     0.02790  0.6500     0.837
## 58         Benzeneacetic acid; 47     0.02740  0.6570     0.837
## 59        2-Palmitoylglycerol; 39    -0.02720  0.6590     0.837
## 60            Arachidic acid; 46     0.02600  0.6740     0.842
## 61        Phenylalanine, 2TMS; 13    -0.02380  0.6990     0.847
## 62                   Glycerol; 57    -0.02350  0.7030     0.847
## 63             Hydroxyproline; 64    -0.02230  0.7170     0.847
## 64 2-Hydroxybutyric acid, 2TMS; 2     0.02180  0.7230     0.847
## 65       4-Deoxytetronic acid; 33    -0.02070  0.7370     0.850
## 66             Nonanoic acid; 67     0.01550  0.8020     0.911
## 67     4-Hydroxybutanoic acid; 43     0.01320  0.8300     0.922
## 68      Arachidonic acid, TMS; 24    -0.01270  0.8360     0.922
## 69                Lactic acid; 29    -0.01150  0.8520     0.926
## 70         Glutamic acid, 3TMS; 8     0.01020  0.8690     0.931
## 71             L-5-Oxoproline; 63    -0.00845  0.8910     0.941
## 72          Cholesterol, TMS; 23     0.00735  0.9050     0.943
## 73       Docosahexaenoic acid; 53    -0.00380  0.9510     0.962
## 74                Campesterol; 49    -0.00362  0.9530     0.962
## 75            Dodecanoic acid; 54     0.00295  0.9620     0.962
```

5.7.0.2 Forest Plot of Model Coefficients

```
## Warning: Ignoring unknown aesthetics: x

## Warning: Ignoring unknown aesthetics: x

## Warning: Ignoring unknown aesthetics: x

## Warning: Ignoring unknown aesthetics: x

## NULL
```

5.8 Fully-Adjusted Model

```
## [1] "Fitting models:"
## [1] "~ mnsineuropat + Age + bmi + Blood_glucose + Duration_DM + Gender + Hba1c_baseline + log_Blood_
## [1] ""
```

5.8.0.1 Tables of Model Coefficients

```
## [1] ""
## [1] "Table: mnsineuropat"
## [1] "  (from model: "
## [1] "   ~ mnsineuropat + Age + bmi + Blood_glucose + Duration_DM +"
## [1] "     Gender + Hba1c_baseline + log_Blood_TGA + Smoking + Statin +"
## [1] "     Total_cholesterol + egfr)"
## [1] ""
##                              Name Coefficient P.Value adj.P.Val
## 1            Tridecanoic acid; 74   -1.41e-01  0.0211     0.654
## 2               Glycine, 3TMS; 17    1.33e-01  0.0295     0.654
## 3                 Bisphenol A; 48    1.11e-01  0.0698     0.654
## 4   2-hydroxy Isovaleric acid; 38    1.07e-01  0.0809     0.654
## 5                  Creatinine; 50    1.05e-01  0.0856     0.654
## 6                    Glycerol; 58    1.05e-01  0.0861     0.654
## 7             Stearic acid, TMS; 2    1.05e-01  0.0877     0.654
## 8               Oleic acid, TMS; 3    1.05e-01  0.0882     0.654
## 9               Decanoic acid; 52   -1.03e-01  0.0924     0.654
## 10                    Ribitol; 70    1.03e-01  0.0943     0.654
## 11          Fumaric acid, 2TMS; 9    1.02e-01  0.0960     0.654
## 12          Malic acid, 3TMS; 11    9.49e-02  0.1220     0.704
## 13             Proline, 2TMS; 21   -9.27e-02  0.1310     0.704
## 14          Palmitic acid, TMS; 5    9.25e-02  0.1310     0.704
## 15           Myo inositol 6TMS; 1    8.17e-02  0.1830     0.800
## 16             Tartronic acid; 73   -8.05e-02  0.1890     0.800
## 17 4-Hydroxyphenyllactic acid; 44    8.02e-02  0.1910     0.800
## 18            1,3-Propanediol; 34    7.84e-02  0.2010     0.800
## 19 3-Hydroxybutyric acid, 2TMS; 1    7.28e-02  0.2350     0.800
## 20       4-Deoxytetronic acid; 33   -6.93e-02  0.2580     0.800
## 21          Linoleic acid, TMS; 4    6.89e-02  0.2610     0.800
## 22               Serine, 3TMS; 14    6.84e-02  0.2650     0.800
## 23 2-Hydroxybutyric acid, 2TMS; 2    6.73e-02  0.2720     0.800
## 24         Heptadecanoic acid; 61    6.72e-02  0.2730     0.800
## 25         Succinic acid, 2TMS; 7    6.50e-02  0.2890     0.800
## 26           alpha-Tocopherol; 26   -6.12e-02  0.3190     0.800
## 27             Hydroxyproline; 64   -6.09e-02  0.3200     0.800
## 28          Nonadecanoic acid; 66    6.00e-02  0.3280     0.800
## 29         11-Eicosenoic acid; 35    5.91e-02  0.3350     0.800
## 30       4-Deoxytetronic acid; 32    5.87e-02  0.3390     0.800
## 31         Heptadecanoic acid; 60   -5.67e-02  0.3550     0.800
## 32               Ribonic acid; 72    5.66e-02  0.3560     0.800
## 33              Hydroxylamine; 62   -5.66e-02  0.3560     0.800
## 34     Eicosapentaenoic acid; 55    5.58e-02  0.3630     0.800
## 35    3-Indolepropionic acid; 41    5.42e-02  0.3770     0.807
## 36               Pyruvic acid; 31   -5.03e-02  0.4120     0.809
## 37          Aminomalonic acid; 45    4.98e-02  0.4170     0.809
## 38            Arabinopyranose; 51    4.94e-02  0.4210     0.809
## 39              Octanoic acid; 68   -4.83e-02  0.4310     0.809
## 40            Threonine, 3TMS; 12   -4.83e-02  0.4310     0.809
## 41           Myristoleic acid; 65    4.44e-02  0.4690     0.853
## 42                1-Dodecanol; 36   -4.35e-02  0.4780     0.853
## 43              Alanine, 2TMS; 25    4.13e-02  0.5000     0.856
## 44          Glyceryl-glycoside; 59    4.07e-02  0.5070     0.856
```

```
## 45             1-Monopalmitin; 37   -4.01e-02  0.5140     0.856
## 46           Methionine, 2TMS; 16   -3.41e-02  0.5780     0.906
## 47        Phenylalanine, 2TMS; 13   -3.32e-02  0.5890     0.906
## 48 alpha-ketoglutaric acid, TMS M   -3.21e-02  0.6010     0.906
## 49             Arachidic acid; 46    3.08e-02  0.6150     0.906
## 50         Glutamic acid, 3TMS; 8    3.01e-02  0.6230     0.906
## 51           Citric acid, 4TMS; 6    2.94e-02  0.6310     0.906
## 52           Isoleucine, 2TMS; 18   -2.88e-02  0.6390     0.906
## 53           Cholesterol, TMS; 23    2.83e-02  0.6440     0.906
## 54        2-Palmitoylglycerol; 39   -2.76e-02  0.6520     0.906
## 55              Glyceric acid; 30   -2.50e-02  0.6830     0.926
## 56         Benzeneacetic acid; 47    2.38e-02  0.6980     0.926
## 57              Leucine, 2TMS; 19   -2.33e-02  0.7040     0.926
## 58               Ethanolamine; 56   -2.16e-02  0.7240     0.937
## 59        3-Indoleacetic acid; 40    2.04e-02  0.7400     0.941
## 60 4-Hydroxybenzeneacetic acid; 4    1.89e-02  0.7590     0.948
## 61                   Tyrosine; 75   -1.66e-02  0.7870     0.967
## 62 2,4-Dihydroxybutanoic acid; 28    1.55e-02  0.8000     0.968
## 63 3,4-Dihydroxybutanoic acid; 27    1.45e-02  0.8130     0.968
## 64              Nonanoic acid; 67    1.32e-02  0.8300     0.973
## 65      Docosahexaenoic acid; 53    1.06e-02  0.8630     0.982
## 66               Valine, 2TMS; 20   -1.05e-02  0.8640     0.982
## 67            Dodecanoic acid; 54    7.29e-03  0.9050     0.999
## 68                   Glycerol; 57   -5.06e-03  0.9340     0.999
## 69     4-Hydroxybutanoic acid; 43    4.76e-03  0.9380     0.999
## 70           Pyroglutamic acid; 69    4.42e-03  0.9430     0.999
## 71                Campesterol; 49   -3.91e-03  0.9490     0.999
## 72             L-5-Oxoproline; 63   -3.11e-03  0.9600     0.999
## 73                    Ribitol; 71   -6.43e-04  0.9920     0.999
## 74                Lactic acid; 29    5.49e-04  0.9930     0.999
## 75      Arachidonic acid, TMS; 24    5.95e-05  0.9990     0.999
```

5.8.0.2 Forest Plot of Model Coefficients

```
## Warning: Ignoring unknown aesthetics: x

## Warning: Ignoring unknown aesthetics: x

## Warning: Ignoring unknown aesthetics: x

## Warning: Ignoring unknown aesthetics: x

## Warning: Ignoring unknown aesthetics: x

## Warning: Ignoring unknown aesthetics: x

## NULL
```

6 Appendix

```
## R version 3.6.2 (2019-12-12)
## Platform: x86_64-w64-mingw32/x64 (64-bit)
## Running under: Windows 10 x64 (build 17763)
##
## Matrix products: default
##
## locale:
## [1] LC_COLLATE=English_United States.1252
## [2] LC_CTYPE=English_United States.1252
## [3] LC_MONETARY=English_United States.1252
## [4] LC_NUMERIC=C
## [5] LC_TIME=English_United States.1252
##
## attached base packages:
## [1] stats     graphics  grDevices utils     datasets  methods   base
##
## loaded via a namespace (and not attached):
##  [1] Rcpp_1.0.3         plyr_1.8.5         pillar_1.4.3       compiler_3.6.2
##  [5] RColorBrewer_1.1-2 forcats_0.4.0      tools_3.6.2        digest_0.6.23
##  [9] evaluate_0.14      lifecycle_0.2.0    tibble_3.0.1       gtable_0.3.0
## [13] pkgconfig_2.0.3    rlang_0.4.6        yaml_2.2.0         haven_2.2.0
## [17] xfun_0.12          stringr_1.4.0      dplyr_0.8.3        knitr_1.27
## [21] vctrs_0.2.4        hms_0.5.3          grid_3.6.2         tidyselect_1.0.0
## [25] glue_1.3.1         R6_2.4.1           rmarkdown_2.1      limma_3.42.0
## [29] reshape2_1.4.3     ggplot2_3.2.1      readr_1.3.1        purrr_0.3.3
## [33] farver_2.0.3       tidyr_1.0.0        magrittr_1.5       scales_1.1.0
## [37] ellipsis_0.3.0     htmltools_0.4.0    assertthat_0.2.1   colorspace_1.4-1
## [41] labeling_0.3       stringi_1.4.4      lazyeval_0.2.2     munsell_0.5.0
## [45] crayon_1.3.4
```
