# Supplementary material for: Cardiovascular Autonomic Neuropathy in Type 1 Diabetes Is Associated With Disturbances in TCA, Lipid, and Glucose Metabolism
Source: Front Endocrinol (Lausanne). 2022 Apr 14;13:831793. doi: 10.3389/fendo.2022.831793 (PMC9046722; doi:10.3389/fendo.2022.831793)
Supplement: Supplementary file 1 [file DataSheet_1.zip › Supplementary List 1.DOCX]

Supplemtary list 1. List of metabolites and lipids

Metabolites

1-Dodecanol, 1-Monopalmitin, 1,3-Propanediol, 11-Eicosenoic acid, 2-hydroxy Isovaleric acid, 2-Hydroxybutyric acid, 2-Palmitoylglycerol, 2,4-Dihydroxybutanoic acid, 3-Hydroxybutyric acid, 3-Indoleacetic acid, 3-Indolepropionic acid, 3,4-Dihydroxybutanoic acid, 4-Deoxytetronic acid (A), 4-Deoxytetronic acid (B), 4-Hydroxybenzeneacetic acid, 4-Hydroxybutanoic acid, 4-Hydroxyphenyllactic acidAlanine, alpha-ketoglutaric acid MeOX, alpha-Tocopherol, Aminomalonic acid, Arabinopyranose, Arachidic acid, Arachidonic acid, Benzeneacetic acid, Bisphenol A, Campesterol, Cholesterol, Citric acid, Creatinine, Decanoic acid, Docosahexaenoic , cid, Dodecanoic acid, Eicosapentaenoic acid, Ethanolamine, Fumaric acid, Glutamic acid, Glyceric acid, Glycerol (A),Glycerol (B), Glyceryl-glycoside, Glycine, Heptadecanoic acid (A), Heptadecanoic acid (B), Hydroxylamine, Hydroxyproline, Isoleucine, L-5-Oxoproline, Lactic acid, Leucine, Linoleic acid, Malic acid, Methionine, Myo inositol, Myristoleic acid, Nonadecanoic acid, Nonanoic acid, Octanoic acid, Oleic acid, Palmitic acid, Phenylalanine, Proline, Pyroglutamic acid, Pyruvic acid, Ribitol (A), Ribitol (B), Ribonic acid, Serine, Stearic acid, Succinic acid, Tartronic acid, Threonine, Tridecanoic acid, Tyrosine,

Lipds

(Abreiviations: LPC = Lysophospholipid, PC= Phosphatidylcholine , SM= Sphingomyelin , TG= Triglyceride)

LPC(16:0), LPC(18:0), LPC(18:1), LPC(18:2), LPC(20:4), PC(16:0e/18:1(9Z), PC(32:0), PC(32:1), PC(32:2), PC(34:1), PC(34:2), PC(34:3), PC(35:1), PC(35:2), PC(36:2), PC(36:3), PC(36:4), PC(36:5), PC(38:2), PC(38:3), PC(38:4), PC(38:5), PC(38:6), PC(40:5), PC(40:6), PC(40:7), PC(O-34:2), PC(O-34:3), PC(O-36:4), PC(O-36:5), PC(O-38:4), PC(O-38:5), SM(42:2), SM(d16:1/18:1), SM(d18:1/24:0), SM(d18:2/24:1), SM(d32:1), SM(d33:1),SM(d34:1), SM(d36:1), SM(d36:2), SM(d38:1), SM(d38:2), SM(d39:1), SM(d40:1), SM(d40:2), SM(d41:1), SM(d41:2), TG(14:0/16:0/18:1), TG(14:0/18:1/18:1), TG(14:0/18:2/18:2), TG(16:0/18:0/18:1), TG(16:0/18:2/18:2), TG(16:0/18:2/18:3), TG(16:0/18:2/22:6), TG(16:0/22:5/18:1), TG(18:0/18:1/20:4), TG(18:1/12:0/18:1), TG(18:1/18:1/16:0), TG(18:1/18:1/18:1), TG(18:1/18:1/22:6), TG(18:1/18:2/18:2), TG(18:2/18:1/16:0), TG(18:2/18:1/18:1), TG(18:2/18:2/18:2), TG(18:2/22:5/16:0) ,TG(45:0), TG(46:0), TG(46:1), TG(48:3), TG(49:1), TG(50:0), TG(50:1), TG(50:2), TG(50:3), TG(51:2), TG(52:2), TG(52:3), TG(52:4), TG(54:2), TG(54:3), TG(54:4)
